# Diagnosis of hepatocellular carcinoma using liquid biopsy-based biomarkers: a systematic review and network meta-analysis

**DOI:** 10.3389/fonc.2024.1483521

**Published:** 2025-01-28

**Authors:** Yutong Jiang, Shangwen Qi, Rongrong Zhang, Ruixia Zhao, Yu Fu, Yuxuan Fang, Mingyi Shao

**Affiliations:** ^1^ The First Affiliated Hospital of Henan University of Chinese Medicine, Zhengzhou, Henan, China; ^2^ The First Clinical Medical College of Henan University of Chinese Medicine, Zhengzhou, China

**Keywords:** hepatocellular carcinoma, liquid biopsy, diagnostic biomarkers, network meta-analysis, liver disease

## Abstract

**Introduction:**

The diagnostic performance of liquid biopsy-based biomarkers for HCC was comprehensively compared in this network meta-analysis (NMA).

**Methods:**

A thorough literature search was conducted to identify all comparative studies from January 1, 2000, to January 11, 2024. The QUADAS-2 tool was utilized to appraise the quality of studies involving diagnostic performance. R (v4.3.3) and an ANOVA model-based NMA were used to assess the diagnostic accuracy of each biomarker.

**Results:**

This study included 82 studies comprising a total of 15,024 patients.CircRNA demonstrated significantly superior performance in distinguishing HCC from healthy populations (superiority index: 3.550 (95% CI [0.143-3])) compared to other diagnostic biomarkers for HCC. “mRNA exhibited significantly superior performance in distinguishing HCC from liver disease patients (superiority index:10.621 (95% CI [7-11])) compared to other diagnostic biomarkers for HCC. Further subgroup analysis of the top-ranking liquid biopsy-based diagnostic biomarkers revealed that hsa_circ_000224 (superiority index: 3.091 (95% CI[0.143-9]) ranked remarkably higher in distinguishing HCC from both healthy populations and liver disease patients. Subgroup analysis of mRNA demonstrated that KIAA0101 mRNA (superiority index: 2.434 (95% CI [0.2-5]) ranked remarkably higher in distinguishing HCC from healthy populations and liver disease patients, respectively.

**Discussion:**

The results of this meta-analysis show that circRNA and mRNA are the first choice for HCC diagnosis. Subsequent analysis of circRNA and mRNA highlighted hsa_circ_000224, hsa_circ_0003998, KIAA0101 mRNA and GPC-3mRNA as the optimal diagnostic biomarkers for distinguishing HCC from healthy populations and liver disease patients, respectively. Well-structured prospective studies are crucial to comprehensively validate these findings.

**Systematic Review Registration:**

https://www.crd.york.ac.uk/PROSPERO/,identifier CRD42024521299.

## 1 Introduction

Liver cancer ranks as the fifth most prevalent cancer globally and the fourth principal cause of cancer-related fatalities (1). In China, primary liver cancer stands as the fourth most prevalent malignant tumor and the second leading contributor to cancer-related death. Current therapeutic modalities for hepatocellular carcinoma (HCC) encompass surgery, transcatheter arterial chemoembolization, targeted therapy, and immunotherapy (2). Nonetheless, the overall survival rates for patients in advanced stages remain notably low, with a median survival period ranging between 10 to 20 months (3), and a 5-year survival rate ranging from 50% to 70% (4). Early detection and prompt treatment of liver cancer can notably augment life expectancy and diminish mortality (5–7). However, due to the lack of obvious early symptoms and reliable diagnostic biomarkers, 30% to 40% of HCC patients miss timely intervention. Presently, clinical diagnosis of HCC predominantly relies on imaging modalities such as abdominal ultrasound, MRI, and enhanced CT scans, as well as histology (8, 9). Serum alpha-fetoprotein (AFP) has long been used as an early diagnostic biomarker for HCC; however, its sensitivity and specificity remain inadequate (10, 11). Particularly for HCC tumors measuring less than 3 cm in diameter, AFP sensitivity diminishes significantly (12, 13), rendering its diagnostic accuracy subpar. This highlights an urgent need to develop highly sensitive and specific diagnostic biomarkers for HCC (14, 15).

Liquid biopsy, encompassing circulating free microRNA, circulating tumor cells, and circulating tumor DNA, utilized for analyzing plasma and other biological fluids such as saliva, urine, and feces, has demonstrated significant promise in the diagnosis of HCC (16). It may offer heightened sensitivity and specificity compared to serum AFP (17). Although various liquid biopsy-based biomarkers are available for diagnosing HCC, it remains unclear which offers the highest sensitivity and specificity. Existing studies have primarily compared liquid biopsy biomarkers to the gold standard (AFP) but lack direct comparisons between different liquid biopsy-based biomarkers. Hence, there is a critical need to identify the most sensitive and specific liquid biopsy-based biomarker for early diagnosis of HCC (18).

The lack of consensus on the diagnostic performance of different liquid biopsy-based biomarkers for HCC, combined with the limited patient numbers in single-center studies, compromises the generalizability of results. Network meta-analysis (NMA) offers a solution by amalgamating efficacy data from multiple studies assessing various treatments for a specific disease and estimating unobserved comparisons from the observed network of comparisons. Conventionally, NMA is employed to evaluate treatment interventions based on randomized controlled trials, rather than the accuracy of diagnostic tests. However, an ANOVA model based on Bayesian methods bridges this gap. Using this method, the log-transformed sensitivity and specificity can be expressed as a sum of fixed effects of the test (19). This method correlates study effects to simulate the inherent correlation between the sensitivity and specificity of various tests and random errors. Additionally, it incorporates the superiority index proposed by Deutsch et al. (20), which facilitates the ranking of diagnostic tests. Hence, the variance analysis model was applied to compare the performance of various liquid biopsy-based diagnostic methods in distinguishing HCC patients from healthy populations and liver disease patients, and to explore the optimal biomarker for diagnosing HCC.

## 2 Methods

Following the Preferred Reporting Items for Systematic Reviews and Meta-Analyses of Diagnostic Test Accuracy Studies (PRISMA-DTA) guidelines (21), this study was executed and registered with the PROSPERO for meta-analysis (ID: CRD42024521299).

### 2.1 Strategy adopted while searching

A search was made across PubMed, Embase, Cochrane Library, and Web of Science from January 1, 2000, to January 11, 2024, with language restricted to English. The search terms combined MeSH and free-text terms, encompassing keywords such as “Hepatitis”, “Liver Cirrhosis”, “Liquid Biopsy”, “Liver Cell Carcinoma”, “Carcinoma, Hepatocellular”, “Diagnosis”, among others. The detailed search strategy utilized is outlined in **Supplementary Table S1**.

### 2.2 Literature screening

Inclusion criteria: (1) Study participants: individuals diagnosed with HCC, aged 18 years or older; (2) Diagnostic approach: Utilization of liquid biopsy-based biomarkers, including circulating free DNA(cfDNA), microRNA(miRNA), long non-coding RNA(lncRNA), messenger RNA(mRNA), exosome, circular RNA(circRNA), and circulating tumor cell (CTC), for HCC detection; (3) Control (gold standard): Alpha-fetoprotein (AFP); (4) Outcome assessment: Acquisition or computability of adequate data, such as sensitivity (SEN), specificity (SPE), true positive (TP), false positive (FP), true negative (TN), and false negative (FN); (5) Study design: Cross-sectional, cohort, case-control.

The diagnostic threshold for HCC is defined as an AFP level ≥ 500 μg/L or 400 ng/mL. HCC diagnosis was established based on either: (1) liver transplantation pathology, or (2) identification of a new mass exceeding 1 cm in size in the context of cirrhosis, detected via Computed Tomography (CT), Magnetic Resonance Imaging (MRI), or angiographic imaging showing arterial enhancement and portal-vein washing.

Exclusion criteria: (1) Reviews, meta-analyses, conference abstracts, editorials, letters, case reports, comments, brief surveys, and notes; (2) Animal or *in vitro* studies; (3) Duplicated or unavailable literature; (4) Studies lacking extractable outcome measures; (5) Literature containing apparent data errors; (6) Literature with a sample size of fewer than 30 cases.

Following the preset criteria, two reviewers, J.Y.T and Z.R.R, independently selected the studies. Initially, all potentially relevant studies were imported into EndNote X9 for duplicate exclusion. Subsequently, titles and abstracts were screened to exclude ineligible ones. Finally, full texts were further screened. Disagreements were resolved through discussion with a third researcher, Z.R.X.

### 2.3 Data extraction and quality assessment

Two reviewers, J.Y.T and Z.R.R, independently extracted data from the finally included studies, encompassing details such as first author, publication year, country, basic demographic information of study subjects, test methodologies, test parameters, cutoff values, SPE, SEN, TP, FP, FN, and TN, either directly provided or calculable from source data. Discrepancies were resolved through dialogue or, if necessary, by a third reviewer, Z.R.X.

The quality and applicability of the included studies on diagnostic accuracy were evaluated using the QUADAS-2 tool (22) by two independent reviewers, J.Y.T and Z.R.R. This tool comprises four domains assessing the risk of bias (patient selection, index test, gold standard, patient flow and timing) and three applicability domains (patient selection, index test, reference standard). A domain was considered to have a low risk of bias if all key questions were answered “yes”; conversely, any “no” response indicated a high risk of bias. When information was insufficient, the risk of bias was considered unclear. Discrepancies were resolved through discussion between the J.Y.T and Z.R.R, and if, consensus was not achieved, Z.R.X made the final decision. The quality assessment of the included studies was performed by RevMan 5.4 (The Cochrane Collaboration).

### 2.4 Data synthesis and statistical analysis

The values of TP/FP/FN/TN were extracted from each study. If the included studies reported SEN, SPE, the number of HCC patients, and the number of cases with other liver diseases, a 2×2 table for diagnostic tests was used to calculate TP/FP/FN/TN.

A Bayesian network meta-analysis (NMA-DT) of diagnostic test accuracy data was conducted using the R v4.3.3 (The R Foundation). In addition, an Analysis of Variance(ANOVA) analysis model (19) was employed to evaluate the accuracy of each liquid biopsy-based biomarker in diagnosing HCC, even when different thresholds were employed. The model represents the logit-transformed sensitivity and specificity as the sum of the fixed effects of the detection method, the associated study effects to model the intrinsic correlation between sensitivity and specificity, and the random errors associated with various detection methods evaluated in specific studies. At the same time, the superiority index proposed by Deutsch et al. (23) was incorporated. This index can be used to rank diagnostic tests. With this method, the diagnostic tests were ranked by calculating the relative superiority index, which integrates sensitivity and specificity. The expression for the superiority index is as follows:


$$\text{Superiority }{\text{index}}_{\text{k}}=\frac{2{\text{a}}_{\text{k}}+{\text{c}}_{\text{k}}}{2{\text{b}}_{\text{k}}+{\text{c}}_{\text{k}}}$$

where a_k_ is the number of tests to which test k is superior (higher sensitivity and specificity); b_k_ is the number of tests to which test k is inferior (lower sensitivity and specificity); and c_k_ is the number of tests exhibiting the same performance as test k (equal sensitivity and specificity).

Compared to the ranking method based on the diagnostic odds ratio (DOR), this approach is particularly suitable for diagnostic tests with high sensitivity but low specificity or low sensitivity but high specificity. In general, higher values of the DOR and relative superiority index denote increased accuracy in disease detection. Also, receiver operating characteristic curves (ROC) and area under the curve (AUC) values for each diagnostic test were plotted and computed. Subgroup analyses were conducted for top-ranked biomarkers. Subgroup analyses for each biomarker involved distinguishing HCC patients from healthy individuals and those with liver disease.

The above modeling method, grid plots of diagnostic tests, sensitivity analysis, and heterogeneity assessment were conducted using R (v4.3.3).

## 3 Results

### 3.1 Results and flowchart for literature screening

**Figure 1:**
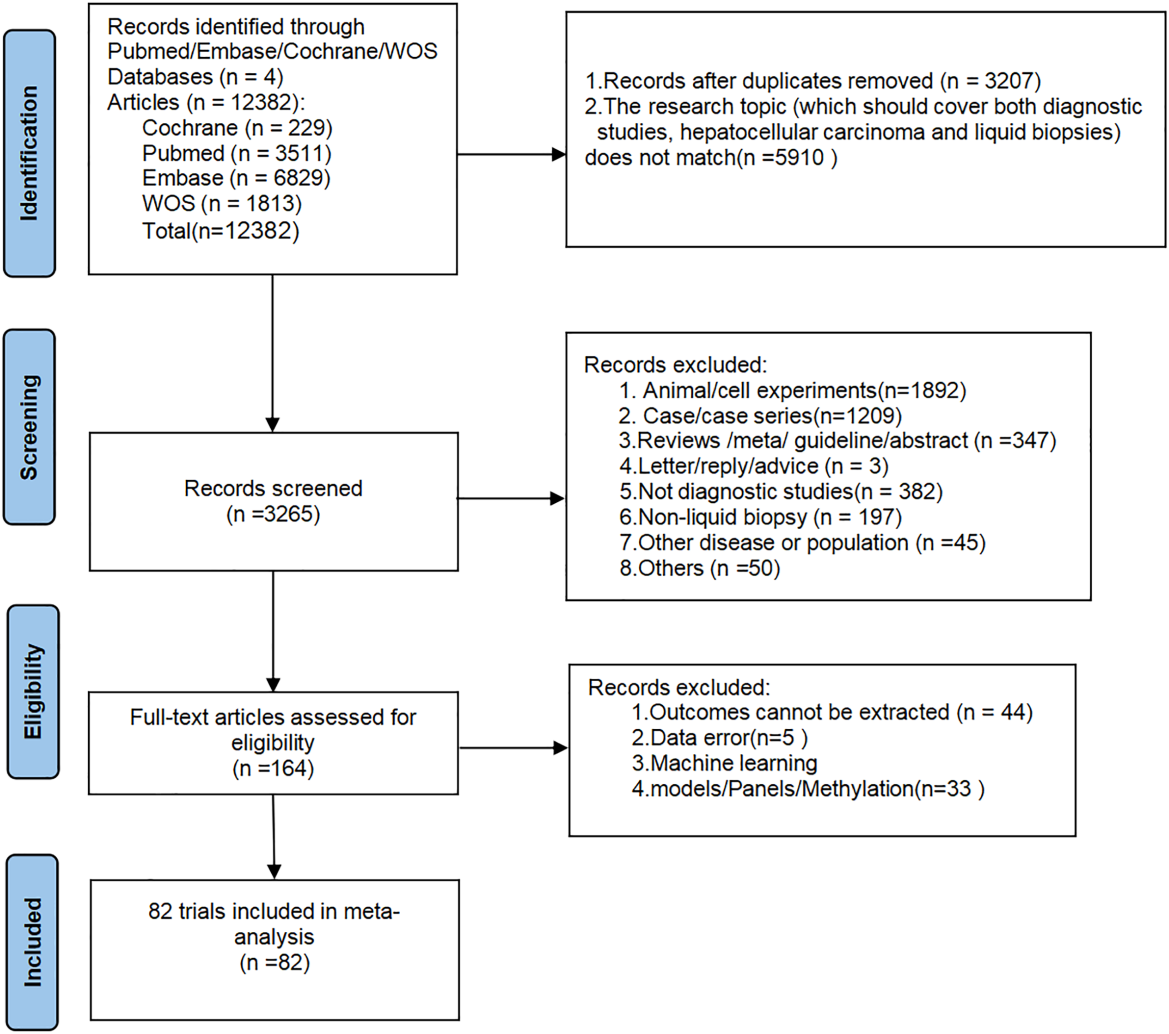
Diagram of literature screening.

A total of 12,382 articles were initially retrieved from databases. After removing 3,207 duplicate records and 5,910 articles unrelated to the research topic (not simultaneously meeting the criteria of “diagnostic studies + hepatocellular carcinoma + liquid biopsy”), 3,265 articles remained. These 3,265 articles were then screened by title and abstract, resulting in the exclusion of 1,892 articles based on animal or cell experiments, 1,209 case or case series reports, 347 review/guideline/meta-analysis articles, 382 articles unrelated to diagnostics, 197 articles unrelated to liquid biopsy, 45 articles on other diseases, 3 correspondence/suggestions articles, and 50 other unrelated articles. A total of 164 articles underwent detailed full-text review, during which 44 articles were excluded due to the inability to extract the required data, 5 articles were excluded due to obvious data errors, and 33 articles were excluded for being on topics such as machine learning, panels, or DNA methylation. Finally, a total of 82 studies were included in this meta-analysis (**Supplementary Table S2**). The literature screening process is depicted in **Figure 1**.

### 3.2 Basic characteristics of included studies

The 82 included studies originated from 10 countries (China, Egypt, Japan, Thailand, India, Vietnam, Saudi Arabia, Germany, Italy, and Turkey), encompassing a total of 15,024 patients. Gender information was missing in 21 studies, resulting in a total of 7,484 males and 3,428 females across the remaining studies. Age information was missing in some studies, and the methods of recording varied, yielding an overall age range of 22 to 101 years. In differentiating HCC from healthy populations, 47 studies incorporated five types of liquid biopsy-based biomarkers: exosome, lncRNA, mRNA, miRNA, and circRNA. As for distinguishing HCC from liver disease patients, 67 studies encompassed six types of liquid biopsy-based biomarkers: exosome, lncRNA, mRNA, miRNA, circRNA, and cfDNA. The basic characteristics of the included studies are outlined in **Table 1**, and the PCR detection method is shown in **Supplementary Table S3**.

**Table 1:** Main characteristics of included studies.

No.	First Author	Publication Year	Country	Study Design	*N* (Male/Female)	Control group	Age Mean ± SD or Medianor Median or range	Sample source	Liquid biopsy biomarker	Detection Method	Main index
1	Chen	2015	China	cohort study	HCC:104(68/32) CHB:100(66/34) LC:90(58/32) hc:120(80/40)	CHB/LC/ hc	HCC:47.6(33,64) CHB:48.9(35,62) LC:46.1(36,60) hc:47.9(34,59)	Serum	miR-96	RT-PCR	sensitivity/specificity/AUC
2	Guo	2021	China	case control study	HCC:87 Gastric cancer:13 cholangiocarcinoma:15 hc:30	Gastric cancer/ cholangiocarcinoma/ hc	N/A	plasma	Circular RNA 0006602	qPCR	sensitivity/specificity/AUC
3	Yosry	2022	Egypt	observational cross-sectional study	Early Fibrosis:80(48/32) Advanced Liver Fibrosis:77(42/35) HCC:62(42/15)	Early Fibrosis/ Advanced Liver Fibrosis	Age: median Early Fibrosis:49(43,57) Advanced Liver Fibrosis:55(50,61) HCC:60(57,65)	Serum	miR-141-3p/miR-155-5p/miR200a-3p miR-200c-3p, miR-/205-5p/miR-208a-3p, miR-499a-5p, miR-103a-3p, miR-574-3p and miR-15a-5p	RT-PCR	sensitivity/specificity/cut -off/accuracy/AUROC/LR+/LR-
4	Hung	2015	China Taiwan	case control study	HCC: 120(96/24) DN: 30(24/6) hc:15(12/3)	DN/hc	HCC: 58.5 ± 10.0 DN: 60.3 ± 11.1 hc:62.1 ± 8.7	serum	miR-122/let-7b	qRT-PCR	sensitivity/specificity/cut-off point/AUC
5	Gwad	2018	Egypt	case control study	HCC:60(43/17) CHC:42(33/9) hc:18(12/6)	CHC/hc	≥57.8;<57.8(numbers) HCC:35/25 CHC: 21/21 hc:9/9	serum	lncRNA‐RP11‐513I15.6/ miR‐1262/RAB11A mRNA	qRT-PCR	sensitivity/specificity/ PPV/NPV/accuracy/cut off
6	Matboli	2018	Egypt	N/A	HCC:68(49/19) CHC:60(39/21) hc:36(23/13)	CHC/ hc	<56;≥56(numbers) HCC:23/45 CHC:28/32 hc:12/24	serum	hsa_circ_00156/ hsa_circ _000224/hsa_circ_000520	Real‐time PCR	sensitivity/specificity/ PPV/NPV/accuracy
7	Li	2012	China	case control study	HCC:101(76/25) CH and LC::30(23/7) hc:60(46/14)	CH and LC/ hc	HCC:54 ± 11 CH and LC:51 ± 13 hc:52 ± 16	serum	miR-18a	qPCR	sensitivity/specificity/cut-off point/AUC
8	Rashad	2017	Egypt	case control study	HCC: 51(31/20) LC: 39(18/21)	LC	HCC:45.94 ± 6.42 LC: 48.28 ± 5.04	serum	miRNA-27a/ miRNA-18b	qRT-PCR	sensitivity/specificity/cut-off point/AUC
9	Habib	2019	Egypt	case control study	HCC:65(46/19) CHC:34(27/7) hc:32(17/15)	CHC/ hc	≥56;<56(numbers) HCC:43/22 CHC: 22/12 hc: 15/17	serum	lncRNA-TSIX/miR-548-a-3p/SOGA1 mRNA	qPCR	sensitivity/specificity/cut-off point/AUC
10	EI-Hamouly	2019	Egypt	cross-sectional study	HCC:42(27/15) LC:48(29/19) hc:40(23/17)	LC/ hc	HCC:56.14 ± 8.67 LC:53.85 ± 6.37 hc:53.35 ± 5.71	Plasma	microRNA-301	qRT-PCR	sensitivity/specificity/cut-off point/AUC
11	EIhendawy	2020	Egypt	cross section pilot study	HCC:20(11/9) hc:10(6/4)	hc	HCC:54.93 ± 7.49 hc:51.72 ± 4.72	peripheral venous blood	miR-142-5p/miR-191-5p/miR-22-3p/miR-126-5p	RT-qPCR	sensitivity/specificity/ PPV/NPV/AUC
12	Piciocchi	2013	Italy	N/A	HCC:66(50/16) LC:35(21/14) CH:41(28/13)	LC/ CH	HCC:68.5 ± 9 LC:58 ± 13 CH:57 ± 12	Plasma	cfDNA	Real-time PCR	sensitivity/specificity/ PPV/NPV/AUC/accuracy/cut-off point
13	Yang	2015	China	case control study	HCC:156(111/45) BLD:98(CH:83, NAFLD:15) hc:64	BLD/ hc	HCC: Mean age:53.7	serum	miR-218	qRT-PCR	sensitivity/specificity/cut-off point/AUC
14	Huang	2011	China	case control study	HCC:72 BLD:37,(LC:25, Chronic inactive CH:12)/ hc: 41	BLD:(LC, Chronic inactive CH)/ hc	N/A	Plasma	circDNA	Real Time PCR	sensitivity/specificity/ PPV/NPV/AUC/cut-off point
15	Chen	2015	China	case control study	HCC:103(89/14) BLD:95(80/15) hc:40(31/9)	BLD: hc:	Median (range) HCC:52(39-80) BLD:50(37-76) hc:49(30-78)	Serum	miR-182/miR-331-3p	real-time PCR	sensitivity/specificity/ PPV/NPV/AUC/cut-off point
16	Zuo	2015	China	N/A	HCC:90(68/22) CHB: 30(13/17) hc: 30(11/19)	CHB, hc	HCC:54.7 ± 9.8 CHB: 46.6 ± 15.4 hc: 51.8 ± 20.2	Serum	miR-125b/miR-223/miR-27a/miR26a	qRT-PCR	sensitivity/specificity/ AUC/cut-off point
17	Nasser	2019	Egypt	N/A	HCC:30(16/14) LC:15(11/4) CHC:15(9/6) hc: 25	LC/ CHC/ hc	HCC:53.5 ± 4.9 LC:51 ± 5.7 CHC:32 ± 7.4 hc:N/A	serum	miR-21/miR-223/miR-885-5p	(RT) PCR	sensitivity/specificity/ AUC/cut-off point
18	Fouda	2020	Egypt	case control study	HCC:137(93/44) hc:49	hc	HCC:61.1 ± 6.5 hc:60 ± 5.2	serum	miRNA21/miRNA29a/miRNA200/ miRNA335	qRT-PCR	sensitivity/specificity/ AUC/cut-off point/SEM
19	Li	2019	China	case control study	HCC:135 hc:103 CH:117 LC:143	hc/ CH/ LC	N/A	plasma	circSMARCA5	qRT-PCR	sensitivity/specificity/ AUC/cut-off
20	Abdelgawad	2015	Egypt	case control study	HCC:77(50/27) LC:30(19/11) hc:40(24/16)	LC/ hc	HCC:61(47-77) LC:58.5(55-64) hc:45-69	whole blood	KIAA0101 mRNA	qRT-PCR	sensitivity/specificity/ PPV/NPV/accuracy/
21	Zhuang	2015	China	case control study	HCC:52(40/12) CH:42(27/15) HC:43(23/14)	CH/ HC	HCC:51.1 ± 1.4 CH:49.2 ± 1.8 HC:51.5 ± 2.1	serum	miR-21/miR-26a/miR-101	qRT-PCR	sensitivity/specificity/ AUC
22	Lin	2015	China	Cohort	HCC:26(20/6) LC:22(16/6) CHB:23(16/7) hc:22(15/7)	LC/ CHB/ hc	HCC:49 ± 13 LC:51 ± 11 CHB:45 ± 11 hc:47 ± 12	Serum	miR-224	qRT-PCR	sensitivity/specificity/ AUC
23	Han	2018	China	single centre, cohort study	HCC:155(127/28) LC:96(64/32) hc: 95(61/34)	LC/ hc	HCC:58.2 ± 10.5 LC: 52.4 ± 11.4 hc: 51.1 ± 13.7	plasma	miR-148a	qRT-PCR	sensitivity/specificity/ AUROC/cut-off point
24	Sun	2019	China	case control study	HCC:40(26/14) hc:45(26/19) Anhepatic fibrosis:13(8/5) Mild hepatic fibrosis:46(29/17) Severe liver fibrosis/LC:47(29/18)	hc/ Anhepatic fibrosis/ Mild hepatic fibrosis/ Severe liver fibrosis/LC	HCC:62.53 ± 9.92 hc:44.61 ± 14.95 Anhepatic fibrosis:36.79 ± 11.8 Mild hepatic fibrosis:45.1 ± 12.94 Severe liver fibrosis/LC:54.56 ± 7.75	Serum	miR-331-3p/miR-23b-3p	RT-qPCR	sensitivity/specificity/ AUC
25	Amr	2017	Egypt	case control study	HCC:40(33/7) CHC:40(34/6) hc:20(16/4)	CHC/ hc	HCC:52.03 ± 1.55 CHC:48.94 ± 1.34 hc:50.75 ± 1.8	plasma	miR-122/miR-224	RT-quantitative PCR	sensitivity/specificity/ accuracy/cut-off point/AUC
26	Yang	2017	China		HCC:24(19/5) Fibrosis:62(46/16)	Fibrosis	HCC:57.7 ± 8.7 Fibrosis:35.7 ± 10.1	plasma	cfDNA	Qubit dsDNA 7 HS Assay kit	sensitivity/specificity/ PPV/NPV/cut-off point/AUC
27	Tomimaru	2011	Japan	case control study	HCC:126(99/27) CH:30(20/10) hc:50(37/13)	CH/ hc	HCC: 63 ± 10 CH:62 ± 8 hc:62 ± 8	Plasma	microRNA-21	qRT-PCR	sensitivity/specificity/ AUC/accuracy
28	Shaheen	2018	Egypt	case control study	HCC:40(26/14) LC:20(15/5) CHC:20(13/7) hc:40(34/6)	LC/ CHC/ hc	HCC:58.0 ± 8.6 LC:57.2 ± 9.7 CHC:52.5 ± 9.04 hc:57.5 ± 10.0	Serum	miR-182/miR-150	qRT-PCR	sensitivity/specificity/cut-off point/AUC
29	Luo	2018	China	case-control study	HCC:60(50/10) CHB and LC:75(59/16) hc:79(60/19)	CHB and LC/ hc	N/A	Plasma	ZFAS1	RT-qPCR	sensitivity/specificity/cut-off point/AUC
30	Li	2021	China	case control study	HCC:116(62/54) CHB:66(35/31) hc:66(33/33)	CHB/ hc	HCC:56.12 ± 5.46 CHB:57.81 ± 5.69 hc:56.88 ± 6.23	serum	miR-487b	qRT-PCR	sensitivity/specificity/cut-off point/AUC
31	ALrefai	2023	Saudi Arabia	case control study	HCC:50(41/9) LC:50(38/12) hc:50(35/15)	LC/ hc	HCC:59.74± 6.16 LC: 57.68± 4.77 hc: 58.78± 4.03	blood	miR-331-3P/miR-23b-3p/miR-3194-5p	RT-PCR	sensitivity/specificity/ PPV/NPV/AUC/cut-off point
32	Zhao	2021	China	case control study	HBV-unrelated HCC:52 CHB-related HCC:96 CHB:72 hc:76	CHB/ hc	HBV-unrelated HCC:52(43-62) CHB-related HCC:55(41-67) CHB:50(38-61) hc:53(42-59)	serum	miR-324-3p	qRT-PCR	sensitivity/specificity/cut-off point/AUC
33	Chen	2016	China	case control study	HCC:64(53/11) LC:59(44/15) CHB:63(51/12) hc:56(39/17)	LC/ CHB/ hc	HCC:54.00 ± 11.48 LC:52.93 ± 11.37 CHB:53.62 ± 11.04 hc:53.07 ± 11.83	Plasma	miR-125b	qRT-PCR	sensitivity/specificity/AUC
34	Huang	2020	China	case control study	HCC:129(111/18) hc:93(69/24) CHB:27(21/6) LC:49(32/17)	hc/ CHB/ LC	HCC:59(23-88) hc:55(21-79) CHB:52(33-71) LC:58(35-87)	Serum	HULC/MALAT1/Linc00152/PTENP1/PTTG3P/SPRY4-IT1/UBE2CP3/UCA1	qRT-PCR	sensitivity/specificity/AUC
35	Miura	2005	Japan	N/A	HCC:64 LC:20 CH:20 hc:50	LC/ CH/ hc	N/A	Serum	hTERT mRNA	qRT-PCR	sensitivity/specificity/ PPV/NPV/AUC/cut-off point
36	Shen	2017	China	case control study	HCC:70(46/24) LC:40(24/16) hc:45	LC/ hc	HCC:mean:35(42-72) LC:48(37-69) hc:49(40-68)	Serum	miR-574-3p	RT-qPCR	sensitivity/specificity/ PPV/NPV/AUC/accuracy
37	Nguyen	2022	Viet Nam	cross-sectional study	HCC:170(136/34) LC:170(90/80)	LC	HCC:59.8 ± 11.3 LC:46.5 ± 13.8	Serum	hTERT mRNA	RT-PCR	sensitivity/specificity/AUC/cut-off
38	Miura	2010	Japan	case control study	HCC:303(196/107) CH:89 LC:45 hc:201	CH/ LC/ hc/	HCC:mean:65;range(22-101)	Serum	hTERTmRNA	RT-qPCR	sensitivity/specificity/ PPV/NPV/AUC/cut-off
39	Zhang	2014	China	case control study	HCC:95(60/35) CH:118(65/33) hc:127(71/56)	CH/ hc	HCC:54.21 ± 6.95 CH:53.12 ± 7.24 hc:52.58 ± 6.98	Serum	microRNA 143/microRNA 215	RT-qPCR	sensitivity/specificity/ AUC/cut-off/accuracy
40	Han	2022	China	case control study	HCC:127(81/46) hc:99(48/51) BLD:55(30/25)	hc/ BLD	HCC:56(45-65) hc:53(42-59) BLD:50(39-59)	Serum	long non-coding RNA SCARNA10	qPCR	sensitivity/specificity/AUC
41	Yu	2015	China	N/A	HCC: 120(75/45) CHB:110(67/43) hc: 120(65/55)	CHB/ hc	HCC:58 ± 10.4 CHB: 55 ± 11.2 hc:50 ± 9.5	Serum	microRNA-150	qRT-PCR	sensitivity/specificity/ AUC/cut-off
42	Dhayat	2015	Germany	case control study	HCC: 22(20/2) LC: 22(13/9) hc: 15(7/8)	LC/ hc	N/A	plasma	microRNA-141/microRNA-200a	qRT-PCR	sensitivity/specificity/AUC/accuracy
43	Li	2019	China	N/A	HCC:47(31/16) hc: 54(23/31) CH: 54(31/23) LC: 35(22/13)	hc/ CH/ LC	HCC:63 ± 10 hc:45 ± 15 CH:37 ± 12 LC:55 ± 8	Serum	GPC3/miR-122	qRT-PCR	sensitivity/specificity/ AUC/Cut-off
44	Gharib	2022	Saudi Arabia	case control study	HCC:55(33/22) LC:55(31/24) hc:55(37/18)	LC/ hc	HCC:54.83 ± 6.54 LC:54.91 ± 8.69 hc:55.40 ± 7.24	Serum	miRNA-96-5p/miRNA-99a-5p	qRT-PCR	sensitivity/specificity/ PPV/NPV/AUC/cut-off/accuracy
45	Qiao	2019	China	N/A	HCC:100 CHB:50 hc:50	CHB/ hc	N/A	plasma	Hsa_circ_0003998	qRT-PCR	sensitivity/specificity/AUC
46	Quoc	2018	Vietnam	case control study	HCC: 25(17/8) CHB: 8(3/5) hc:19(13/6)	CHB/ hc	HCC: 51.28 ± 16.03 CHB: 41.13 ± 12.92 hc:55.79 ± 7.8	plasma	hsa-miR122	RT-PCR	sensitivity/specificity/AUC
47	Wang	2018	China	N/A	HCC: 63(54/9) CHB: 46(34/12) LC: 47(39/8) hc:58(49/9)	CHB/ LC/ hc	HCC:56 ± 9 CHB:54 ± 12 LC:55 ± 10 hc:55 ± 10	plasma	lncRNA GAS5-AS1	RT-qPCR	sensitivity/specificity/ AUC/Cut-off
48	Wang	2023	China	N/A	HCC:98(60/38) LC:52(28/24) hc:105(62/43)	LC/ hc	HCC: 56.3 ± 9.76 LC:57.98 ± 11.35 hc:54.8 ± 15.05	plasma	Lnc-MyD88	RT-qPCR	sensitivity/specificity/ AUC/Cut-off/Jorden index
49	Yousuf	2022	India	N/A	HCC:33(22/11) hc:33(23/10)	hc	HCC:56.58 ± 12.29 hc:51.68 ± 15.09	Serum	miR-221/miR-222/miR-26a/miR-124/miR-340/miR-126/miR-155miR-219	RT-qPCR	sensitivity/specificity/ AUC/Cut-off
50	Elfert	2022	Egypt	case control study	HCC:90(72/18) CHC:90(66/24) hc:60(35/25)	CHC/ hc	HCC: 47.43 ± 7.56 CHC: 43.40 ± 11.66 hc: 44.0 ± 7.32	Serum	miR-122/miR-483/miR-335	qRT-PCR	sensitivity/specificity/ AUC/Cut-off
51	Eldosoky	2023	Egypt	N/A	HCC:39(27/12) LC: 40(28/12)	LC	HCC: 61.0 (56.0–67.0) LC:58.5 (54.25–65.0)	plasma	hsa-miR-21-5p/hsa-miR-155-5p/hsa-miR-199a-5p	qRT-PCR	sensitivity/specificity/ AUC/Cut-off
52	Boonkaew	2023	Thailand	N/A	HCC:70(54/16) NAFLD:70(30/40) hc:35(4/31)	NAFLD/ hc	HCC:68.8 ± 11.4 NAFLD:50.7 ± 9.5 hc:53.2 ± 5.3	plasma	miR-19-3p/miR-16-5p/miR-30d-5p/	qRT-PCR	sensitivity/specificity/ PPV/NPV/AUROC/cut-off/accuracy
53	Gao	2018	China	N/A	HCC:72(57/15) CH: 50(39/11) hc:50(35/15)	CH/ hc	HCC:51.26 ± 7.31 CH:49.23 ± 8.06 hc:50.37 ± 7.19	plasma	SHNG1	N/A	sensitivity/specificity/ AUC
54	Zhao	2018	China	N/A	HCC:85 (60/25) CHB:50	CHB	HCC:53.2 ± 9.3	Serum	microRNA-143/145	RT-qPCR	sensitivity/specificity/ AUC
55	Li	2014	China	N/A	HCC:31(26/5) CH:31(26/5)	CH	HCC:49 ± 11 CH:49 ± 10	plasma	microRNA-139	RT-qPCR	sensitivity/specificity/ AUC/Cut-off
56	Xie	2014	China	N/A	CHB:79(54/25) LC:61(43/18) HCC:67(57/10) hc:30(21/9)	CHB/ LC/ hc	CHB:32.38 ± 11.93 LC:51.19 ± 9.57 HCC:51.69 ± 10.43 hc: 37.26 ± 10.79	Serum	microRNA-101	RT-qPCR	sensitivity/specificity/ AUC/Cut-off
57	El-Garem	2014	Egypt	case control study	HCC:30(25/5) LC:30(21/9) CH:30(22/8) hc:10(6/4)	LC/ CH/ hc	HCC:60.27 ± 8.20 LC:55.07 ± 7.35 CH:38.20 ± 8.21 hc:40.89 ± 16.85	Serum	miR-221	RT-qPCR	sensitivity/specificity/ AUC
58	Wahb	2021	Egypt	case control study	HCC:35(30/5) CH:33(23/10) hc:32(27/5)	CH/ hc	HCC:55.2 ± 5.2 CH:52.7 ± 5.3 hc:52.8 ± 5.6	Serum	miRNA 9-3p	RT-qPCR	sensitivity/specificity/ PPV/NPV/AUC/cut-off
59	Youssef	2022	Egypt	cohort study	HCC:70(48/22) hc:25	hc	HCC:62.0 ± 7.6	plasma	microRNA-326/microRNA-424/microRNA-511	qRT-PCR	sensitivity/specificity/ PPV/NPV/AUROC/cut-off/accuracy
60	Nomair	2020	Egypt	N/A	HCC:24(15/9) LC:24(14/10) hc:24(15/9)	LC/ hc	HCC: 56.4 ± 7.9 LC: 55.5 ± 6.5 hc: 54.4 ± 5.3	Serum	miRNA-224	RT-PCR	sensitivity/specificity/ AUC/cut-off/accuracy
61	Moshiri	2018	Italy	case-control study	HCC:24 LC:14	LC	N/A	Serum	miR-101-3p/miR-106b-3p	N/A	sensitivity/specificity/ AUC/accuracy
62	Lou	2022	China	N/A	HCC:61(50/11) LC:20(14/6) hc:20(13/7)	LC/ hc	HCC:55.96 ± 12.34 LC:55.31 ± 12.28 hc:41.65 ± 9.90	Serum	lncrna HOTAIR/BRM/ICR	qRT-PCR	sensitivity/specificity/ AUC/cut-off
63	Farag	2018	Saudi Arabia	case-control study	HCC:145(138/7)) CLC:105(70/35)	CLC	N/A	Serum	GP73mRNA	RT-qPCR	sensitivity/specificity/ PPV/NPV/AUC/accuracy/cut-off
64	Shehab-Eldeen	2019	Egypt	cross-sectional study	HCC: 20(16/4) LC: 20(13/7) hc: 20(10/10)	LC/ hc	HCC: 56.95± 1.73 LC: 56.50± 2.1 hc: 52.35± 1.4	Serum	microRNA-122/microRNA-224	RT-qPCR	sensitivity/specificity/ PPV/NPV/AUC/accuracy/cut-off
65	Aboelwafa	2021	Egypt	N/A	LC:100(79/21) HCC:50(40/10) hc:50(35/15)	LC/ hc	LC: 47.4 ± 9.7 HCC: 50.6 ± 5.2 hc: 47.5 ± 9.3	Serum	miR-23b-3p/miR-331-3p	qRT-PCR	sensitivity/specificity/ PPV/NPV/AUC/cut-off
66	Cimentepe	2021	Turkey	N/A	HCC:35(30/5) CHB:35(16/19) hc:30(17/13)	CHB/ hc	HCC:64.7 CHB:35 hc:38	Serum	miR-21-5p/miR-122a-5p/miR-221-5p/miR-223-5p	qRT-PCR	AUC
67	Farag	2018	Saudi Arabia	case-control study	HCC:145(138/7) LC:105(75/30)	LC	N/A	Serum	Glypican-3mRNA	RT-PCR	sensitivity/specificity/ PPV/NPV/AUC/cut-off
68	Gibriela	2022	Egypt	N/A	HCC:40 CLD:48 LC:39 hc:40	CLD/ LC/ hc	N/A	plasma	miR-650/552-3p/676-3p/512-5p/147b	qRT-PCR	sensitivity/specificity/AUC/cut-off
69	Hussein	2022	Egypt	prospective case control study	LC:25 HCC:25 hc:10	LC/ hc	LC:63.44 ± 9 HCC:61 ± 7.76	Serum	MicroRNA 21	RT-PCR	sensitivity/specificity/ AUC/cut-off
70	Shehab-Eldeen	2023	Egypt	case control study	HCC:50(41/9) LC:50(36/14) hc:50(31/19)	LC/ hc	HCC: 58.9 ± 5.5 LC: 57.2 ± 7 hc: 59.7 ± 4	Serum	MicroRNA-29a/MicroRNA-124	RT-qPCR	sensitivity/specificity/ PPV/NPV/AUC/cut-off
71	Xu	2018	China	N/A	HCC:100(74/26) LC:100(74/26) CH:100(74/26) hc:100(74/26)	LC/ CH/ hc	HCC:54.9 ± 13.1 LC:55.9 ± 12.0 CH:55.6 ± 11.7 hc:54.1 ± 13.8	Serum	microRNA-125b	RT-qPCR	sensitivity/specificity/ AUC/cut-off
72	Zuo	2014	China	N/A	HCC:65(48/17) hc:30(17/13)	hc	Age range: HCC:27-79 hc: 24-80	Serum	miR-125b	RT-qPCR	sensitivity/specificity/ AUC/cut-off
73	Song	2020	China	case control study	HCC:94(63/31) CH:52(31/21	CH	Age range;median;>60;<60(numbers) HCC:32-75;61;51/43 CH:41-74;62;28/24	Serum	lncRNA-PVT1	qRT-PCR	sensitivity/specificity/ AUC/cut-off
74	Lyu	2021	China	N/A	HCC:111(89/22) LC:58(43/15) CHB:50(35/15) hc:54(36/18)	LC/ CHB/ hc	<50;>50(numbers) HCC:31/80 LC:16/42 CHB:17/35 hc:22/32	Plasma	hsa circ 0070396	qRT-PCR	sensitivity/specificity/ AUC
75	Wang	2018	China	N/A	HCC: 50(40/10) LC: 40(25/15) CH: 40(31/9) hc: 50(37/13)	LC/ CH/ hc	HCC: 56.32 ± 9.71 LC: 55.13 ± 11.94 CH: 51.25 ± 8.46 hc: 53.92 ± 8.17	Serum	Exosomal miR-122/miR-148a/miR-1246	RT-qPCR	sensitivity/specificity/ AUC
76	Wang	2021	China	N/A	HCC:56(45/11) CHB:57(38/19) LC:47(35/12)	CHB/ LC	N/A	Serum	hsa_circ_0028861	qRT-PCR	sensitivity/specificity/ AUC
77	Wei	2022	China	case control study	HCC:90 hc:90(50/40)	hc	N/A	Serum	microRNA-370-3p/microRNA-196a-5p		sensitivity/specificity/ AUC
78	Chen	2022	China	N/A	HCC:60 hc:60	hc	N/A	Serum	miR-34a	qRT-PCR	sensitivity/specificity/ AUC
79	Ghosh	2020	India	N/A	LC:25 HCC:38 CH:35	LC/ CH	N/A	Serum	miR-10b-5p/miR-223-3p/miR-221-3p/miR-21-5p	qRT-PCR	sensitivity/specificity/ AUC
80	Xu	2018	China	N/A	HCC:55 CHB:60 hc:60	CHB/ hc	N/A	Serum	lncRNA ENSG00000258332.1/LINC00635	qRT-PCR	sensitivity/specificity/ AUC/cut-off
81	Xu	2017	China	N/A	HCC: 88 LC:67 CHB:68 hc:68	LC/ CHB/ hc	N/A	Serum	hnRNPH1 mRNA	qRT-PCR	sensitivity/specificity/ AUC/cut-off
82	Yang	2022	China	N/A	HCC: 50(30/20) LC: 50(29/21) hc: 50(26/24)	LC/ hc	HCC: 60.8 ± 5.3 LC: 63.0 ± 3.4 hc: 61.5 ± 5.2	exosomes/Plasma	microRNA-26a/microRNA-29c/microRNA199a	qRT-PCR	sensitivity/specificity/ AUC

BLD, benign liver disease; CH, Chronic hepatitis; CHB, chronic hepatitis B; CHC, chronic hepatitis C; CLD, Cholestatic Liver Disease; DN, Dysplasia of nodules; HCC, Hepatocellular carcinoma; hc, healthy control; LC, Liver cirrhosis; NAFLD, non-alcoholic fatty liver disease, N/A, Not Applicable.

### 3.3 Quality assessment

**Figure 2:**
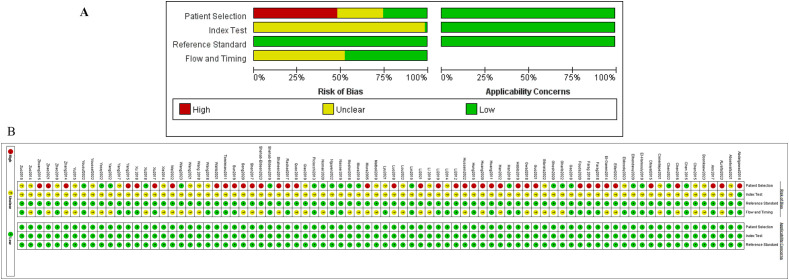
Quality assessment of the studies included using the modifed Qualty Assessment of Diagnostic Accuracy Studies. **(A)** is a summary of publication Bias (the results of the Risk of Bias assessment on the left, Applicability Concerns assessment on the right), and **(B)** is the results of each study assessment.

The quality assessment was performed via the Quality Assessment of Diagnostic Accuracy Studies-2 (QUADAS-2) tool, with results depicted in **Figure 2**. In conclusion, the overall quality of the included studies was moderate to low. Due to 22 studies not specifying whether the patient sample was included consecutively or randomly, the risk of bias in patient selection was rated as unclear. Forty studies were explicitly described as case-control studies, and 39 studies clearly stated non-consecutive inclusion, thus rated as high risk. Since thresholds were not determined before the initial screening, all studies were rated as unclear regarding this aspect. Most studies did not mention whether liquid biopsy was conducted before the gold standard was established, thus rated as unclear. Forty-three studies did not specify whether there was a time interval between the gold standard and liquid biopsy diagnosis, hence rated as unclear. All studies met clinical applicability standards and were rated as low risk. The detailed quality assessment of the included studies is presented in **Figure 2** and **Supplementary Table S4**.

### 3.4 Network meta-analysis

#### 3.4.1 Diagnostic performance of various liquid biopsy-based biomarkers in HCC vs. healthy population

**Figure 3:**
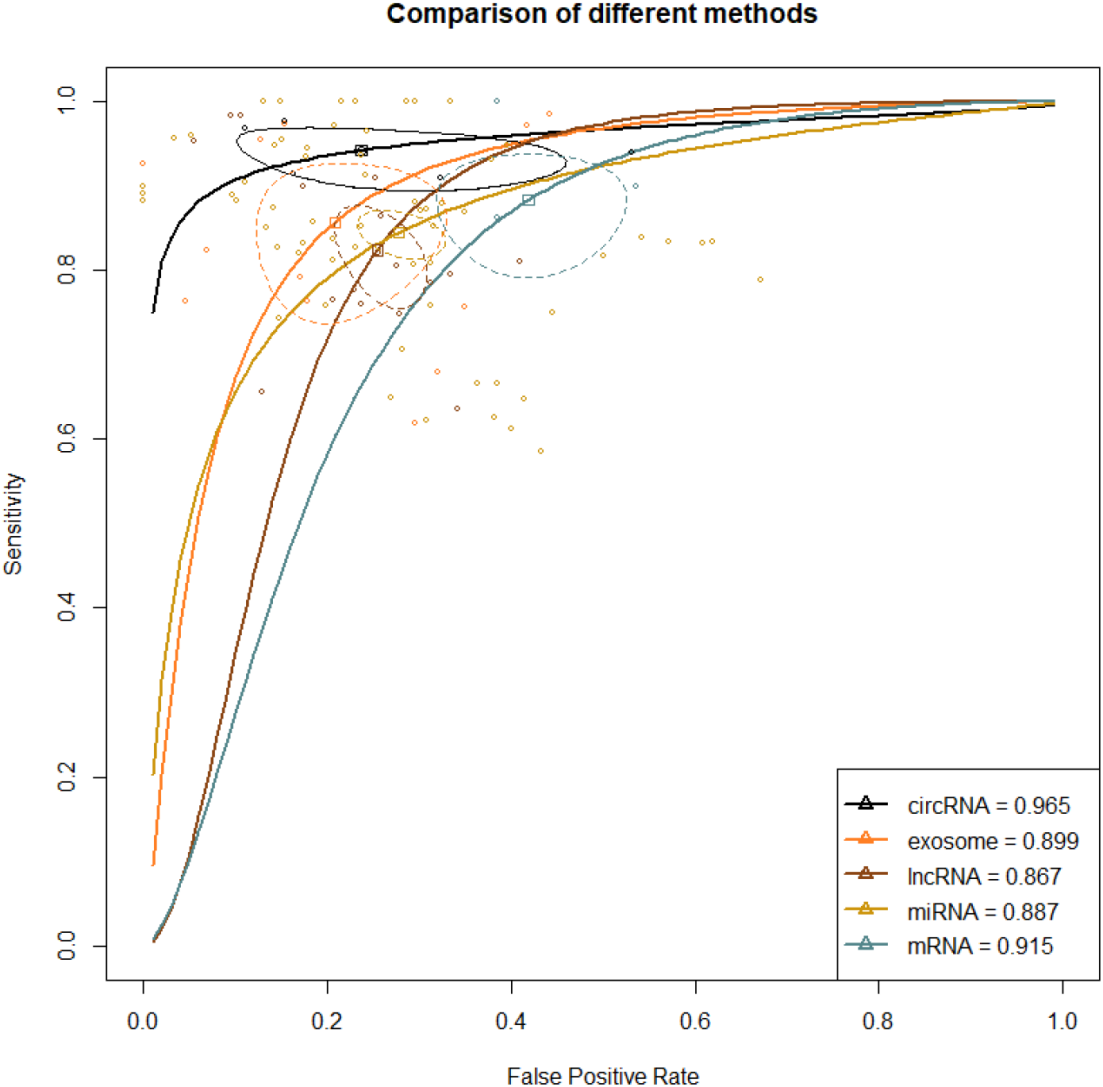
ROC curve of various liquid biopsy-based biomarkers in HCC vs. healthy population. A larger AUC indicates higher sensitivity and specificity, demonstrating that the diagnostic biomarker has higher diagnostic performance. The steepness of the ROC curve reflects the model’s classification performance at different thresholds, particularly its ability to balance the true positive rate and the false positive rate.

A total of 47 studies reported this outcome. Among them, there were 29 studies on miRNA, 8 on lncRNA, 6 on exosome, 3 on circRNA, and 1 on mRNA. By synthesizing direct and indirect evidence through NMA, it was found that the synthesized SEN and SPE of exosome were 0.877 (95% CI [0.784-0.933]) and 0.824 (95% CI [0.718-0.896]), respectively, with an AUC of 0.899 (95% CI [0.834-0.974]) for the ROC curve. The synthesized SEN and SPE of lncRNA were 0.839 (95% CI [0.780-0.884]) and 0.748 (95% CI [0.707-0.786]), respectively, with an AUC of 0.867 (95% CI [0.833-0.904]). The synthesized SEN and SPE of miRNA were 0.871 (95% CI [0.844-0.893]) and 0.752 (95% CI [0.706-0.792]), respectively, with an AUC of 0.887 (95% CI [0.859-0.916]). The synthesized SEN and SPE of circRNA were 0.946 (95% CI [0.915-0.965]) and 0.763 (95% CI [0.609-0.869]), respectively, with an AUC of 0.965 (95% CI [0.944-0.988]). The synthesized SEN and SPE of mRNA were 0.942 (95% CI [0.778-0.987]) and 0.583 (95% CI [0.498-0.664]), respectively, with an AUC of 0.915 (95% CI [0.542-0.933]) (**Table 2**; **Figure 3**).

**Table 2:** Summary of the diagnostic efficacy of different liquid biopsy biomarkers in differentiating HCC from healthy controls and liver disease patients.

Diagnostic techniques	Sensitivity (95%CI)	specificity (95%CI)	Diagnostic odds ratio (95%CI)	Superiority index (95%CI)	Relative sensitivity (95%CI)	Relative specificity (95%CI)
Diagnostic performance based on HCC vs. healthy control						
exosome	0.877 (0.784-0.933)	0.824 (0.718-0.896)	31.504 (12.937-76.719)	2.797 (0.143-9)	1	1
lncRNA	0.839 (0.780-0.884)	0.748 (0.707-0.786)	14.364 (9.278-22.238)	1.774 (0.143-7)	0.978 (0.854-1.133)	1.013 (0.819-1.249)
miRNA	0.871 (0.844-0.893)	0.752 (0.706-0.792)	18.495 (12.843-226.633)	1.88 (0.2-7)	1.005 (0.909-1.145)	0.994 (0.847-1.206)
mRNA	0.942 (0.778-0.987)	0.583 (0.498-0.664)	10.983 (5.280-22.847)	0.538 (0.111-9)	1.024 (0.851-1.208)	0.685 (0.445-0.950)
circRNA	0.946 (0.915-0.965)	0.763 (0.609-0.869)	55.74 (18.211-170.604)	3.55 (0.143-3)	1.057 (0.906-1.218)	0.931 (0.637-1.228)
Diagnostic performance based on HCC vs. liver disease						
exosome	0.785 (0.751-0.816)	0.722 (0.675-0.764)	9.511 (7.627-11.860)	0.593 (0.111-2.333)	1	1
lncRNA	0.871 (0.806-0.917)	0.622 (0.541-0.697)	10.477 (6.793-16.161)	0.621 (0.111-2.333)	1.086 (0.948-1.236)	0.89 (0.674-1.152)
miRNA	0.797 (0.767-0.824)	0.759 (0.725-0.791)	11.71 (9.255-14.817)	0.975 (0.143-2.333)	1.019 (0.921-1.158)	1.062 (0.902-1.277)
mRNA	0.949 (0.932-0.962)	0.935 (0.578-0.993)	139.514 (26.334-739.120)	10.621 (7-11)	1.215 (1.095-1.381)	1.225 (1.018-1.490)
circRNA	0.812 (0.684-0.896)	0.737 (0.686-0.783)	11.386 (5.002-25.920)	0.945 (0.091-5)	1.005 (0.760-1.217)	0.991 (0.628-1.329)
cfDNA	0.792 (0.719-0.850)	0.739 (0.522-0.880)	10.381 (4.535-23.765)	1.028 (0.091-5.05)	1.001 (0.771-0.208)	1.02 (0.709-1.350)

Direct and indirect evidence was synthesized through NMA. In addition, the relative superiority index was introduced to comprehensively assess SEN and SPE. Among them, circRNA ranked the highest in overall diagnosis (superiority index: 3.550 (95% CI [0.143-3]); DOR: 55.740 (95% CI [18.211-170.604])), followed by exosome (superiority index: 2.797 (95% CI [0.143-9]); DOR: 31.504 (95% CI [12.937-76.719])), miRNA (superiority index: 1.880 (95% CI [0.2-7]); DOR: 18.495 (95% CI [12.843-226.633])), lncRNA (superiority index: 1.776 (95% CI [0.143-7]); DOR: 14.364 (95% CI [9.278-22.238])), and mRNA (superiority index: 0.538 (95% CI [0.111-9]); DOR: 10.983 (95% CI [5.280-22.847])) (**Table 2**; **Figure 3**).

#### 3.4.2 Diagnostic performance of various liquid biopsy-based biomarkers in HCC vs. liver disease

**Figure 4:**
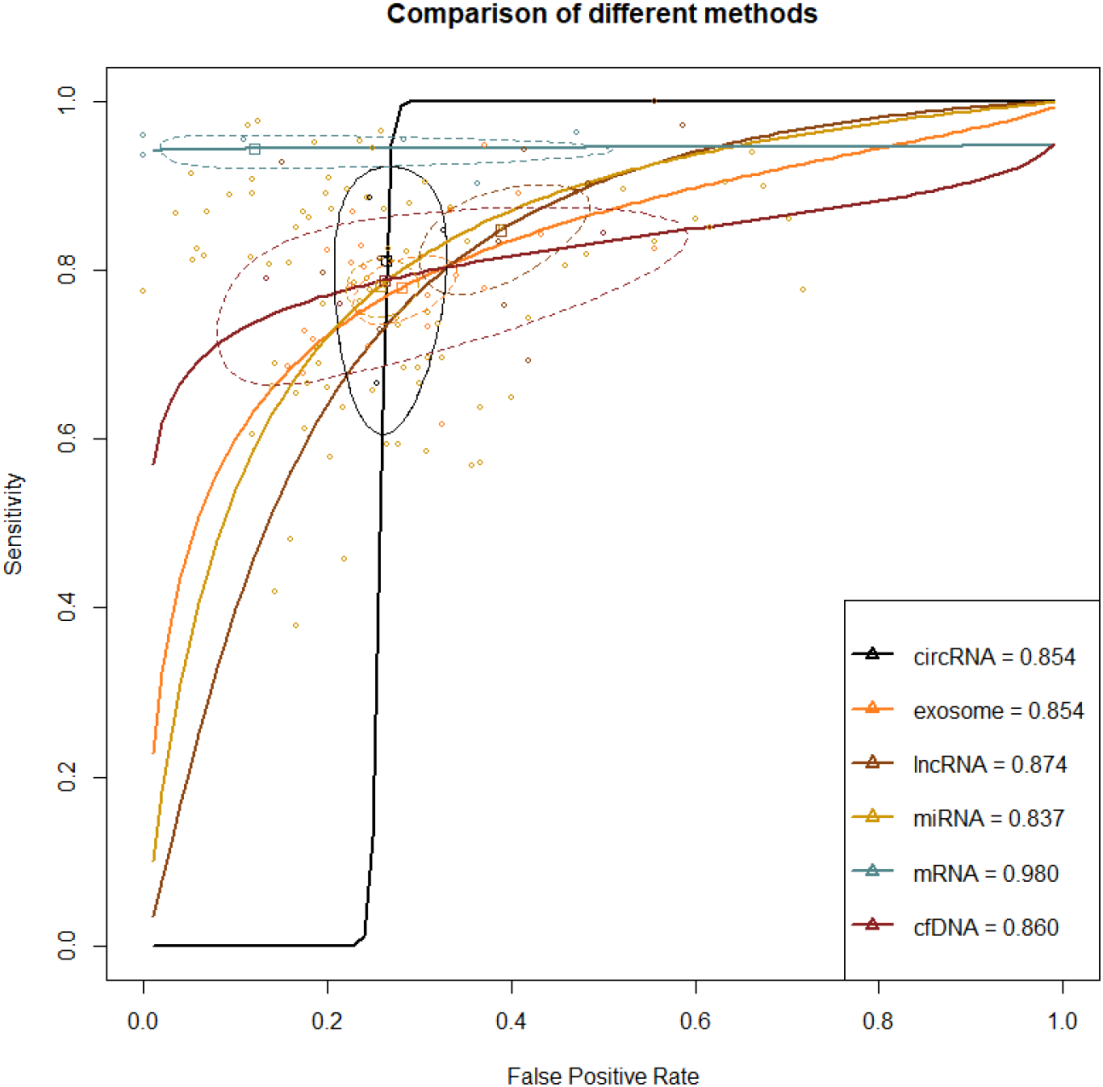
ROC curve of various liquid biopsy-based biomarkers in HCC vs. Liver disease population A larger AUC indicates higher sensitivity and specificity, demonstrating that the diagnostic biomarker has higher diagnostic performance. The steepness of the ROC curve reflects the model’s classification performance at different thresholds, particularly its ability to balance the true positive rate and the false positive rate.

A total of 67 studies reported this outcome. Among them, there were 41 studies on miRNA, 8 on exosome, 7 on lncRNA, 6 on mRNA, 2 on circRNA, and 3 on cfDNA. The synthesized SEN and SPE of exosome were 0.785 (95% CI [0.751-0.816]) and 0.722 (95% CI [0.675-0.764]), respectively, with an AUC of 0.854 (95% CI [0.835-0.874]). The synthesized SEN and SPE of lncRNA were 0.871 (95% CI [0.806-0.917]) and 0.622 (95% CI [0.541-0.697]), respectively, with an AUC of 0.874(95% CI [0.838-0.914]). The synthesized SEN and SPE of miRNA were 0.797 (95% CI [0.767-0.824]) and 0.759 (95% CI [0.725-0.791]), respectively, with an AUC of 0.837(95% CI [0.817-0.858]). The synthesized SEN and SPE of circRNA were 0.812 (95% CI [0.684-0.896]) and 0.737 (95% CI [0.686-0.783]), respectively, with an AUC of 0.854 (95% CI [0.768-0.960]). The synthesized SEN and SPE of mRNA were 0.949 (95% CI [0.932-0.962]) and 0.935 (95% CI [0.578-0.993]), respectively, with an AUC of 0.980(95% CI [0.970-0.990]). The synthesized SEN and SPE of cfDNA were 0.792 (95% CI [0.719-0.850]) and 0.739 (95% CI [0.522-0.880]), respectively, with an AUC of 0.860(95% CI [0.773-0.968]) (**Table 2**; **Figure 4**).

Direct and indirect evidence was synthesized through NMA. In addition, the relative superiority index was introduced to comprehensively assess SEN and SPE. Among them, mRNA ranked highest in overall diagnosis (superiority index: 10.621 (95% CI [7-11]); DOR: 139.514 (95% CI [26.334-739.120])). The others were ranked by relative superiority index from high to low as follows: cfDNA (superiority index: 1.028 (95% CI [0.091-5.05]); DOR: 10.381 (95% CI [4.535-23.765])), miRNA (superiority index: 0.975 (95% CI [0.143-2.333]); DOR: 11.710 (95% CI [9.255-14.817])), circRNA (superiority index: 0.945 (95% CI [0.091-5]); DOR: 11.386 (95% CI [5.002-25.920])), lncRNA (superiority index: 0.621 (95% CI [0.111-2.333]); DOR: 10.477 (95% CI [6.793-16.161])), and exosome (superiority index: 0.593 (95% CI [0.111-2.333]); DOR: 9.511 (95% CI [7.627-11.860])) (**Table 2**; **Figure 4**).

### 3.5 Subgroup analysis

#### 3.5.1 Diagnostic performance of biomarkers in HCC vs. healthy population (based on optimal circRNA)

A total of 3 studies reported these results. The synthesized SEN and SPE for hsa_circ_00156 were 0.940 (95% CI [0.874-0.978]) and 0.469 (95% CI [0.343-0.598]), respectively; for hsa_circ_000224, the synthesized SEN and SPE were 0.976 (95% CI [0.931-0.995]) and 0.846 (95% CI [0.695-0.941]), respectively; for hsa_circ_000520, the synthesized SEN and SPE were 0.969 (95% CI [0.922-0.991]) and 0.889 (95% CI [0.739-0.969]), respectively; for circSMARCA5, the synthesized SEN and SPE were 0.914 (95% CI [0.851-0.956]) and 0.836 (95% CI [0.754-0.900]), respectively; for hsa_circ_0003998, the synthesized SEN and SPE were 0.909 (95% CI [0.829-0.960]) and 0.677 (95% CI [0.547-0.791]), respectively (**Table 3**).

**Table 3:** Subgroup analysis of diagnostic efficacy based on circRNA in differentiating HCC from healthy controls and patients with liver disease.

Diagnostic techniques	Sensitivity (95%CI)	specificity (95%CI)	Diagnostic odds ratio (95%CI)	Superiority index (95%CI)	Relative sensitivity (95%CI)	Relative specificity (95%CI)
Diagnostic performance based on HCC vs. healthy control						
hsa_circ_00156	0.940 (0.874-0.978)	0.469 (0.343-0.598)	13.824 (5.291-36.113)	0.991 (0.111-5)	1.000	1.000
hsa_circ_000224	0.976 (0.931-0.995)	0.846 (0.695-0.941)	223.667 (53.085-942.399)	3.091 (0.143-9)	1.182 (0.479-2.402)	1.834 (0.450-6.145)
hsa_circ _000520	0.969 (0.922-0.991)	0.889 (0.739-0.969)	248 (58.796-1046.062)	2.983 (0.143-9)	1.423 (0.489-2.358)	1.924 (0.396-6.630)
circSMARCA5	0.914 (0.851-0.956)	0.836 (0.754-0.900)	54.364 (24.470-120.779)	2.388 (0.111-9)	1.15 (0.321-2.972)	1.967 (0.322-7.619)
hsa_circ_0003998	0.909 (0.829-0.960)	0.677 (0.547-0.791)	21 (8.530-51.702)	1.887 (0.111-9)	1.146 (0.302-2.961)	1.74 (0.262-6.507)
Diagnostic performance based on HCC vs. liver disease						
circSMARCA5	0.794 (0.597-0.909)	0.750 (0.694-0.799)	11.587 (2.945-45.584)	1.322 (0.333-3)	1.000	1.000
hsa_circ_0003998	0.847 (0.760-0.912)	0.673 (0.529-0.797)	11.392 (5.125-25.324)	1.336 (0.333-3)	1.41 (0.277-4.434)	1.215 (0.206-4.084)

Direct and indirect evidence was synthesized through NMA. In addition, the relative superiority index was introduced to comprehensively assess SEN and SPE. Among them, hsa_circ_000224 ranked highest in overall diagnostics (superiority index: 3.091 (95% CI [0.143-9]); DOR: 223.667 (95% CI [53.085-942.399])). The rest were ranked in descending order of their relative superiority index as follows: hsa_circ_000520 (superiority index: 2.983 (95% CI [0.143-9]); DOR: 248.000 (95% CI [58.796-1046.062])), circSMARCA (superiority index: 2.388 (95% CI [0.111-9]); DOR: 54.364 (95% CI [24.470-120.779])), hsa_circ_0003998 (superiority index: 1.887 (95% CI [0.111-9]); DOR: 21.000 (95% CI [8.530-51.702])), and hsa_circ_00156 (superiority index: 0.991 (95% CI [0.111-5]); DOR: 13.824 (95% CI [5.291-36.113])) (**Table 3**).

#### 3.5.2 Diagnostic performance of biomarkers in HCC vs. liver disease (based on optimal circRNA)

A total of 2 studies reported this outcome. The synthesized SEN and SPE for circSMARCA5 were 0.794 (95% CI [0.597-0.909]) and 0.750 (95% CI [0.694-0.799]), respectively; for hsa_circ_0003998, the synthesized SEN and SPE were 0.847 (95% CI [0.760-0.912]) and 0.673 (95% CI [0.529-0.797]), respectively (**Table 3**).

Direct and indirect evidence was synthesized through NMA. In addition, the relative superiority index was introduced to comprehensively assess SEN and SPE. Among them, hsa_circ_0003998 (superiority index: 1.336 (95% CI [0.333-3]); DOR: 11.392 (95% CI [5.125-25.324])) was superior to circSMARCA5 (Superiority index: 1.322 (95% CI [0.333-3]); DOR: 11.587 (95% CI [2.945-45.584])) (**Table 3**).

#### 3.5.3 Diagnostic performance of biomarkers in HCC vs. healthy population (based on optimal mRNA)

A total of 3 studies reported this outcome. The synthesized SEN and SPE for KIAA0101 mRNA were 1.000 (95% CI [0.932-1.000]) and 0.615 (95% CI [0.486-0.733]), respectively; for RAB11A mRNA, the synthesized SEN and SPE were 0.900 (95% CI [0.782-0.967]) and 0.464 (95% CI [0.275-0.661]), respectively; for SOGA mRNA, the synthesized SEN and SPE were 0.862 (95% CI [0.746-0.939]) and 0.615 (95% CI [0.486-0.766]), respectively (**Table 4**).

**Table 4:** Subgroup analysis of diagnostic efficacy based on mRNA in differentiating HCC from healthy controls and patients with liver disease.

Diagnostic techniques	Sensitivity (95%CI)	specificity (95%CI)	Diagnostic odds ratio (95%CI)	Superiority index (95%CI)	Relative sensitivity (95%CI)	Relative specificity (95%CI)
Diagnostic performance based on HCC vs. healthy control						
RAB11A mRNA	0.9 (0.782-0.967)	0.464 (0.275-0.661)	7.800 (2.384-25.522)	1.197 (0.2-5)	1.000	1.000
SOGA mRNA	0.862 (0.746-0.939)	0.615 (0.446-0.766)	10.000 (3.729-26.818)	1.324 (0.2-5)	1.146 (0.240-3.217)	1.731 (0.184-7.364)
KIAA0101 mRNA	1 (0.932-1.000)	0.615 (0.486-0.733)	166.765 (9.854-2822.254)	2.434 (0.2-5)	1.52 (0.528-4.191)	1.641 (0.201-6.776)
Diagnostic performance based on HCC vs. liver disease						
KIAA0101 mRNA	0.963 (0.873;0.995)	0.528 (0.386-0.667)	29.12 (6.422-132.052)	0.965 (0.143-5)	1.000	1.000
hTERT mRNA	0.949 (0.925-0.965)	0.782 (0.628-0.883)	56.018 (15.537-201.973)	0.957 (0.143-3.05)	1.053 (0.681-1.874)	1.914 (0.481-6.484)
GP-73 mRNA	0.935 (0.885-0.969)	1.000 (0.962-1.000)	2646.714 (153.288-45698.827)	2.443 (0.2-7)	0.996 (0.457-1.849)	2.629 (0.726-9.360)
GPC-3 mRNA	0.960 (0.916-0.985)	1.000 (0.963-1.000)	4454.538 (248.132-79969.267)	3.167 (0.2-7)	1.036 (0.468-1.895)	2.648 (0.776-8.858)

Direct and indirect evidence was synthesized through NMA. In addition, the relative superiority index was introduced to comprehensively assess SEN and SPE. Among them, KIAA0101 mRNA ranked highest in overall diagnostics (superiority index: 2.434 (95% CI [0.2-5]); DOR: 166.765 (95% CI [9.854-2822.254])). The rest were ranked in descending order of their relative superiority index as follows: SOGA mRNA (superiority index: 1.324 (95% CI [0.2-5]); DOR: 10.000 (95% CI [3.729-26.818])) and RAB11A mRNA (superiority index: 1.197 (95% CI [0.2-5]); DOR: 7.800 (95% CI [2.384-25.522])) (**Table 4**).

#### 3.5.4 Diagnostic performance of biomarkers in HCC vs. liver disease (based on optimal mRNA)

A total of 3 studies reported these results. The synthesized SEN and SPE for KIAA0101 mRNA were 0.963 (95% CI [0.873-0.995]) and 0.528 (95% CI [0.386-0.667]), respectively; for hTERT mRNA, the synthesized SEN and SPE were 0.949 (95% CI [0.925-0.965]) and 0.782 (95% CI [0.628-0.883]), respectively; for GP-73 mRNA, the synthesized SEN and SPE were 0.935 (95% CI [0.885-0.969]) and 1.000 (95% CI [0.962-1.000]), respectively; for GPC-3 mRNA, the synthesized SEN and SPE were 0.960 (95% CI [0.916-0.985]) and 1.000 (95% CI [0.963-1.000]), respectively (**Table 4**).

Direct and indirect evidence was synthesized through NMA. In addition, the relative superiority index was introduced to comprehensively assess SEN and SPE. Among them, GPC-3 mRNA ranked highest in overall diagnostics (superiority index: 3.167 (95% CI [0.2-7]); DOR: 4454.538 (95% CI [248.132-79969.267])). The rest were ranked in descending order of their relative superiority index as follows: GP-73 mRNA (superiority index: 2.443 (95% CI [0.2-7]); DOR: 2646.7141 (95% CI [153.288-45698.827])), KIAA0101 mRNA (superiority index: 0.965 (95% CI [0.143-5]); DOR: 29.120 (95% CI [6.422-132.052])), and hTERT mRNA (superiority index: 0.957 (95% CI [0.143-3.05]); DOR: 56.018 (95% CI [15.537-201.973])) (**Table 4**).

Additionally, subgroup analyses for the diagnostic performance of second-ranking (HCC vs healthy: exosome; HCC vs liver disease: cfDNA) and third-ranking (HCC vs healthy: miRNA; HCC vs liver disease: miRNA) biomarkers are detailed in **Supplementary Figure S1**.

## 4 Discussion

Reliable biomarkers are crucial for early HCC diagnosis. However, the diagnostic performance of current biomarkers remains unsatisfactory (24). Moreover, histological examination is limited by its invasiveness, while imaging techniques like CT scans and MRI have restrictions in detecting small tumors (25). Therefore, the lack of effective early diagnosis methods for hepatocellular carcinoma results in most patients being diagnosed at an advanced stage, thus missing the best opportunity for treatment. Among non-invasive diagnostic methods, liquid biopsy has attracted considerable attention and has paved the way for novel avenues in cancer detection (16, 26). Liquid biopsy holds the potential to improve the accuracy of early HCC diagnosis and is currently regarded as one of the state-of-the-art diagnostic techniques available. Major biomarkers commonly utilized in liquid biopsy include miRNA, circRNA, mRNA, lncRNA, cfDNA, and exosomes. Given the absence of direct or indirect comparisons of diagnostic performance among these biomarkers in existing studies, the results obtained from the NMA in this study can offer valuable insights into current clinical practice.

This study represents the first comprehensive comparison of all liquid biopsy-based diagnostic biomarkers for HCC. Through systematic review and the NMA, several significant findings have emerged. Firstly, based on the superiority index comparison, circRNA emerged as the most effective biomarker for distinguishing HCC from healthy individuals. circRNAs, a recently discovered type of non-coding RNA, possess a covalently closed continuous structure without 5’ or 3’ ends. They are renowned for their abundance and stability, and often exhibit tissue- or developmental stage-specific expression. circRNAs are widely present in various cancer tissues, as well as in saliva, blood, and exosomes. Research has indicated that circRNAs can regulate tumor progression by serving as miRNA sponges or modulating the expression of parental genes (27–29). In recent years, mounting evidence has demonstrated the common occurrence of abnormal circRNA expression in various malignancies such as gastric cancer (30), breast cancer (31), lung cancer (32), and colorectal cancer (33).

This study conducted further subgroup analyses on the top-ranked diagnostic biomarkers. The subgroup analysis of circRNA revealed that hsa_circ_000224 and hsa_circ_0003998 are the most effective diagnostic biomarkers for distinguishing HCC from healthy individuals, as well as patients with liver disease, respectively. hsa_circ_000224 is a circRNA transcript of the c17orf107 gene, a protein-coding gene located on chromosome 17. The sequence of this gene is highly conserved across mammalian species. A study by Matboli et al. demonstrated a strong association between hsa_circ_000224 and HCC using the cirBase and Circ2Traits databases, and subsequent clinical trials confirmed its potential as a novel diagnostic biomarker for HCC (34). hsa_circ_0003998 is a spliced sequence consisting of 304 nucleotides in length, with its gene located at chr20: 47570092-47580435. It originates from exons 6 and 7 of the ADP-ribosylation factor guanine nucleotide exchange factor 2 (ARFGEF2) gene. The expression of hsa_circ_0003998 is notably higher in HCC tissues with portal vein invasion compared to those without it. HCC is prone to invasion and metastasis to the portal vein, which contributes to its high recurrence rate and poor prognosis. Qiao et al. (35) observed a significantly higher expression of hsa_circ_0003998 in HCC tissues compared to adjacent non-cancerous tissues (*P<* 0.001). Additionally, its expression level was significantly elevated in HCC cell lines relative to healthy human liver cell lines (*P*< 0.001).

Sensitivity and specificity analysis revealed that among biomarkers for distinguishing HCC from healthy individuals, circRNA exhibited the highest sensitivity, with hsa_circ_000224 being the most sensitive biomarker. The highest specificity was observed in exosomes, particularly in exosomal miR-122. miR-122 is the most abundant microRNA in the liver, accounting for 70%. As a tumor suppressor gene, miR-122 is downregulated in HCC cell lines and tumor tissues (36), leading to the activation of the Wnt/β-catenin pathway, thereby promoting the formation and progression of HCC. Luo et al. discovered that exosomal delivery of miR-122 from adipose tissue-derived mesenchymal stem cells (AMSCs) could enhance the chemosensitivity of HCC (37). Recent studies have demonstrated that exosomal miR-122 showed excellent diagnostic and predictive value in HCC patients with various coexisting diseases (38, 39). Hence, conducting prospective, large-scale studies on HCC patients with diverse comorbidities is essential for future research.

Additionally, through comparative superiority index analysis, it is determined that mRNA serves as not only the optimal diagnostic biomarker in distinguishing HCC from liver disease patients but also demonstrates the highest sensitivity and specificity. mRNA, a single-stranded ribonucleic acid, constitutes only 2% to 5% of the total cellular RNA but exists in diverse functional forms. Investigations have discovered that hepatitis B virus mRNA may directly contribute to the progression from hepatitis B to liver cancer. Given the significant role of mRNA in liver cancer development, it stands as a potential therapeutic target for this disease. Consequently, comprehensive investigations into the mechanisms of mRNA in liver cancer can offer novel insights and approaches for the prevention, diagnosis, and treatment of this malignancy.

Subgroup analysis of mRNA revealed that by comparing the superiority index, KIAA0101 mRNA and GPC-3 mRNA emerged as the optimal diagnostic biomarkers for distinguishing HCC from healthy individuals and liver disease patients, respectively. Through sensitivity and specificity analysis, it was discovered that KIAA0101 mRNA exhibits the highest sensitivity in distinguishing HCC from liver disease patients, whereas GPC-3 mRNA and GP-73 mRNA demonstrated the highest specificity. KIAA0101 is a 15-kDa protein harboring a conserved proliferating cell nuclear antigen (PCNA) binding motif (40, 41). DNA repair regulation (42), cell cycle progression, apoptosis, and cell proliferation (43) are all regulated by this protein. Past research has affirmed the involvement of KIAA0101 in the invasion and metastasis processes of HCC. Yuan et al. (44) detected the overexpression of KIAA0101 mRNA and KIAA0101 protein (61%) in HCC patients. However, certain studies (45) have reported a decrease in KIAA0101 protein levels in HCC, which may be attributed to diverse underlying etiologies. Tantiwetrueangd et al. (46) utilized real-time quantitative PCR (qRT-PCR) to assess the expression levels of KIAA0101/PCLAF mRNA in 40 pairs of HCC tissues and matched non-cancerous tissues, identifying a significant correlation with p53 and Ki-67. Hence, KIAA0101 mRNA, in conjunction with p53 and Ki-67 proteins, holds promise as potential biomarkers for HCC. The above-mentioned studies underscored the significance of KIAA0101 mRNA in the diagnosis and treatment of HCC. Future cross-sectional, case-control, or randomized controlled studies could further delve into novel diagnostic and therapeutic strategies for HCC. Glypican-3 (GPC-3) is a heparan sulfate proteoglycan cell surface protein that binds to the cell membrane and interacts with various growth factors. It can facilitate the development of liver cancer by influencing migration, proliferation, and regulation of cell survival across different tissues (47, 48). In 1997, Hsu et al. initially reported increased expression of GPC-3 in primary liver cancer (47). Also, recent immunohistochemical studies have identified elevated GPC-3 expression levels in HCC patients (49). Research has demonstrated that GPC-3 can upregulate the expression of c-Myc, thereby contributing to the onset and progression of HCC (50, 51). It has been confirmed that GPC-3 can be used to detect AFP-negative HCC cases, and GPC-3 mRNA is remarkably upregulated in HCC compared to normal and benign liver samples (52, 53). These studies all underscored the crucial role of GPC-3 mRNA in the diagnosis and treatment of HCC. Previous studies have indicated that GP-73 mRNA surpasses AFP in the early detection of HCC, exhibiting high sensitivity and specificity (54, 55). Our study further confirmed that GP-73 mRNA displays high specificity in diagnosing HCC, consistent with multiple studies indicating that GP-73 mRNA is notably upregulated in HCC compared to healthy and benign liver samples (56–59).

Based on the types of liquid biopsy, the included studies in our research ranked from highest to lowest are on miRNA, lncRNA, exosome, mRNA, circRNA, cfDNA, and circDNA. While miRNA exhibited an overall inferior diagnostic performance compared to circRNA and mRNA, it had the highest number of publications in diagnosing HCC. This was evident during our literature search and inclusion process. MicroRNAs (miRNAs) are a subset of non-coding RNAs, typically consisting of approximately 22 nucleotides (60). They have garnered increasing attention from scientists due to their potential application as biomarkers for the identification and management of cancer (6, 15)As a class of non-coding RNAs, miRNAs are essential in processes such as cell growth, differentiation, and apoptosis by regulating the expression of target genes (61). Since aberrant miRNA expression in HCC is strongly linked to the onset and progression of malignancies (62), miRNA is a crucial molecule in HCC study. miRNAs exhibit high stability in blood, are not easily degraded, and possess high sensitivity and accuracy, which makes blood-based miRNA detection feasible (63). Furthermore, the non-invasive diagnosis of HCC is made easier by the non-invasive sample techniques (64). Advances in high-throughput sequencing technology and innovative detection methods have made it possible to more thoroughly and precisely detect and evaluate the expression profiles of miRNAs (65). The sensitivity and specificity of these techniques have also been steadily increasing, offering technical support for studies on miRNAs in the diagnosis of HCC.

Prior research has demonstrated that miRNA can function as oncogenes or tumor suppressor genes by inhibiting protein-coding genes implicated in cancer onset and progression (66, 67). Subgroup analysis in this study revealed that miR-148a and miR-9-3p emerged as the optimal biomarkers for diagnosing HCC, distinguishing HCC from healthy individuals and liver disease patients, respectively. This finding underscores the broad applicability and practical utility of miRNA in diagnosing HCC, highlighting its significance. There are, however, very few studies on the application of cfDNA/circDNA for HCC diagnosis in this research. Nonetheless, as most research on cfDNA in HCC focuses on predictive or diagnostic models, cfDNA remains highly promising for HCC diagnosis (68, 69). A recent study reported a model that integrated four cfDNA features, which enabled early detection of HCC in patients with cirrhosis. The model achieved a sensitivity of 95% and a specificity of 97% (70). However, the current standardization of cfDNA/circDNA testing is inadequate, leading to potential variations in test results between different laboratories. Additionally, limited awareness and understanding of cfDNA/circDNA testing among physicians and patients may restrict its application. Therefore, improving the standardization of testing and enhancing the awareness and acceptance of cfDNA/circDNA testing among physicians and patients are essential for promoting its widespread use.

The strengths of our study include a comprehensive search of diagnostic biomarkers for HCC, the use of strict inclusion and exclusion criteria for detailed data extraction, and the first application of the relative superiority index to compare different diagnostic biomarkers. Furthermore, we conducted additional analyses of the optimal category of biomarkers to identify more precise subtypes.

However, limitations are inevitable. First, most of the included studies employed a case-control design with a relatively small sample size, which may affect the reliability and generalizability of the results. Although the variance analysis model used in the NMA can potentially mitigate the inherent bias risks of observational studies, it cannot completely eliminate systematic errors arising from study design. Future research will benefit from more direct comparisons. Second, since liver diseases encompass a variety of conditions, such as liver cirrhosis, chronic hepatitis, hepatitis B, and hepatitis C, these differing pathological backgrounds may affect the expression and diagnostic performance of biomarkers. The lack of sufficient consideration for this heterogeneity in existing studies may lead to significant variability in results across studies, warranting caution when interpreting our conclusions. Third, although cfDNA is widely regarded as a potential biomarker for liver cancer, the number of existing studies is limited. Additionally, the detection methods and standardized processes for different liquid biopsy biomarkers are not yet fully unified, which may contribute to discrepancies in detection results. Finally, the staging data of patients with HCC in most studies are incomplete, hindering a precise and comprehensive evaluation of the diagnostic performance of these biomarkers. Therefore, future studies could focus on conducting prospective, large-sample, multi-center research, particularly including more studies on the use of cfDNA for diagnosing HCC. Efforts should also be made to standardize the collection, preservation, extraction, and detection methods for liquid biopsy biomarkers to enhance the credibility and comparability of research findings. Additionally, the standardization of staging data for HCC patients should be strengthened to ensure data completeness and accuracy, enabling a more comprehensive evaluation of the diagnostic performance of biomarkers across different stages of HCC.

This study concluded that circRNA and mRNA are the optimal biomarkers for diagnosing HCC, which holds significant clinical value in the early diagnosis of this disease, evaluation of therapeutic efficacy, and dynamic monitoring of tumor progression and recurrence. On one hand, identifying the optimal diagnostic biomarkers can help physicians select the optimal treatment strategy. On the other hand, commonly used diagnostic methods, such as liver biopsy and ultrasound-guided percutaneous liver biopsy, are invasive techniques with associated surgical risks. However, liquid biopsy enables detection through blood tests, offering a non-invasive alternative that reduces medical costs, improves treatment efficiency, and provides high convenience and patient compliance.

## 5 Conclusion

In conclusion, this study identified circDNA and mRNA as the optimal biomarkers for HCC diagnosis. Specifically, hsa_circ_000224, hsa_circ_0003998, KIAA0101 mRNA, and GPC-3 mRNA demonstrated promising potential for clinical utilization. In clinical practice, multiple liquid biopsy-based biomarkers are frequently combined to diagnose HCC. Notwithstanding, this study only compared individual liquid biopsy-based biomarkers. Future comparative studies are needed to determine whether combining multiple markers improves diagnostic performance. Given the limitations of this study, clinicians should interpret the ranked outcomes with caution when diagnosing HCC. Further research is urgently needed to improve the diagnostic performance of biomarkers and validate the diagnostic performance of these indicator technologies.

## Data Availability

The original contributions presented in the study are included in the article/**Supplementary Material**. Further inquiries can be directed to the corresponding author.

## Supplementary material

Supplementary Figure 1 Supplementary analysis.

Supplementary Table 4 Quality evaluation of included studies.
