# Supplementary material for: Diagnosis of hepatocellular carcinoma using liquid biopsy-based biomarkers: a systematic review and network meta-analysis
Source: Front Oncol. 2025 Jan 28;14:1483521. doi: 10.3389/fonc.2024.1483521 (PMC11810725; doi:10.3389/fonc.2024.1483521)
Supplement: Supplementary Figure 1 — Supplementary analysis. [file Image1.pdf]

Figure S1 Supplementary analysis

1.Various liquid biopsy-based biomarkers in HCC vs. healthy population

①Sensitivity analysis

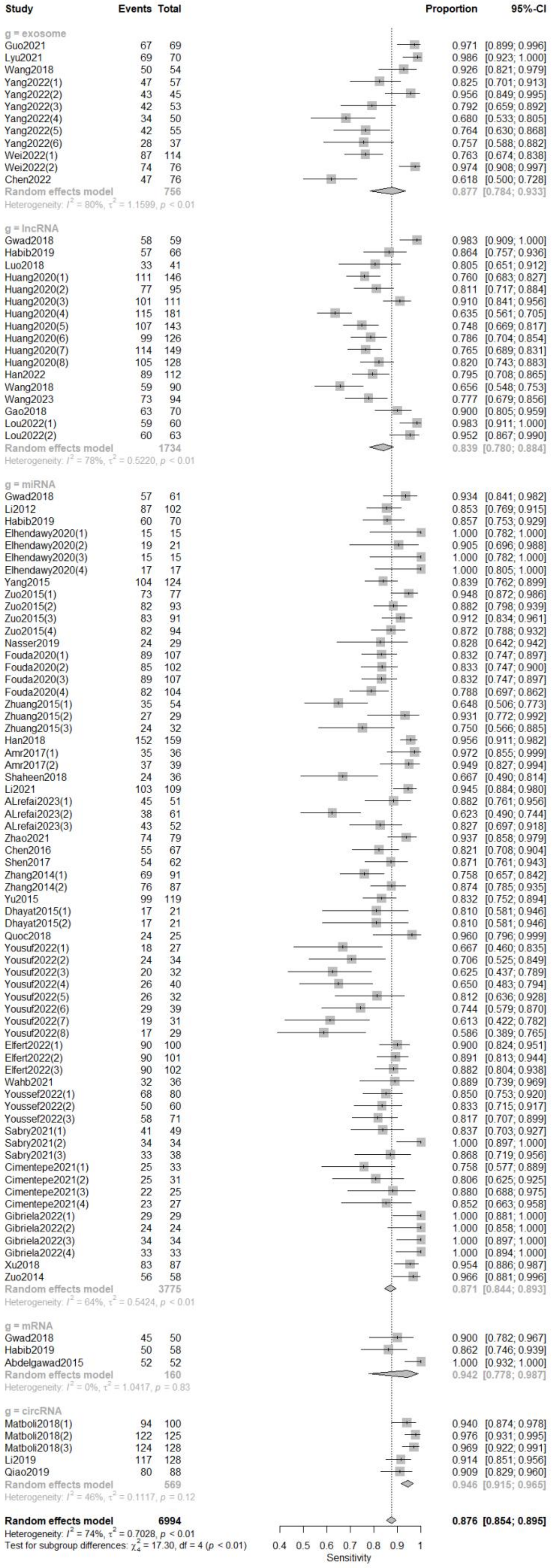

②Specificity analysis

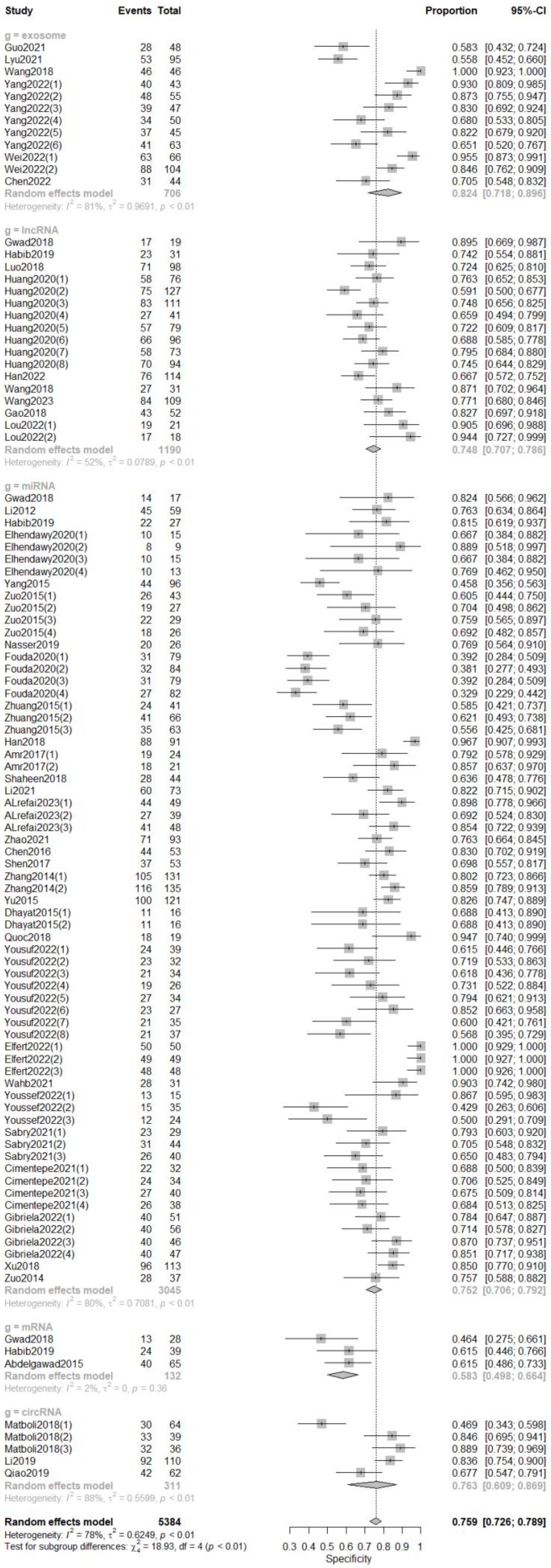

③DOR

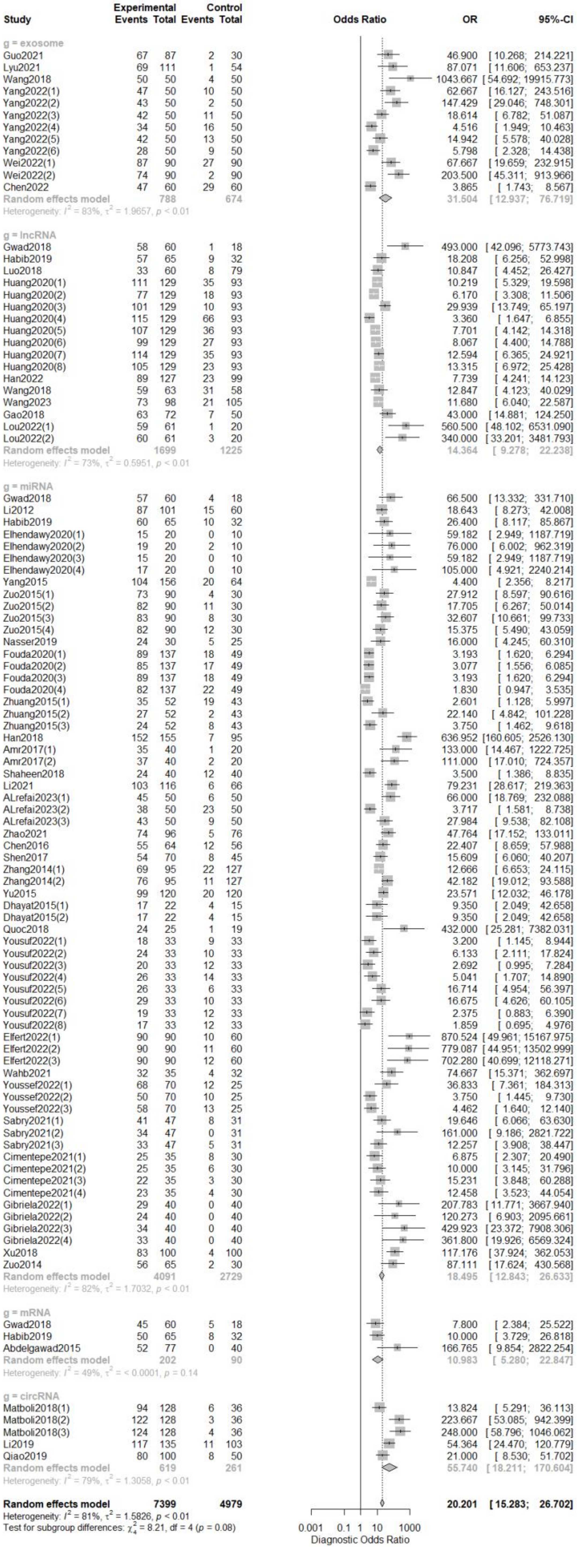

2. Various liquid biopsy-based biomarkers in HCC vs. Liver disease

① Sensitivity analysis

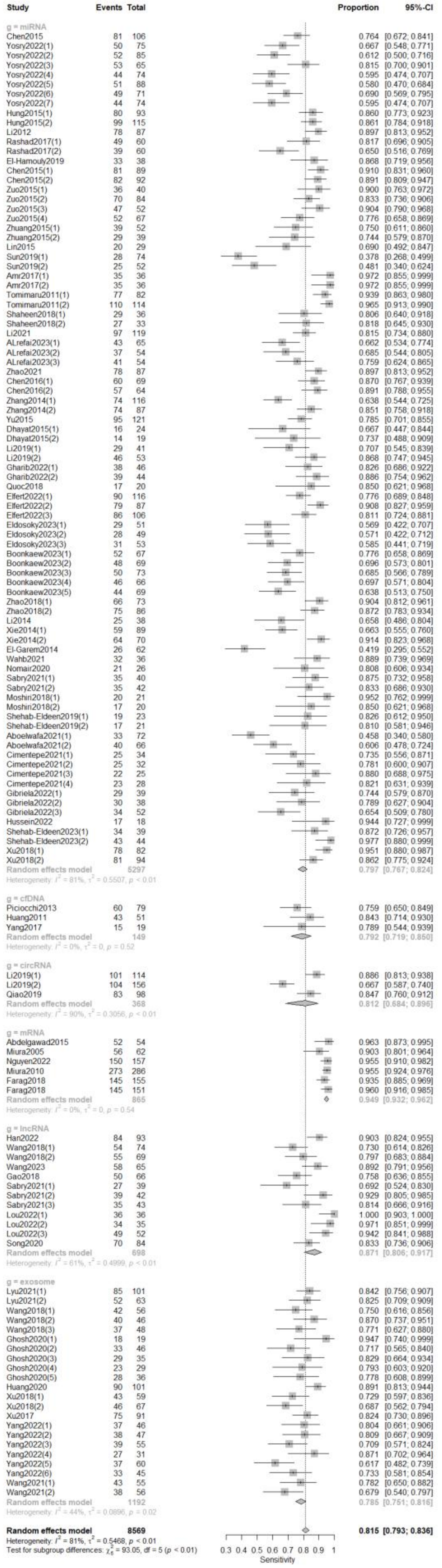

② Specificity analysis

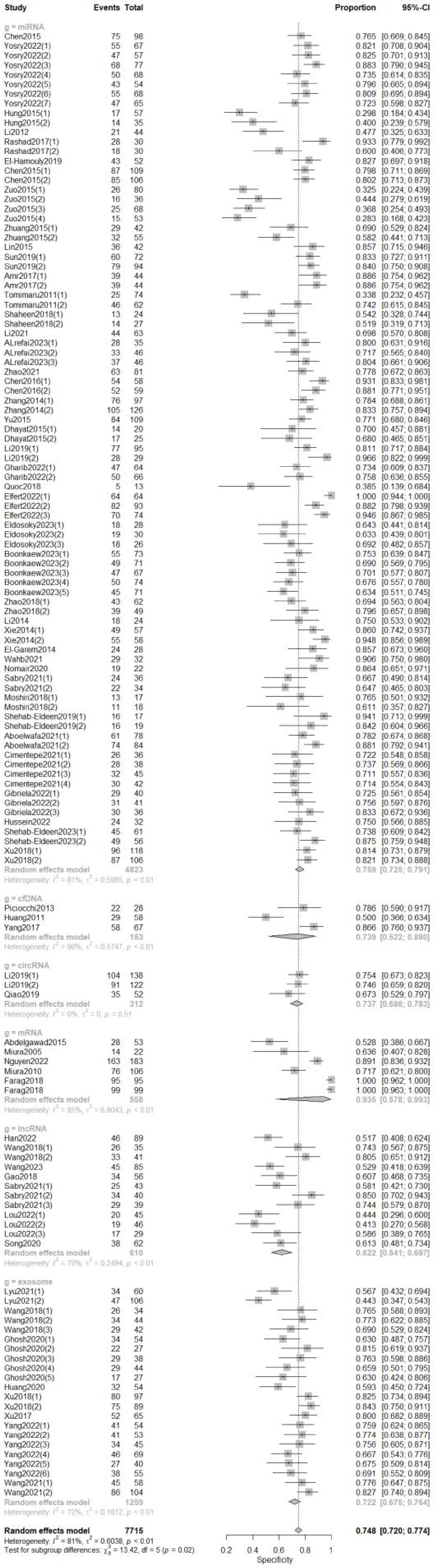
Study Events Total Proportion 95%-CI
g = miRNA
Chen2015 75 98 0.765 [0.669; 0.845]
Yosry2022(1) 55 67 0.821 [0.708; 0.904]
Yosry2022(2) 47 57 0.825 [0.701; 0.913]
Yosry2022(3) 68 77 0.883 [0.790; 0.945]
Yosry2022(4) 50 68 0.735 [0.614; 0.835]
Yosry2022(5) 43 54 0.796 [0.665; 0.894]
Yosry2022(6) 55 68 0.809 [0.695; 0.894]
Yosry2022(7) 47 65 0.723 [0.598; 0.827]
Hung2015(1) 17 57 0.298 [0.184; 0.434]
Hung2015(2) 14 35 0.400 [0.239; 0.579]
Li2012 21 44 0.477 [0.325; 0.633]
Rashad2017(1) 28 30 0.933 [0.779; 0.992]
Rashad2017(2) 18 30 0.600 [0.406; 0.773]
El-Hamouly2019 43 52 0.827 [0.697; 0.918]
Chen2015(1) 87 109 0.798 [0.711; 0.869]
Chen2015(2) 85 106 0.802 [0.713; 0.873]
Zuo2015(1) 26 80 0.325 [0.224; 0.439]
Zuo2015(2) 16 36 0.444 [0.279; 0.619]
Zuo2015(3) 25 68 0.368 [0.254; 0.493]
Zuo2015(4) 15 53 0.283 [0.168; 0.423]
Zhuang2015(1) 29 42 0.690 [0.529; 0.824]
Zhuang2015(2) 32 55 0.582 [0.441; 0.713]
Lin2015 36 42 0.857 [0.715; 0.946]
Sun2019(1) 60 72 0.833 [0.727; 0.911]
Sun2019(2) 79 94 0.840 [0.750; 0.908]
Amr2017(1) 39 44 0.886 [0.754; 0.962]
Amr2017(2) 39 44 0.886 [0.754; 0.962]
Tomimaru2011(1) 25 74 0.338 [0.232; 0.457]
Tomimaru2011(2) 46 62 0.742 [0.615; 0.845]
Shaheen2018(1) 13 24 0.542 [0.328; 0.744]
Shaheen2018(2) 14 27 0.519 [0.319; 0.713]
Li2021 44 63 0.698 [0.570; 0.808]
ALrefai2023(1) 28 35 0.800 [0.631; 0.916]
ALrefai2023(2) 33 46 0.717 [0.565; 0.840]
ALrefai2023(3) 37 46 0.804 [0.661; 0.906]
Zhao2021 63 81 0.778 [0.672; 0.863]
Chen2016(1) 54 58 0.931 [0.833; 0.981]
Chen2016(2) 52 59 0.881 [0.771; 0.951]
Zhang2014(1) 76 97 0.784 [0.688; 0.861]
Zhang2014(2) 105 126 0.833 [0.757; 0.894]
Yu2015 84 109 0.771 [0.680; 0.846]
Dhayat2015(1) 14 20 0.700 [0.457; 0.881]
Dhayat2015(2) 17 25 0.680 [0.465; 0.851]
Li2019(1) 77 95 0.811 [0.717; 0.884]
Li2019(2) 28 29 0.966 [0.822; 0.999]
Gharib2022(1) 47 64 0.734 [0.609; 0.837]
Gharib2022(2) 50 66 0.758 [0.636; 0.855]
Quoc2018 5 13 0.385 [0.139; 0.684]
Elfert2022(1) 64 64 1.000 [0.944; 1.000]
Elfert2022(2) 82 93 0.882 [0.798; 0.939]
Elfert2022(3) 70 74 0.946 [0.867; 0.985]
Eldosoky2023(1) 18 28 0.643 [0.441; 0.814]
Eldosoky2023(2) 19 30 0.633 [0.439; 0.801]
Eldosoky2023(3) 18 26 0.692 [0.482; 0.857]
Boonkaew2023(1) 55 73 0.753 [0.639; 0.847]
Boonkaew2023(2) 49 71 0.690 [0.569; 0.795]
Boonkaew2023(3) 47 67 0.701 [0.577; 0.807]
Boonkaew2023(4) 50 74 0.676 [0.557; 0.780]
Boonkaew2023(5) 45 71 0.634 [0.511; 0.745]
Zhao2018(1) 43 62 0.694 [0.563; 0.804]
Zhao2018(2) 39 49 0.796 [0.657; 0.898]
Li2014 18 24 0.750 [0.533; 0.902]
Xie2014(1) 49 57 0.860 [0.742; 0.937]
Xie2014(2) 55 58 0.948 [0.856; 0.989]
El-Garem2014 24 28 0.857 [0.673; 0.960]
Wahb2021 29 32 0.906 [0.750; 0.980]
Nomair2020 19 22 0.864 [0.651; 0.971]
Sabry2021(1) 24 36 0.667 [0.490; 0.814]
Sabry2021(2) 22 34 0.647 [0.465; 0.803]
Moshiri2018(1) 13 17 0.765 [0.501; 0.932]
Moshiri2018(2) 11 18 0.611 [0.357; 0.827]
Shehab-Eldeen2019(1) 16 17 0.941 [0.713; 0.999]
Shehab-Eldeen2019(2) 16 19 0.842 [0.604; 0.966]
Aboelwafa2021(1) 61 78 0.782 [0.674; 0.868]
Aboelwafa2021(2) 74 84 0.881 [0.792; 0.941]
Cimentepe2021(1) 26 36 0.722 [0.548; 0.858]
Cimentepe2021(2) 28 38 0.737 [0.569; 0.866]
Cimentepe2021(3) 32 45 0.711 [0.557; 0.836]
Cimentepe2021(4) 30 42 0.714 [0.554; 0.843]
Gibriela2022(1) 29 40 0.725 [0.561; 0.854]
Gibriela2022(2) 31 41 0.756 [0.597; 0.876]
Gibriela2022(3) 30 36 0.833 [0.672; 0.936]
Hussein2022 24 32 0.750 [0.566; 0.885]
Shehab-Eldeen2023(1) 45 61 0.738 [0.609; 0.842]
Shehab-Eldeen2023(2) 49 56 0.875 [0.759; 0.948]
Xu2018(1) 96 118 0.814 [0.731; 0.879]
Xu2018(2) 87 106 0.821 [0.734; 0.888]
Random effects model 4823 0.759 [0.725; 0.791]
Heterogeneity: I^2 = 81%, τ^2 = 0.5985, p < 0.01
g = cfDNA
Piciocchi2013 22 28 0.786 [0.590; 0.917]
Huang2011 29 58 0.500 [0.366; 0.634]
Yang2017 58 67 0.866 [0.760; 0.937]
Random effects model 153 0.739 [0.522; 0.880]
Heterogeneity: I^2 = 90%, τ^2 = 0.5747, p < 0.01
g = circRNA
Li2019(1) 104 138 0.754 [0.673; 0.823]
Li2019(2) 91 122 0.746 [0.659; 0.820]
Qiao2019 35 52 0.673 [0.529; 0.797]
Random effects model 312 0.737 [0.686; 0.783]
Heterogeneity: I^2 = 0%, τ^2 = 0, p = 0.51
g = mRNA
Abdelgawad2015 28 53 0.528 [0.386; 0.667]
Miura2005 14 22 0.636 [0.407; 0.828]
Nguyen2022 163 183 0.891 [0.836; 0.932]
Miura2010 76 106 0.717 [0.621; 0.800]
Farag2018 95 95 1.000 [0.962; 1.000]
Farag2018 99 99 1.000 [0.963; 1.000]
Random effects model 558 0.936 [0.578; 0.993]
Heterogeneity: I^2 = 85%, τ^2 = 6.9043, p < 0.01
g = lncRNA
Han2022 46 89 0.517 [0.408; 0.624]
Wang2018(1) 26 35 0.743 [0.567; 0.875]
Wang2018(2) 33 41 0.805 [0.651; 0.912]
Wang2023 45 85 0.529 [0.418; 0.639]
Gao2018 34 56 0.607 [0.468; 0.735]
Sabry2021(1) 25 43 0.581 [0.421; 0.730]
Sabry2021(2) 34 40 0.850 [0.702; 0.943]
Sabry2021(3) 29 39 0.744 [0.579; 0.870]
Lou2022(1) 20 45 0.444 [0.296; 0.600]
Lou2022(2) 19 46 0.413 [0.270; 0.568]
Lou2022(3) 17 29 0.586 [0.389; 0.765]
Song2020 38 62 0.613 [0.481; 0.734]
Random effects model 610 0.622 [0.541; 0.697]
Heterogeneity: I^2 = 70%, τ^2 = 0.2494, p < 0.01
g = exosome
Lyu2021(1) 34 60 0.567 [0.432; 0.694]
Lyu2021(2) 47 106 0.443 [0.347; 0.543]
Wang2018(1) 26 34 0.765 [0.588; 0.893]
Wang2018(2) 34 44 0.773 [0.622; 0.885]
Wang2018(3) 29 42 0.690 [0.529; 0.824]
Ghosh2020(1) 34 54 0.630 [0.487; 0.757]
Ghosh2020(2) 22 27 0.815 [0.619; 0.937]
Ghosh2020(3) 29 38 0.763 [0.598; 0.886]
Ghosh2020(4) 29 44 0.659 [0.501; 0.795]
Ghosh2020(5) 17 27 0.630 [0.424; 0.806]
Huang2020 32 54 0.593 [0.450; 0.724]
Xu2018(1) 80 97 0.825 [0.734; 0.894]
Xu2018(2) 75 89 0.843 [0.750; 0.911]
Xu2017 52 65 0.800 [0.682; 0.889]
Yang2022(1) 41 54 0.759 [0.624; 0.865]
Yang2022(2) 41 53 0.774 [0.638; 0.877]
Yang2022(3) 34 45 0.756 [0.605; 0.871]
Yang2022(4) 46 69 0.667 [0.543; 0.776]
Yang2022(5) 27 40 0.675 [0.509; 0.814]
Yang2022(6) 38 55 0.691 [0.552; 0.809]
Wang2021(1) 45 58 0.776 [0.647; 0.875]
Wang2021(2) 86 104 0.827 [0.740; 0.894]
Random effects model 1259 0.722 [0.675; 0.764]
Heterogeneity: I^2 = 72%, τ^2 = 0.1812, p < 0.01
Random effects model 7715 0.748 [0.720; 0.774]
Heterogeneity: I^2 = 81%, τ^2 = 0.6038, p < 0.01
Test for subgroup differences: χ^2_5 = 13.42, df = 5 (p = 0.02)
0.2 0.4 0.6 0.8 1
Specificity

③DOR

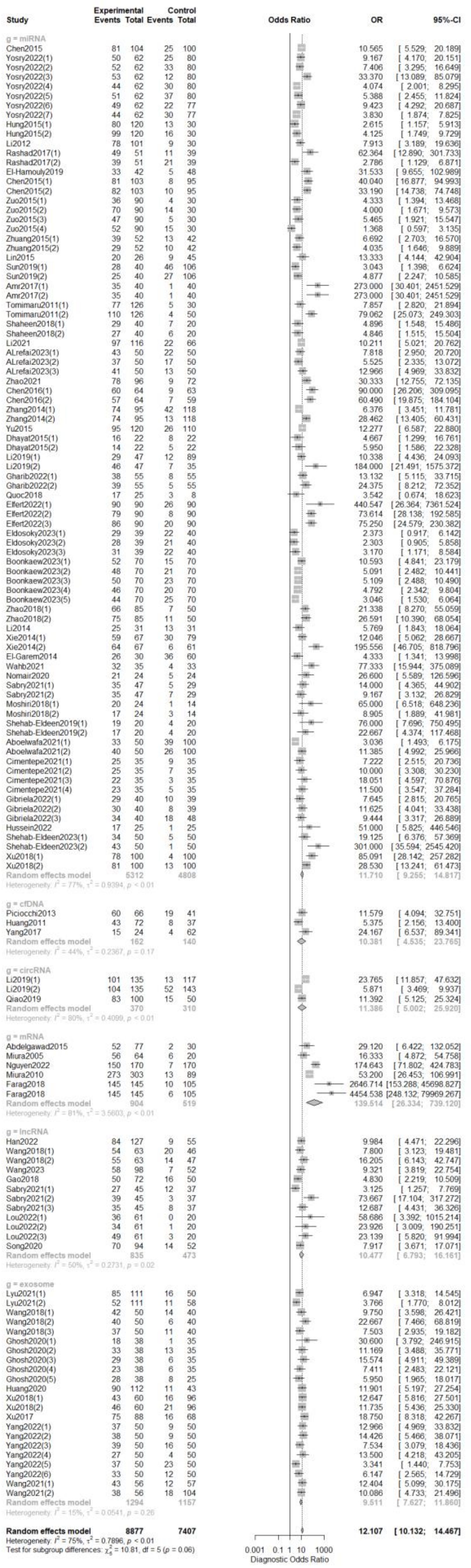

Subgroup analysis

3. biomarkers in HCC vs. healthy population (based on optimal circRNA)

①Sensitivity analysis

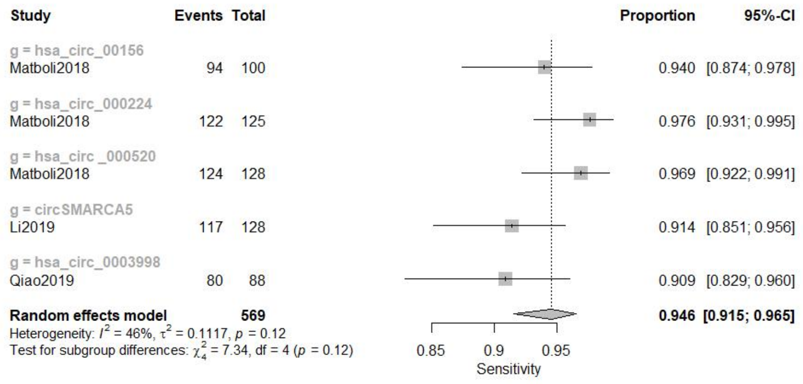

②Specificity analysis

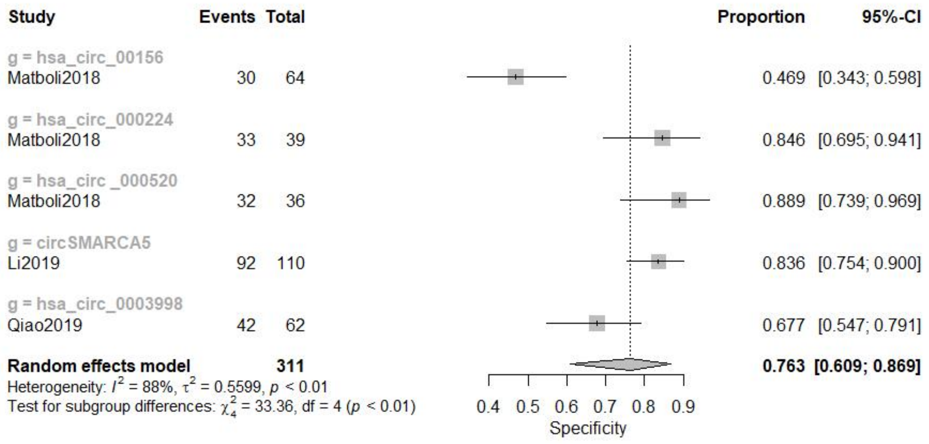

③DOR

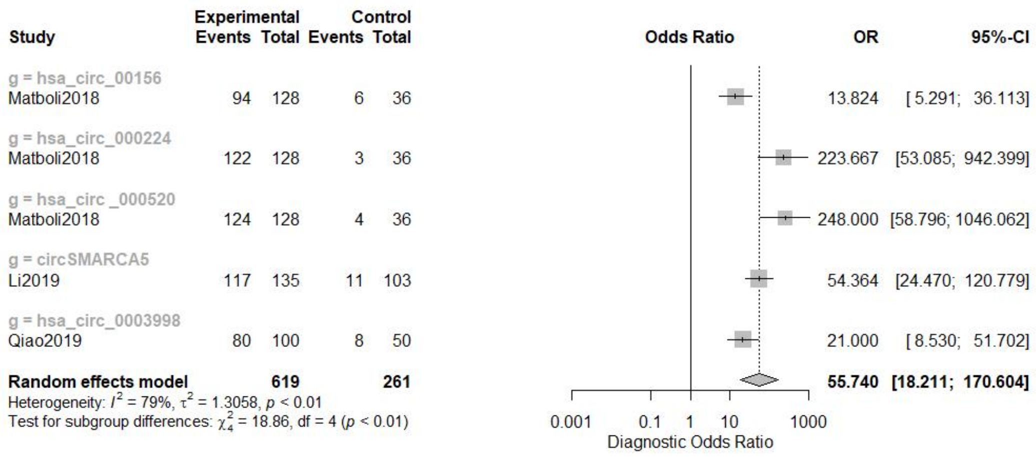

4. biomarkers in HCC vs. Liver disease (based on optimal circRNA)

①Sensitivity analysis

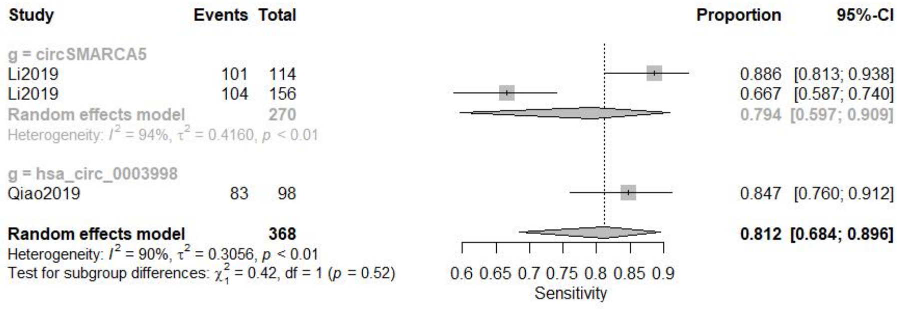

②Specificity analysis

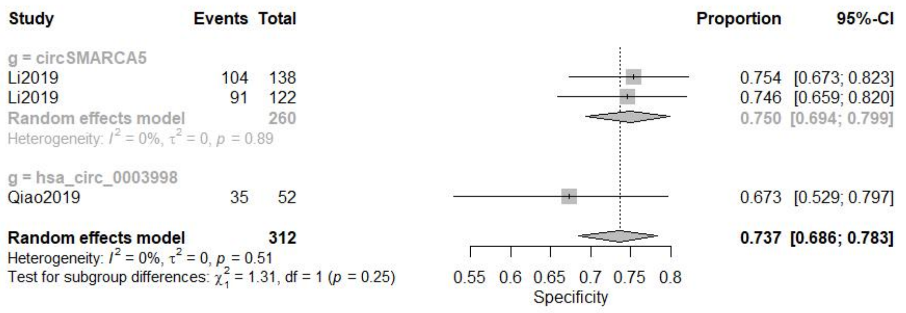

③DOR

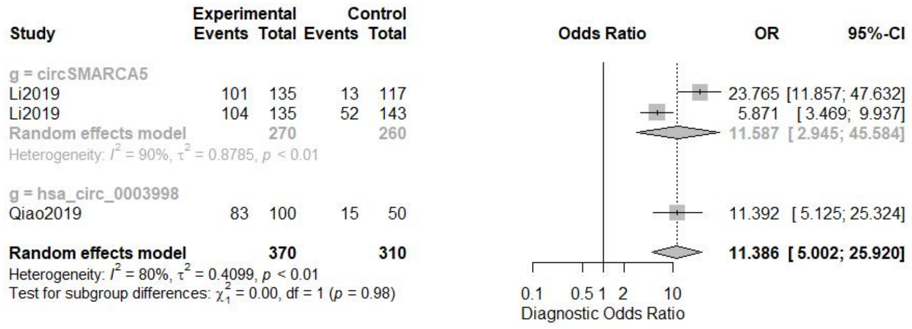

5. biomarkers in HCC vs. healthy population (based on optimal mRNA)

①Sensitivity analysis

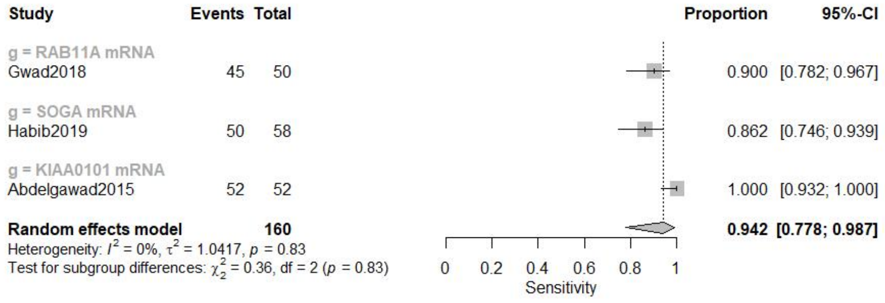

②Specificity analysis

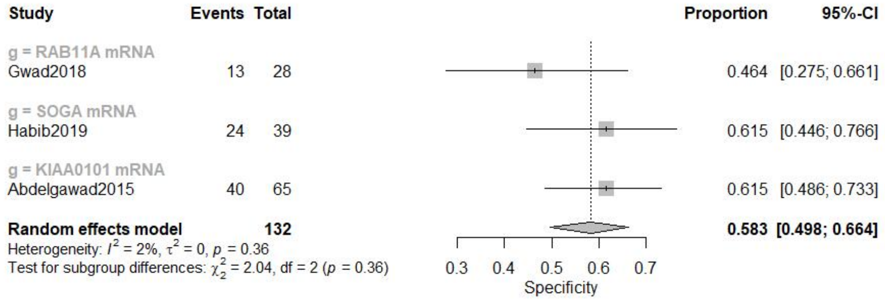

③DOR

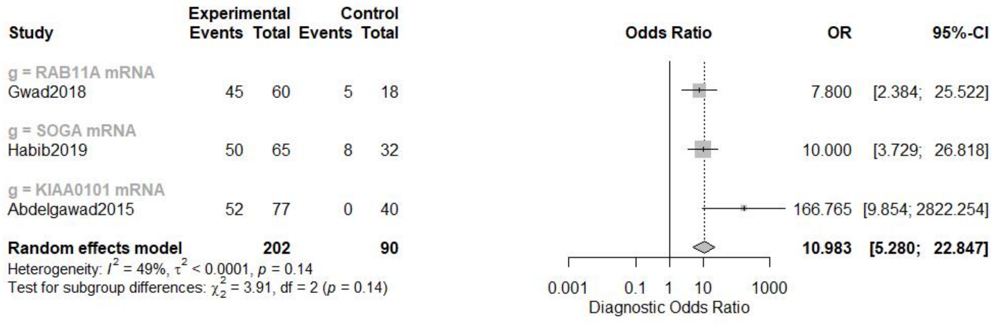

6. biomarkers in HCC vs. Liver disease (based on optimal mRNA)

①Sensitivity analysis

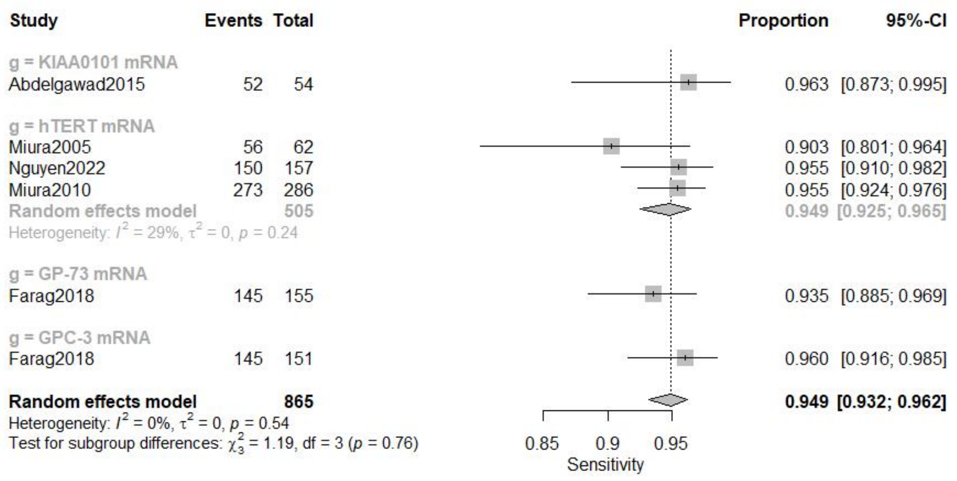

②Specificity analysis

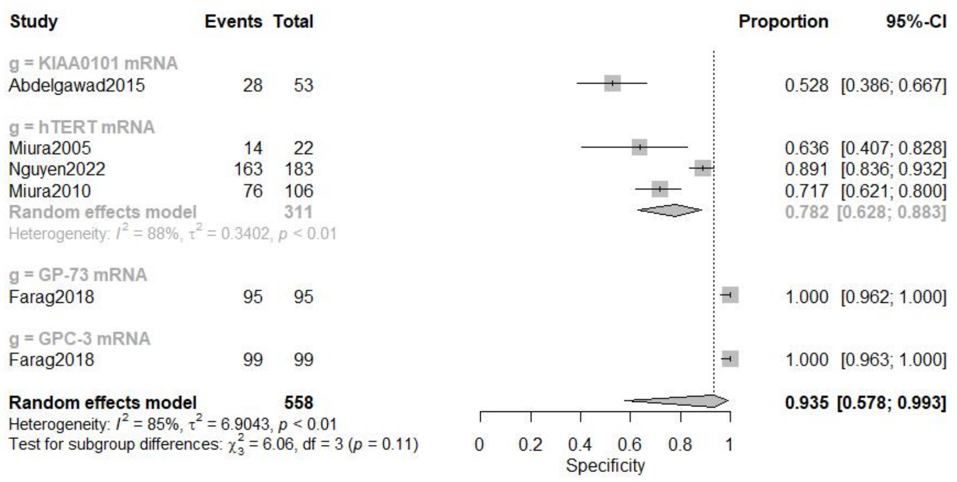

③DOR

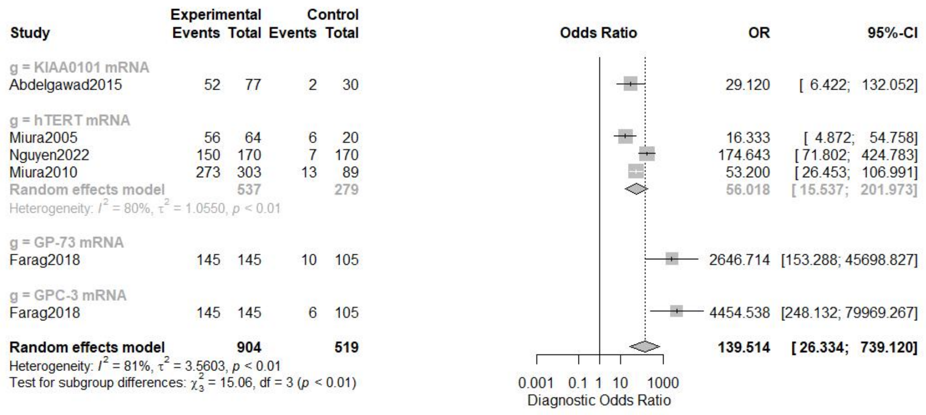

7. biomarkers in HCC vs. healthypopulation (based on optimal exosome)

①Sensitivity analysis

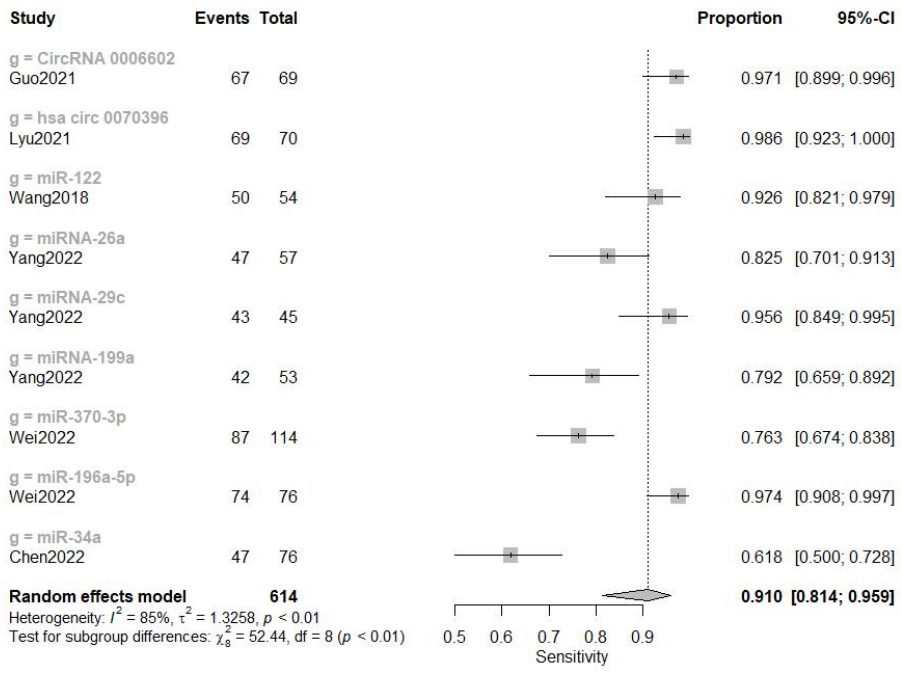

②Specificity analysis

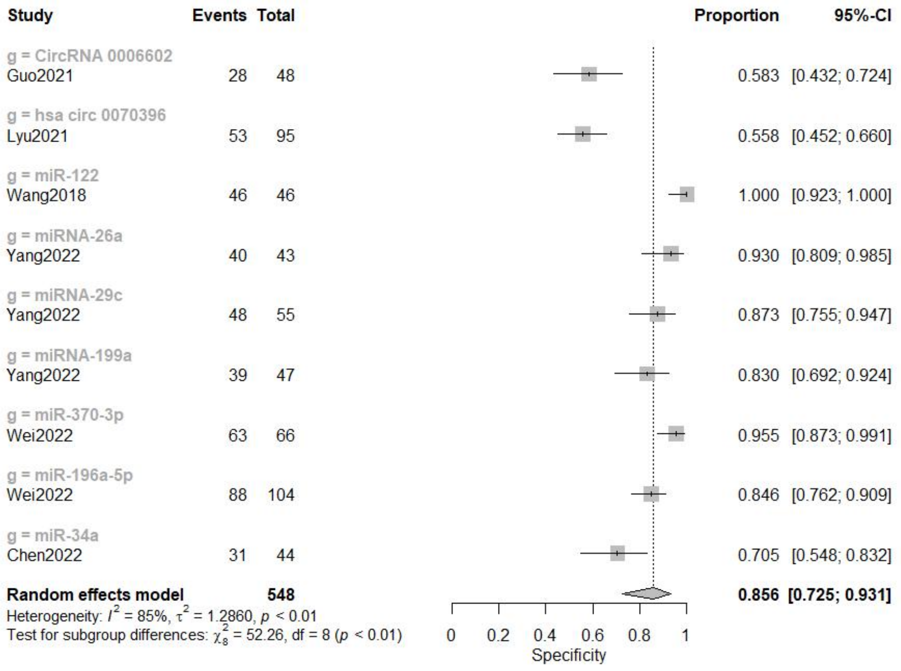

③DOR

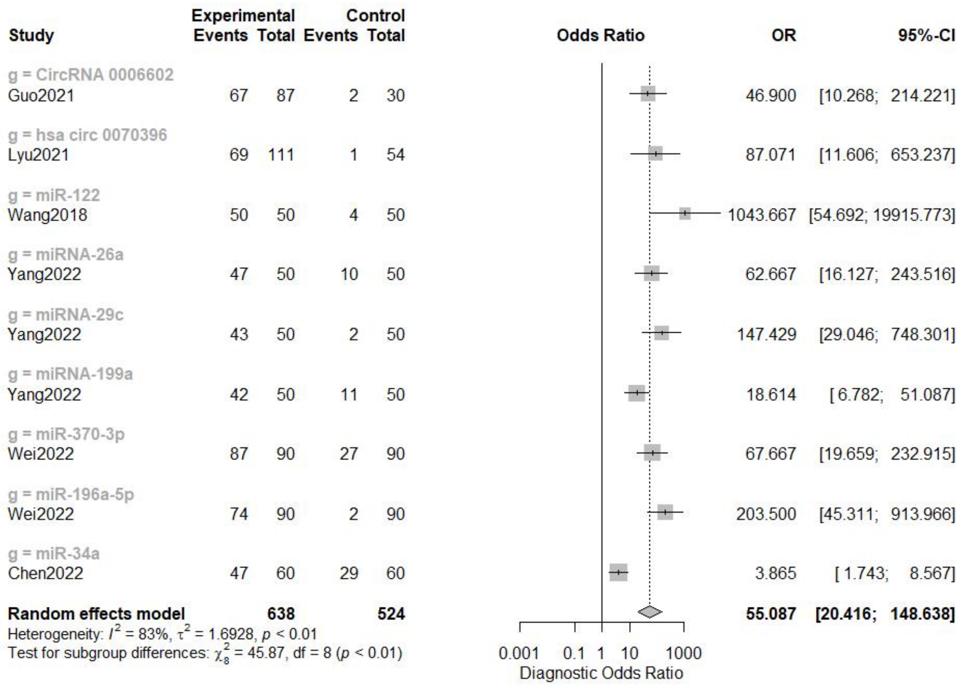

8. biomarkers in HCC vs. Liver disease (based on optimal exosome)

①Sensitivity analysis

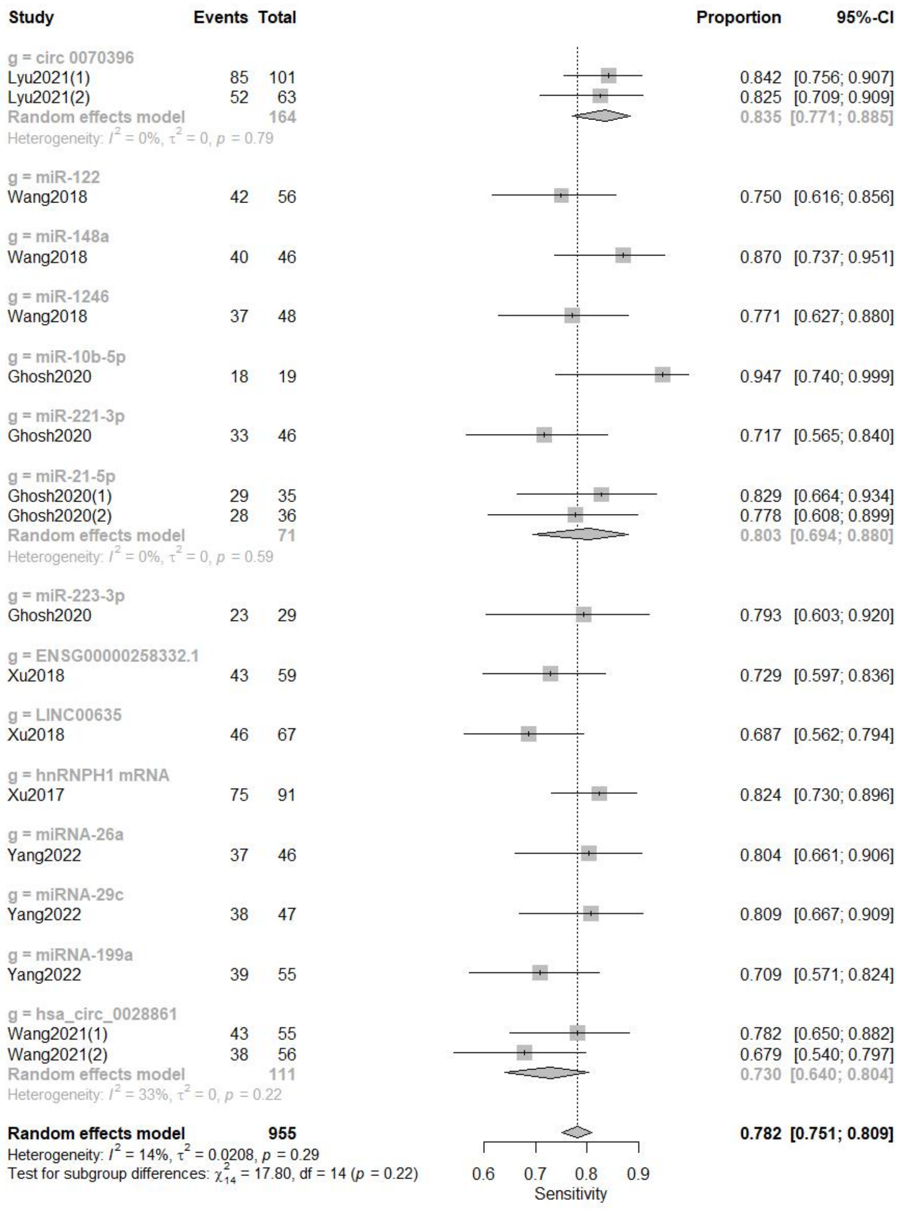

②Specificity analysis

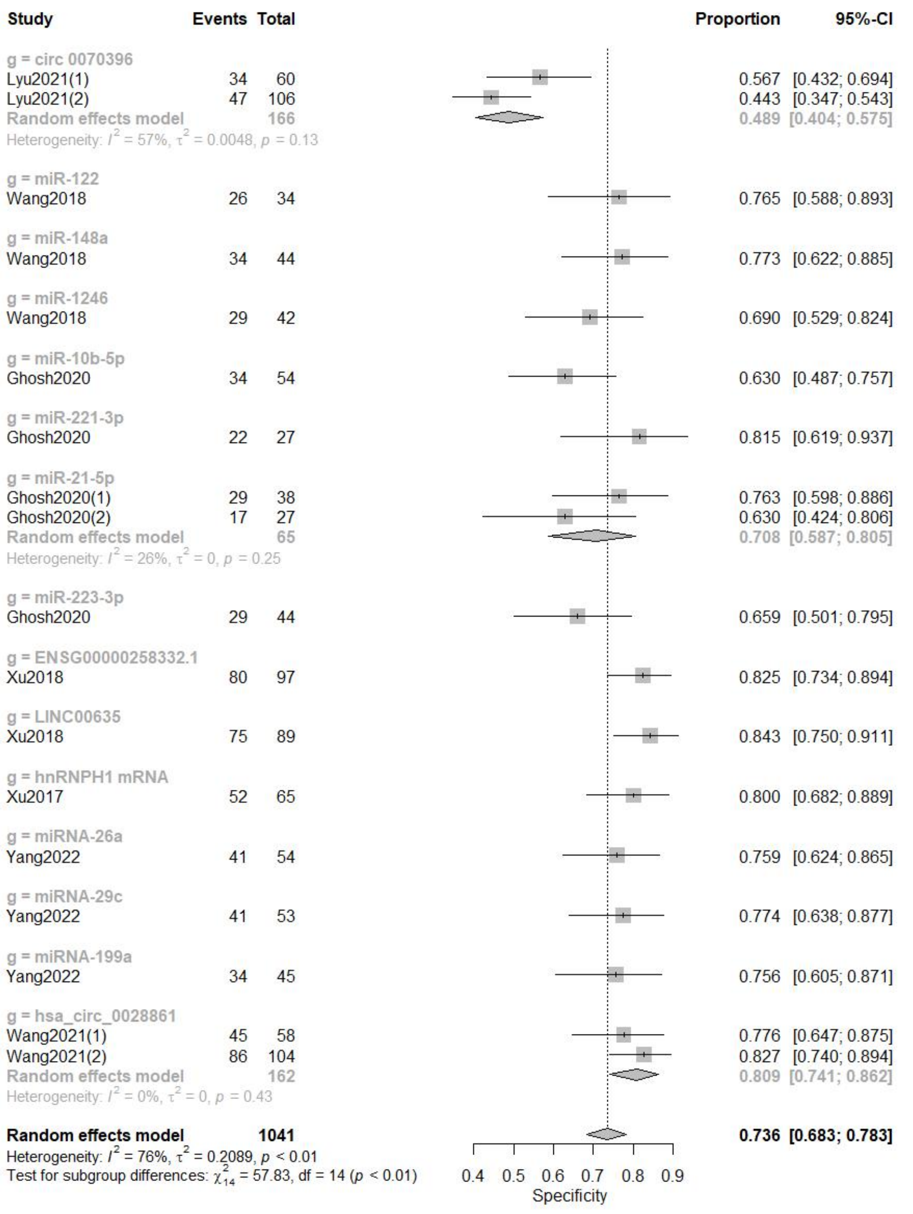

③DOR

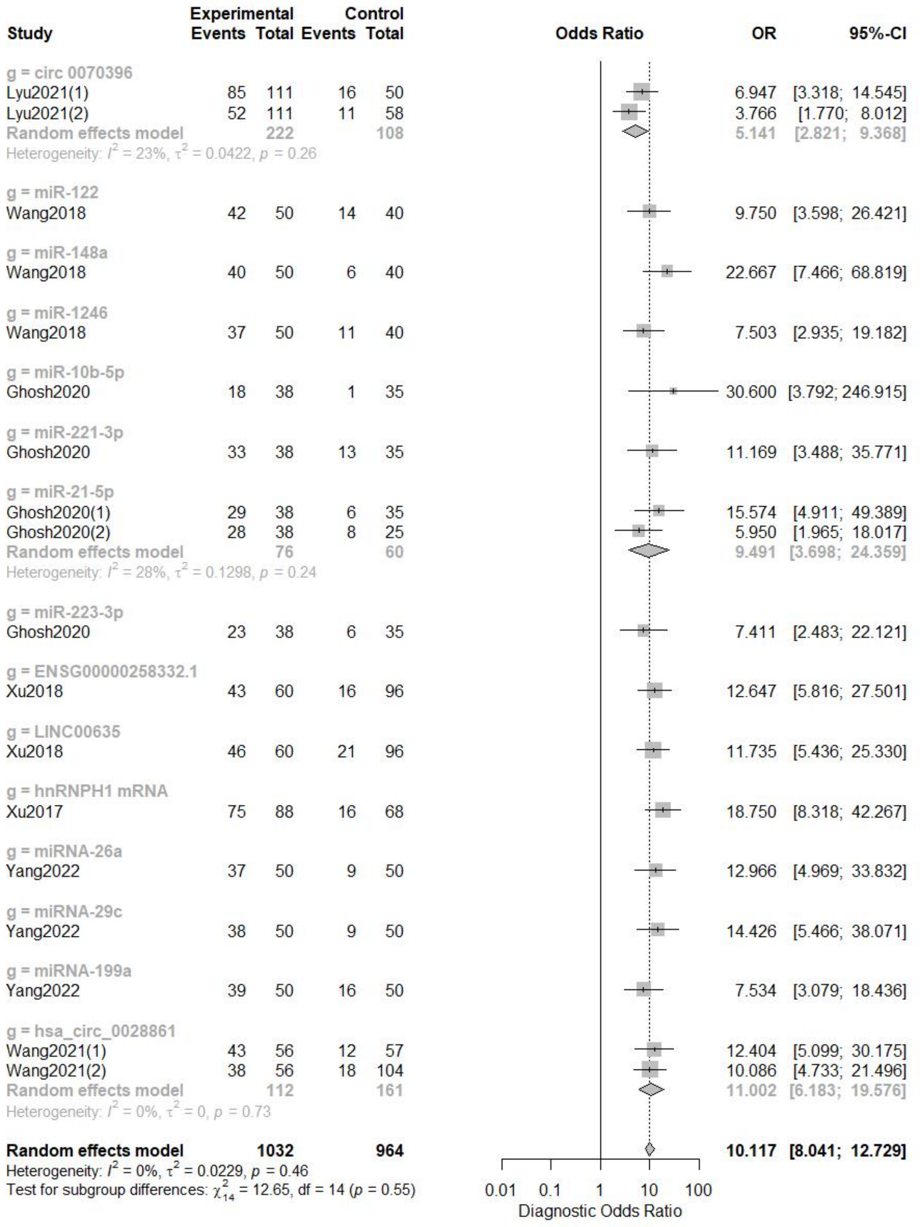

9. biomarkers in HCC vs. healthy population(based on optimal miRNA)

①Sensitivity analysis

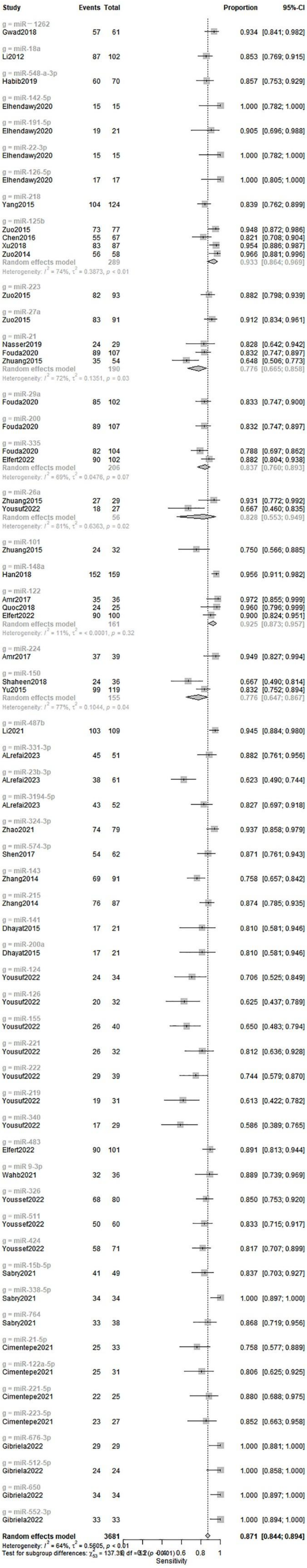

Study Events Total Proportion 95%-CI
g = miR−1262
Gwad2018 57 61 0.934 [0.841; 0.982]
g = miR-18a
Li2012 87 102 0.853 [0.769; 0.915]
g = miR-548-a-3p
Habib2019 60 70 0.857 [0.753; 0.929]
g = miR-142-5p
Elhendawy2020 15 15 1.000 [0.782; 1.000]
g = miR-191-5p
Elhendawy2020 19 21 0.905 [0.696; 0.988]
g = miR-22-3p
Elhendawy2020 15 15 1.000 [0.782; 1.000]
g = miR-126-5p
Elhendawy2020 17 17 1.000 [0.805; 1.000]
g = miR-218
Yang2015 104 124 0.839 [0.762; 0.899]
g = miR-125b
Zuo2015 73 77 0.948 [0.872; 0.986]
Chen2016 55 67 0.821 [0.708; 0.904]
Xu2018 83 87 0.954 [0.886; 0.987]
Zuo2014 56 58 0.966 [0.881; 0.996]
Random effects model 289 0.933 [0.864; 0.969]
Heterogeneity: I^2 = 74%, τ^2 = 0.3873, p < 0.01
g = miR-223
Zuo2015 82 93 0.882 [0.798; 0.939]
g = miR-27a
Zuo2015 83 91 0.912 [0.834; 0.961]
g = miR-21
Nasser2019 24 29 0.828 [0.642; 0.942]
Fouda2020 89 107 0.832 [0.747; 0.897]
Zhuang2015 35 54 0.648 [0.506; 0.773]
Random effects model 190 0.776 [0.665; 0.858]
Heterogeneity: I^2 = 72%, τ^2 = 0.1351, p = 0.03
g = miR-29a
Fouda2020 85 102 0.833 [0.747; 0.900]
g = miR-200
Fouda2020 89 107 0.832 [0.747; 0.897]
g = miR-335
Fouda2020 82 104 0.788 [0.697; 0.862]
Elfert2022 90 102 0.882 [0.804; 0.938]
Random effects model 206 0.837 [0.760; 0.893]
Heterogeneity: I^2 = 69%, τ^2 = 0.0476, p = 0.07
g = miR-26a
Zhuang2015 27 29 0.931 [0.772; 0.992]
Yousuf2022 18 27 0.667 [0.460; 0.835]
Random effects model 56 0.828 [0.553; 0.949]
Heterogeneity: I^2 = 81%, τ^2 = 0.6363, p = 0.02
g = miR-101
Zhuang2015 24 32 0.750 [0.566; 0.885]
g = miR-148a
Han2018 152 159 0.956 [0.911; 0.982]
g = miR-122
Amr2017 35 36 0.972 [0.855; 0.999]
Quoc2018 24 25 0.960 [0.796; 0.999]
Elfert2022 90 100 0.900 [0.824; 0.951]
Random effects model 161 0.925 [0.873; 0.957]
Heterogeneity: I^2 = 11%, τ^2 = < 0.0001, p = 0.32
g = miR-224
Amr2017 37 39 0.949 [0.827; 0.994]
g = miR-150
Shaheen2018 24 36 0.667 [0.490; 0.814]
Yu2015 99 119 0.832 [0.752; 0.894]
Random effects model 155 0.776 [0.647; 0.867]
Heterogeneity: I^2 = 77%, τ^2 = 0.1044, p = 0.04
g = miR-487b
Li2021 103 109 0.945 [0.884; 0.980]
g = miR-331-3p
ALrefai2023 45 51 0.882 [0.761; 0.956]
g = miR-23b-3p
ALrefai2023 38 61 0.623 [0.490; 0.744]
g = miR-3194-5p
ALrefai2023 43 52 0.827 [0.697; 0.918]
g = miR-324-3p
Zhao2021 74 79 0.937 [0.858; 0.979]
g = miR-574-3p
Shen2017 54 62 0.871 [0.761; 0.943]
g = miR-143
Zhang2014 69 91 0.758 [0.657; 0.842]
g = miR-215
Zhang2014 76 87 0.874 [0.785; 0.935]
g = miR-141
Dhayat2015 17 21 0.810 [0.581; 0.946]
g = miR-200a
Dhayat2015 17 21 0.810 [0.581; 0.946]
g = miR-124
Yousuf2022 24 34 0.706 [0.525; 0.849]
g = miR-126
Yousuf2022 20 32 0.625 [0.437; 0.789]
g = miR-155
Yousuf2022 26 40 0.650 [0.483; 0.794]
g = miR-221
Yousuf2022 26 32 0.812 [0.636; 0.928]
g = miR-222
Yousuf2022 29 39 0.744 [0.579; 0.870]
g = miR-219
Yousuf2022 19 31 0.613 [0.422; 0.782]
g = miR-340
Yousuf2022 17 29 0.586 [0.389; 0.765]
g = miR-483
Elfert2022 90 101 0.891 [0.813; 0.944]
g = miR 9-3p
Wahb2021 32 36 0.889 [0.739; 0.969]
g = miR-326
Youssef2022 68 80 0.850 [0.753; 0.920]
g = miR-511
Youssef2022 50 60 0.833 [0.715; 0.917]
g = miR-424
Youssef2022 58 71 0.817 [0.707; 0.899]
g = miR-15b-5p
Sabry2021 41 49 0.837 [0.703; 0.927]
g = miR-338-5p
Sabry2021 34 34 1.000 [0.897; 1.000]
g = miR-764
Sabry2021 33 38 0.868 [0.719; 0.956]
g = miR-21-5p
Cimentepe2021 25 33 0.758 [0.577; 0.889]
g = miR-122a-5p
Cimentepe2021 25 31 0.806 [0.625; 0.925]
g = miR-221-5p
Cimentepe2021 22 25 0.880 [0.688; 0.975]
g = miR-223-5p
Cimentepe2021 23 27 0.852 [0.663; 0.958]
g = miR-676-3p
Gibriela2022 29 29 1.000 [0.881; 1.000]
g = miR-512-5p
Gibriela2022 24 24 1.000 [0.858; 1.000]
g = miR-650
Gibriela2022 34 34 1.000 [0.897; 1.000]
g = miR-552-3p
Gibriela2022 33 33 1.000 [0.894; 1.000]
Random effects model 3681 0.871 [0.844; 0.894]
Heterogeneity: I^2 = 64%, τ^2 = 0.5605, p < 0.01
Test for subgroup differences
0.6 0.8 1
Sensitivity

②Specificity analysis

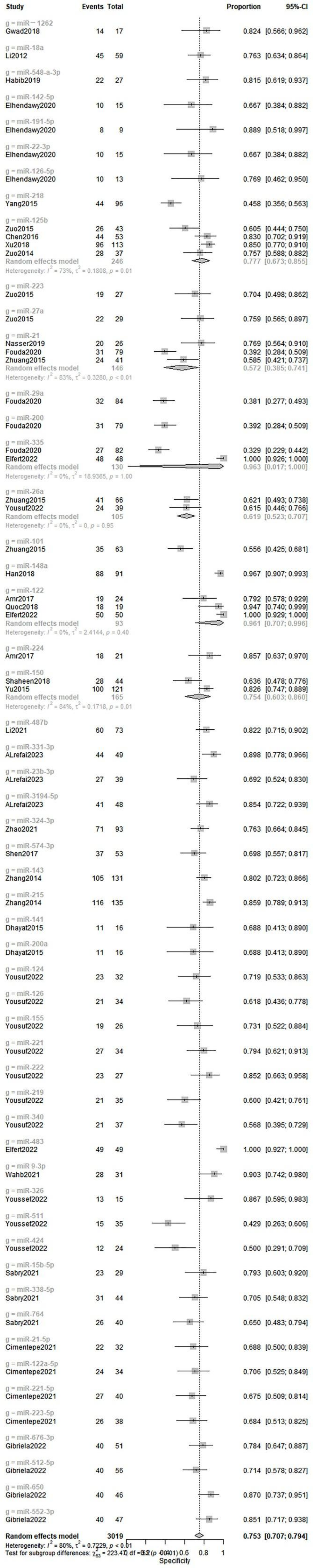

③DOR

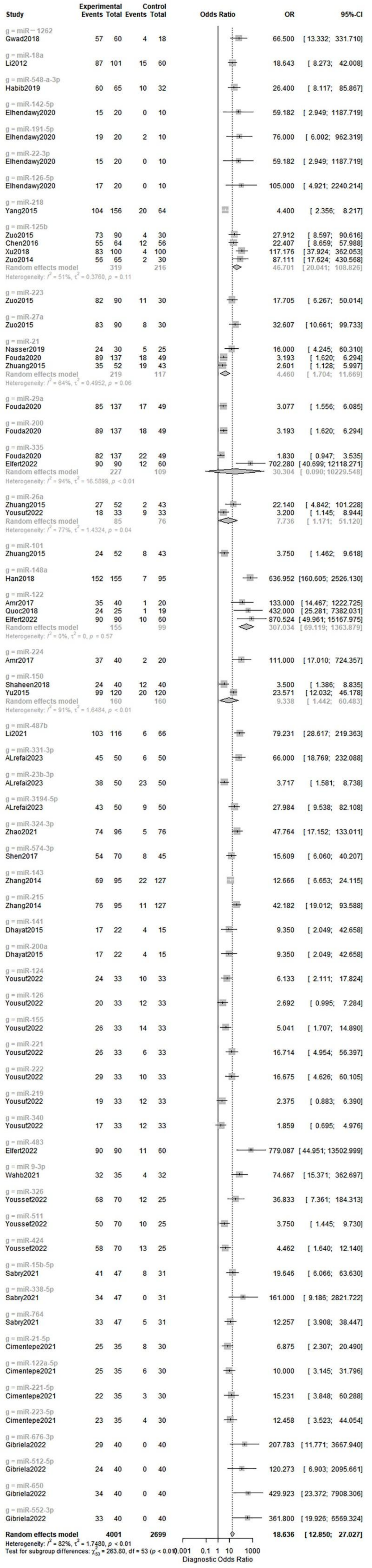
Study
Experimental Events Total
Control Events Total
Odds Ratio
OR
95%-CI
g = miR−1262
Gwad2018 57 60 4 18 66.500 [13.332; 331.710]
g = miR-18a
Li2012 87 101 15 60 18.643 [8.273; 42.008]
g = miR-548-a-3p
Habib2019 60 65 10 32 26.400 [8.117; 85.867]
g = miR-142-5p
Elhendawy2020 15 20 0 10 59.182 [2.949; 1187.719]
g = miR-191-5p
Elhendawy2020 19 20 2 10 76.000 [6.002; 962.319]
g = miR-22-3p
Elhendawy2020 15 20 0 10 59.182 [2.949; 1187.719]
g = miR-126-5p
Elhendawy2020 17 20 0 10 105.000 [4.921; 2240.214]
g = miR-218
Yang2015 104 156 20 64 4.400 [2.356; 8.217]
g = miR-125b
Zuo2015 73 90 4 30 27.912 [8.597; 90.616]
Chen2016 55 64 12 56 22.407 [8.659; 57.988]
Xu2018 83 100 4 100 117.176 [37.924; 362.053]
Zuo2014 56 65 2 30 87.111 [17.624; 430.568]
Random effects model 319 216 46.701 [20.041; 108.826]
Heterogeneity: I^2 = 51%, τ^2 = 0.3760, p = 0.11
g = miR-223
Zuo2015 82 90 11 30 17.705 [6.267; 50.014]
g = miR-27a
Zuo2015 83 90 8 30 32.607 [10.661; 99.733]
g = miR-21
Nasser2019 24 30 5 25 16.000 [4.245; 60.310]
Fouda2020 89 137 18 49 3.193 [1.620; 6.294]
Zhuang2015 35 52 19 43 2.601 [1.128; 5.997]
Random effects model 219 117 4.460 [1.704; 11.669]
Heterogeneity: I^2 = 64%, τ^2 = 0.4952, p = 0.06
g = miR-29a
Fouda2020 85 137 17 49 3.077 [1.556; 6.085]
g = miR-200
Fouda2020 89 137 18 49 3.193 [1.620; 6.294]
g = miR-335
Fouda2020 82 137 22 49 1.830 [0.947; 3.535]
Elfert2022 90 90 12 60 702.280 [40.699; 12118.271]
Random effects model 227 109 30.304 [0.090; 10229.548]
Heterogeneity: I^2 = 94%, τ^2 = 16.5899, p < 0.01
g = miR-26a
Zhuang2015 27 52 2 43 22.140 [4.842; 101.228]
Yousuf2022 18 33 9 33 3.200 [1.145; 8.944]
Random effects model 85 76 7.736 [1.171; 51.120]
Heterogeneity: I^2 = 77%, τ^2 = 1.4324, p = 0.04
g = miR-101
Zhuang2015 24 52 8 43 3.750 [1.462; 9.618]
g = miR-148a
Han2018 152 155 7 95 636.952 [160.605; 2526.130]
g = miR-122
Amr2017 35 40 1 20 133.000 [14.467; 1222.725]
Quoc2018 24 25 1 19 432.000 [25.281; 7382.031]
Elfert2022 90 90 10 60 870.524 [49.961; 15167.975]
Random effects model 155 99 307.034 [69.119; 1363.879]
Heterogeneity: I^2 = 0%, τ^2 = 0, p = 0.57
g = miR-224
Amr2017 37 40 2 20 111.000 [17.010; 724.357]
g = miR-150
Shaheen2018 24 40 12 40 3.500 [1.386; 8.835]
Yu2015 99 120 20 120 23.571 [12.032; 46.178]
Random effects model 160 160 9.338 [1.442; 60.483]
Heterogeneity: I^2 = 91%, τ^2 = 1.6484, p < 0.01
g = miR-487b
Li2021 103 116 6 66 79.231 [28.617; 219.363]
g = miR-331-3p
ALrefai2023 45 50 6 50 66.000 [18.769; 232.088]
g = miR-23b-3p
ALrefai2023 38 50 23 50 3.717 [1.581; 8.738]
g = miR-3194-5p
ALrefai2023 43 50 9 50 27.984 [9.538; 82.108]
g = miR-324-3p
Zhao2021 74 96 5 76 47.764 [17.152; 133.011]
g = miR-574-3p
Shen2017 54 70 8 45 15.609 [6.060; 40.207]
g = miR-143
Zhang2014 69 95 22 127 12.666 [6.653; 24.115]
g = miR-215
Zhang2014 76 95 11 127 42.182 [19.012; 93.588]
g = miR-141
Dhayat2015 17 22 4 15 9.350 [2.049; 42.658]
g = miR-200a
Dhayat2015 17 22 4 15 9.350 [2.049; 42.658]
g = miR-124
Yousuf2022 24 33 10 33 6.133 [2.111; 17.824]
g = miR-126
Yousuf2022 20 33 12 33 2.692 [0.995; 7.284]
g = miR-155
Yousuf2022 26 33 14 33 5.041 [1.707; 14.890]
g = miR-221
Yousuf2022 26 33 6 33 16.714 [4.954; 56.397]
g = miR-222
Yousuf2022 29 33 10 33 16.675 [4.626; 60.105]
g = miR-219
Yousuf2022 19 33 12 33 2.375 [0.883; 6.390]
g = miR-340
Yousuf2022 17 33 12 33 1.859 [0.695; 4.976]
g = miR-483
Elfert2022 90 90 11 60 779.087 [44.951; 13502.999]
g = miR 9-3p
Wahb2021 32 35 4 32 74.667 [15.371; 362.697]
g = miR-326
Youssef2022 68 70 12 25 36.833 [7.361; 184.313]
g = miR-511
Youssef2022 50 70 10 25 3.750 [1.445; 9.730]
g = miR-424
Youssef2022 58 70 13 25 4.462 [1.640; 12.140]
g = miR-15b-5p
Sabry2021 41 47 8 31 19.646 [6.066; 63.630]
g = miR-338-5p
Sabry2021 34 47 0 31 161.000 [9.186; 2821.722]
g = miR-764
Sabry2021 33 47 5 31 12.257 [3.908; 38.447]
g = miR-21-5p
Cimentepe2021 25 35 8 30 6.875 [2.307; 20.490]
g = miR-122a-5p
Cimentepe2021 25 35 6 30 10.000 [3.145; 31.796]
g = miR-221-5p
Cimentepe2021 22 35 3 30 15.231 [3.848; 60.288]
g = miR-223-5p
Cimentepe2021 23 35 4 30 12.458 [3.523; 44.054]
g = miR-676-3p
Gibriela2022 29 40 0 40 207.783 [11.771; 3667.940]
g = miR-512-5p
Gibriela2022 24 40 0 40 120.273 [6.903; 2095.661]
g = miR-650
Gibriela2022 34 40 0 40 429.923 [23.372; 7908.306]
g = miR-552-3p
Gibriela2022 33 40 0 40 361.800 [19.926; 6569.324]
Random effects model 4001 2699 18.636 [12.850; 27.027]
Heterogeneity: I^2 = 82%, τ^2 = 1.7480, p < 0.01
Test for subgroup differences: χ^2_{53} = 263.80, df = 53 (p < 0.01)
0.001 0.1 1 10 1000
Diagnostic Odds Ratio

10. biomarkers in HCC vs. Liver disease (based on optimal miRNA)

①Sensitivity analysis

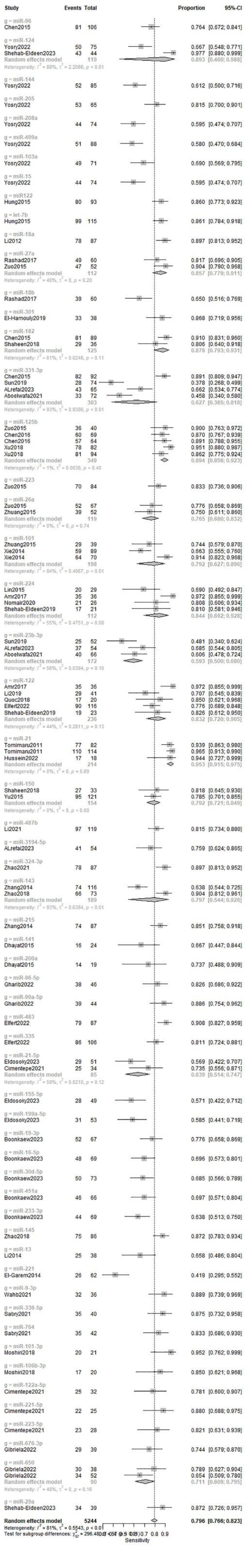

②Specificity analysis

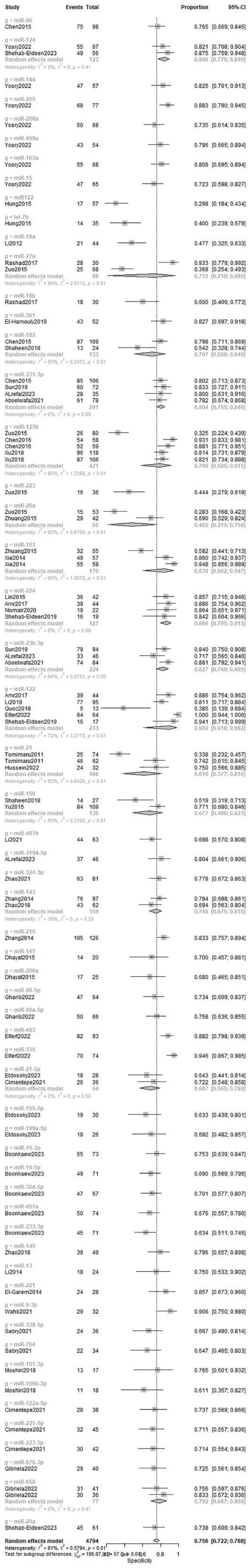

③DOR

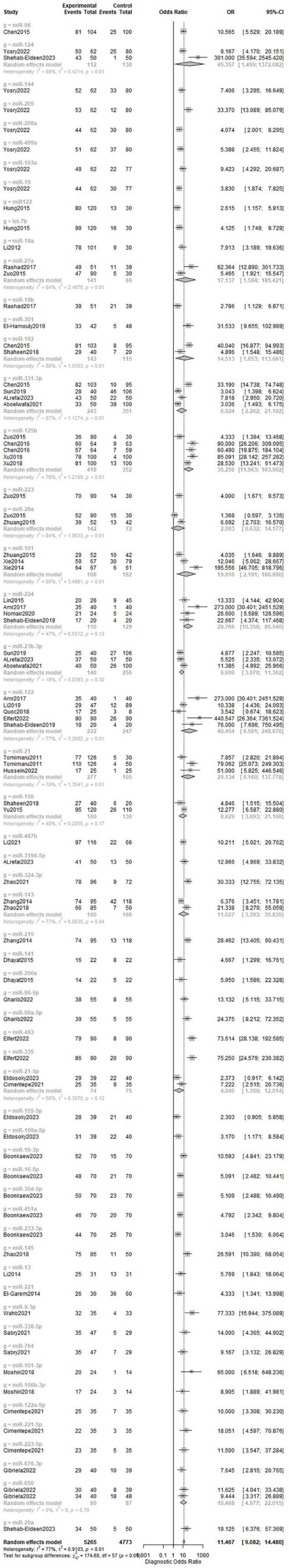
Study
Experimental Events Total
Control Events Total
Odds Ratio
OR
95%-CI
Random effects model
Diagnostic Odds Ratio
