# Supplementary material for: Diagnosis of hepatocellular carcinoma using liquid biopsy-based biomarkers: a systematic review and network meta-analysis
Source: Front Oncol. 2025 Jan 28;14:1483521. doi: 10.3389/fonc.2024.1483521 (PMC11810725; doi:10.3389/fonc.2024.1483521)
Supplement: Supplementary Table 4 — Quality evaluation of included studies. [file Table1.docx]

**Table S1** Search strategy

**Cochrane：**

Search Name:

Date Run: 11/01/2024 14:32:01

Comment:

ID Search Hits

#1 ('hepatic cellular inflammation' OR 'hepatic inflammation' OR 'hepatic inflammatory disease' OR 'hepatitic disease' OR 'hepatitic disorder' OR 'hepatitic illness' OR 'hepatitides' OR 'hepatocellular inflammation' OR 'hepatocellular inflammatory disease' OR 'hepatocellular inflammatory syndrome' OR 'hepatoinflammation' OR 'liver infection' OR 'liver inflammation' OR 'liver inflammatory disease' OR 'subacute hepatitis' OR 'hepatitis'):ti,ab,kw 31690

#2 MeSH descriptor: [Hepatitis] explode all trees 8452

#3 ('Hepatitis' OR 'Hepatitides' OR 'hepatic cellular inflammation' OR 'hepatic inflammation' OR 'hepatic inflammatory disease' OR 'hepatitic disease' OR 'hepatitic disorder' OR 'hepatitic illness' OR 'hepatocellular inflammation' OR 'hepatocellular inflammatory disease' OR 'hepatocellular inflammatory syndrome' OR 'hepatoinflammation' OR 'liver infection' OR 'liver inflammation' OR 'liver inflammatory disease' OR 'subacute hepatitis' OR 'cirrhosis' OR 'cirrhosis hepatis' OR 'cirrhosis, liver' OR 'cryptogenic liver cirrhosis' OR 'dietary cirrhosis' OR 'dietary liver cirrhosis' OR 'hepatic cirrhosis' OR 'postnecrotic liver cirrhosis' OR 'liver cirrhosis' OR 'Fibrosis, Liver' OR 'Liver Fibrosis'):ti,ab,kw 39787

#4 MeSH descriptor: [Liver Cirrhosis] explode all trees 4422

#5 ('Liquid Biopsy' OR 'Liquid biops*' OR 'cfDNA' OR 'circulating cell-free DNA' OR 'circulating tumor DNA' OR 'circulating DNA' OR 'ctDNA' OR 'plasma DNA' OR 'serum DNA' OR 'blood DNA' OR 'circulating microRNA' OR 'microRNA' OR 'miRNA' OR 'miR' OR 'circulating tumor cell' OR 'CTC' OR 'Exosome*' OR 'Exosomic' OR 'Exosomal' OR 'Extracellular vesicle*' OR 'Microvesicle*' OR 'alpha fetoprotein' OR 'AFP'):ti,ab,kw 17188

#6 MeSH descriptor: [Liquid Biopsy] explode all trees 33

#7 ('liver cell carcinoma*' OR 'liver cell cancer' OR 'Hepatocellular Cancer*' OR 'hepatocellular carcinoma*' OR 'hepatocarcinoma' OR 'hepatic cell carcinoma' OR 'hepato-cellular carcinoma' OR 'hepatocyte carcinoma'):ti,ab,kw 9915

#8 MeSH descriptor: [Carcinoma, Hepatocellular] explode all trees 2447

#9 ('diagnosis' OR 'sensitivity' OR 'specificity' OR 'accuracy' OR 'receiver operating characteristic'):ti,ab,kw 265717

#10 MeSH descriptor: [Diagnosis] explode all trees 448828

#11 (#1 OR #2 OR #3 OR #4) AND (#5 OR #6) AND (#7 OR #8) AND (#9 OR #10) 229

**EMBASE：**

| No. | Query | Results | Date |
| --- | --- | --- | --- |
| #11 | (#1 OR #2 OR #3 OR #4) AND (#5 OR #6) AND (#7 OR #8) AND (#9 OR #10) AND [2000-2024]/py | 6829 | 11-Jan-24 |
| #10 | 'diagnosis':ti,ab,kw OR 'sensitivity':ti,ab,kw OR 'specificity':ti,ab,kw OR 'accuracy':ti,ab,kw OR 'receiver operating characteristic':ti,ab,kw | 4813335 | 11-Jan-24 |
| #9 | 'diagnosis'/exp | 8596316 | 11-Jan-24 |
| #8 | 'liver cell carcinoma*':ti,ab,kw OR 'liver cell cancer':ti,ab,kw OR 'hepatocellular cancer*':ti,ab,kw OR 'hepatocellular carcinoma*':ti,ab,kw OR 'hepatocarcinoma':ti,ab,kw OR 'hepatic cell carcinoma':ti,ab,kw OR 'hepato-cellular carcinoma':ti,ab,kw OR 'hepatocyte carcinoma':ti,ab,kw | 184655 | 11-Jan-24 |
| #7 | 'liver cell carcinoma'/exp | 214774 | 11-Jan-24 |
| #6 | 'liquid biopsy':ti,ab,kw OR 'liquid biops*':ti,ab,kw OR 'cfdna':ti,ab,kw OR 'circulating cell-free dna':ti,ab,kw OR 'circulating tumor dna':ti,ab,kw OR 'circulating dna':ti,ab,kw OR 'ctdna':ti,ab,kw OR 'plasma dna':ti,ab,kw OR 'serum dna':ti,ab,kw OR 'blood dna':ti,ab,kw OR 'circulating microrna':ti,ab,kw OR 'microrna':ti,ab,kw OR 'mirna':ti,ab,kw OR 'mir':ti,ab,kw OR 'circulating tumor cell':ti,ab,kw OR 'ctc':ti,ab,kw OR 'exosome*':ti,ab,kw OR 'exosomic':ti,ab,kw OR 'exosomal':ti,ab,kw OR 'extracellular vesicle*':ti,ab,kw OR 'microvesicle*':ti,ab,kw OR 'alpha fetoprotein':ti,ab,kw OR 'afp':ti,ab,kw | 347825 | 11-Jan-24 |
| #5 | 'liquid biopsy'/exp | 12008 | 11-Jan-24 |
| #4 | 'cirrhosis':ti,ab,kw OR 'cirrhosis hepatis':ti,ab,kw OR 'cirrhosis, liver':ti,ab,kw OR 'cryptogenic liver cirrhosis':ti,ab,kw OR 'dietary cirrhosis':ti,ab,kw OR 'dietary liver cirrhosis':ti,ab,kw OR 'hepatic cirrhosis':ti,ab,kw OR 'postnecrotic liver cirrhosis':ti,ab,kw OR 'liver cirrhosis':ti,ab,kw | 180030 | 11-Jan-24 |
| #3 | 'liver cirrhosis'/exp | 210985 | 11-Jan-24 |
| #2 | 'hepatic cellular inflammation':ti,ab,kw OR 'hepatic inflammation':ti,ab,kw OR 'hepatic inflammatory disease':ti,ab,kw OR 'hepatitic disease':ti,ab,kw OR 'hepatitic disorder':ti,ab,kw OR 'hepatitic illness':ti,ab,kw OR 'hepatitides':ti,ab,kw OR 'hepatocellular inflammation':ti,ab,kw OR 'hepatocellular inflammatory disease':ti,ab,kw OR 'hepatocellular inflammatory syndrome':ti,ab,kw OR 'hepatoinflammation':ti,ab,kw OR 'liver infection':ti,ab,kw OR 'liver inflammation':ti,ab,kw OR 'liver inflammatory disease':ti,ab,kw OR 'subacute hepatitis':ti,ab,kw OR 'hepatitis':ti,ab,kw | 366836 | 11-Jan-24 |
| #1 | 'hepatitis'/exp OR 'hepatitis' | 519058 | 11-Jan-24 |

**PUBMED：**

Search number Query Sort By Filters Search Details Results Time

12 (#1 OR #2 OR #3 OR #4) AND (#5 OR #6) AND (#7 OR #8) AND (#9 OR #10) from 2000 - 2024 (("hepatitis"[MeSH Terms] OR "hepatitis a"[MeSH Terms] OR ("hepatic inflammation"[Title/Abstract] OR "hepatic inflammatory disease"[Title/Abstract] OR "hepatitic disease"[Title/Abstract] OR "hepatitic illness"[Title/Abstract] OR "hepatitides"[Title/Abstract] OR "hepatocellular inflammation"[Title/Abstract] OR "liver infection"[Title/Abstract] OR "liver inflammation"[Title/Abstract] OR "liver inflammatory disease"[Title/Abstract] OR "subacute hepatitis"[Title/Abstract] OR "hepatitis"[Title/Abstract]) OR "liver cirrhosis"[MeSH Terms] OR ("fibrosis liver"[Title/Abstract] OR "Liver Fibrosis"[Title/Abstract] OR "cirrhosis"[Title/Abstract] OR "cirrhosis hepatis"[Title/Abstract] OR "cirrhosis liver"[Title/Abstract] OR "cryptogenic liver cirrhosis"[Title/Abstract] OR "dietary cirrhosis"[Title/Abstract] OR "hepatic cirrhosis"[Title/Abstract] OR "postnecrotic liver cirrhosis"[Title/Abstract] OR "liver cirrhosis"[Title/Abstract])) AND ("Liquid Biopsy"[MeSH Terms] OR ("Liquid Biopsy"[Title/Abstract] OR "liquid biops*"[Title/Abstract] OR "cfDNA"[Title/Abstract] OR "circulating cell-free DNA"[Title/Abstract] OR "circulating tumor DNA"[Title/Abstract] OR "circulating DNA"[Title/Abstract] OR "ctDNA"[Title/Abstract] OR "plasma DNA"[Title/Abstract] OR "serum DNA"[Title/Abstract] OR "blood DNA"[Title/Abstract] OR "circulating microRNA"[Title/Abstract] OR "microRNA"[Title/Abstract] OR "miRNA"[Title/Abstract] OR "miR"[Title/Abstract] OR "circulating tumor cell"[Title/Abstract] OR "CTC"[Title/Abstract] OR "exosome*"[Title/Abstract] OR "Exosomic"[Title/Abstract] OR "Exosomal"[Title/Abstract] OR "extracellular vesicle*"[Title/Abstract] OR "microvesicle*"[Title/Abstract] OR "alpha fetoprotein"[Title/Abstract] OR "AFP"[Title/Abstract])) AND ("carcinoma, hepatocellular"[MeSH Terms] OR ("liver cell carcinoma*"[Title/Abstract] OR "liver cell cancer"[Title/Abstract] OR "hepatocellular cancer*"[Title/Abstract] OR "hepatocellular carcinoma*"[Title/Abstract] OR "hepatocarcinoma"[Title/Abstract] OR "hepatic cell carcinoma"[Title/Abstract] OR "hepato-cellular carcinoma"[Title/Abstract] OR "hepatocyte carcinoma"[Title/Abstract])) AND ("diagnosis"[MeSH Terms] OR ("diagnosis"[Title/Abstract] OR "sensitivity"[Title/Abstract] OR "specificity"[Title/Abstract] OR "accuracy"[Title/Abstract] OR "receiver operating characteristic"[Title/Abstract]))) AND (2000:2024[pdat]) 3,511 10:11:19

11 (#1 OR #2 OR #3 OR #4) AND (#5 OR #6) AND (#7 OR #8) AND (#9 OR #10) ("hepatitis"[MeSH Terms] OR "hepatitis a"[MeSH Terms] OR ("hepatic inflammation"[Title/Abstract] OR "hepatic inflammatory disease"[Title/Abstract] OR "hepatitic disease"[Title/Abstract] OR "hepatitic illness"[Title/Abstract] OR "hepatitides"[Title/Abstract] OR "hepatocellular inflammation"[Title/Abstract] OR "liver infection"[Title/Abstract] OR "liver inflammation"[Title/Abstract] OR "liver inflammatory disease"[Title/Abstract] OR "subacute hepatitis"[Title/Abstract] OR "hepatitis"[Title/Abstract]) OR "liver cirrhosis"[MeSH Terms] OR ("fibrosis liver"[Title/Abstract] OR "Liver Fibrosis"[Title/Abstract] OR "cirrhosis"[Title/Abstract] OR "cirrhosis hepatis"[Title/Abstract] OR "cirrhosis liver"[Title/Abstract] OR "cryptogenic liver cirrhosis"[Title/Abstract] OR "dietary cirrhosis"[Title/Abstract] OR "hepatic cirrhosis"[Title/Abstract] OR "postnecrotic liver cirrhosis"[Title/Abstract] OR "liver cirrhosis"[Title/Abstract])) AND ("Liquid Biopsy"[MeSH Terms] OR ("Liquid Biopsy"[Title/Abstract] OR "liquid biops*"[Title/Abstract] OR "cfDNA"[Title/Abstract] OR "circulating cell-free DNA"[Title/Abstract] OR "circulating tumor DNA"[Title/Abstract] OR "circulating DNA"[Title/Abstract] OR "ctDNA"[Title/Abstract] OR "plasma DNA"[Title/Abstract] OR "serum DNA"[Title/Abstract] OR "blood DNA"[Title/Abstract] OR "circulating microRNA"[Title/Abstract] OR "microRNA"[Title/Abstract] OR "miRNA"[Title/Abstract] OR "miR"[Title/Abstract] OR "circulating tumor cell"[Title/Abstract] OR "CTC"[Title/Abstract] OR "exosome*"[Title/Abstract] OR "Exosomic"[Title/Abstract] OR "Exosomal"[Title/Abstract] OR "extracellular vesicle*"[Title/Abstract] OR "microvesicle*"[Title/Abstract] OR "alpha fetoprotein"[Title/Abstract] OR "AFP"[Title/Abstract])) AND ("carcinoma, hepatocellular"[MeSH Terms] OR ("liver cell carcinoma*"[Title/Abstract] OR "liver cell cancer"[Title/Abstract] OR "hepatocellular cancer*"[Title/Abstract] OR "hepatocellular carcinoma*"[Title/Abstract] OR "hepatocarcinoma"[Title/Abstract] OR "hepatic cell carcinoma"[Title/Abstract] OR "hepato-cellular carcinoma"[Title/Abstract] OR "hepatocyte carcinoma"[Title/Abstract])) AND ("diagnosis"[MeSH Terms] OR ("diagnosis"[Title/Abstract] OR "sensitivity"[Title/Abstract] OR "specificity"[Title/Abstract] OR "accuracy"[Title/Abstract] OR "receiver operating characteristic"[Title/Abstract])) 4,133 10:11:10

10 "diagnosis"[Title/Abstract] OR "sensitivity"[Title/Abstract] OR "specificity"[Title/Abstract] OR "accuracy"[Title/Abstract] OR "receiver operating characteristic"[Title/Abstract] "diagnosis"[Title/Abstract] OR "sensitivity"[Title/Abstract] OR "specificity"[Title/Abstract] OR "accuracy"[Title/Abstract] OR "receiver operating characteristic"[Title/Abstract] 3,503,178 10:10:27

9 diagnosis[MeSH Terms] "diagnosis"[MeSH Terms] 9,460,240 10:10:12

8 "liver cell carcinoma*"[Title/Abstract] OR "liver cell cancer"[Title/Abstract] OR "Hepatocellular Cancer*"[Title/Abstract] OR "hepatocellular carcinoma*"[Title/Abstract] OR "hepatocarcinoma"[Title/Abstract] OR "hepatic cell carcinoma"[Title/Abstract] OR "hepato-cellular carcinoma"[Title/Abstract] OR "hepatocyte carcinoma"[Title/Abstract] "liver cell carcinoma*"[Title/Abstract] OR "liver cell cancer"[Title/Abstract] OR "hepatocellular cancer*"[Title/Abstract] OR "hepatocellular carcinoma*"[Title/Abstract] OR "hepatocarcinoma"[Title/Abstract] OR "hepatic cell carcinoma"[Title/Abstract] OR "hepato-cellular carcinoma"[Title/Abstract] OR "hepatocyte carcinoma"[Title/Abstract] 128,879 10:09:58

7 liver cell carcinoma[MeSH Terms] "carcinoma, hepatocellular"[MeSH Terms] 107,165 10:09:39

6 "Liquid Biopsy"[Title/Abstract] OR "Liquid biops*"[Title/Abstract] OR "cfDNA"[Title/Abstract] OR "circulating cell-free DNA"[Title/Abstract] OR "circulating tumor DNA"[Title/Abstract] OR "circulating DNA"[Title/Abstract] OR "ctDNA"[Title/Abstract] OR "plasma DNA"[Title/Abstract] OR "serum DNA"[Title/Abstract] OR "blood DNA"[Title/Abstract] OR "circulating microRNA"[Title/Abstract] OR "microRNA"[Title/Abstract] OR "miRNA"[Title/Abstract] OR "miR"[Title/Abstract] OR "circulating tumor cell"[Title/Abstract] OR "CTC"[Title/Abstract] OR "Exosome*"[Title/Abstract] OR "Exosomic"[Title/Abstract] OR "Exosomal"[Title/Abstract] OR "Extracellular vesicle*"[Title/Abstract] OR "Microvesicle*"[Title/Abstract] OR "alpha fetoprotein"[Title/Abstract] OR "AFP"[Title/Abstract] "Liquid Biopsy"[Title/Abstract] OR "liquid biops*"[Title/Abstract] OR "cfDNA"[Title/Abstract] OR "circulating cell-free DNA"[Title/Abstract] OR "circulating tumor DNA"[Title/Abstract] OR "circulating DNA"[Title/Abstract] OR "ctDNA"[Title/Abstract] OR "plasma DNA"[Title/Abstract] OR "serum DNA"[Title/Abstract] OR "blood DNA"[Title/Abstract] OR "circulating microRNA"[Title/Abstract] OR "microRNA"[Title/Abstract] OR "miRNA"[Title/Abstract] OR "miR"[Title/Abstract] OR "circulating tumor cell"[Title/Abstract] OR "CTC"[Title/Abstract] OR "exosome*"[Title/Abstract] OR "Exosomic"[Title/Abstract] OR "Exosomal"[Title/Abstract] OR "extracellular vesicle*"[Title/Abstract] OR "microvesicle*"[Title/Abstract] OR "alpha fetoprotein"[Title/Abstract] OR "AFP"[Title/Abstract] 259,294 10:09:24

5 Liquid Biopsy[MeSH Terms] "liquid biopsy"[MeSH Terms] 3,331 10:09:08

4 "Fibrosis, Liver"[Title/Abstract] OR "Liver Fibrosis"[Title/Abstract] OR "cirrhosis"[Title/Abstract] OR "cirrhosis hepatis"[Title/Abstract] OR "cirrhosis, liver"[Title/Abstract] OR "cryptogenic liver cirrhosis"[Title/Abstract] OR "dietary cirrhosis"[Title/Abstract] OR "dietary liver cirrhosis"[Title/Abstract] OR "hepatic cirrhosis"[Title/Abstract] OR "postnecrotic liver cirrhosis"[Title/Abstract] OR "liver cirrhosis"[Title/Abstract] "fibrosis liver"[Title/Abstract] OR "Liver Fibrosis"[Title/Abstract] OR "cirrhosis"[Title/Abstract] OR "cirrhosis hepatis"[Title/Abstract] OR "cirrhosis liver"[Title/Abstract] OR "cryptogenic liver cirrhosis"[Title/Abstract] OR "dietary cirrhosis"[Title/Abstract] OR "hepatic cirrhosis"[Title/Abstract] OR "postnecrotic liver cirrhosis"[Title/Abstract] OR "liver cirrhosis"[Title/Abstract] 128,318 10:08:45

3 Liver Cirrhosis[MeSH Terms] "liver cirrhosis"[MeSH Terms] 103,951 10:08:28

2 "hepatic cellular inflammation"[Title/Abstract] OR "hepatic inflammation"[Title/Abstract] OR "hepatic inflammatory disease"[Title/Abstract] OR "hepatitic disease"[Title/Abstract] OR "hepatitic disorder"[Title/Abstract] OR "hepatitic illness"[Title/Abstract] OR "hepatitides"[Title/Abstract] OR "hepatocellular inflammation"[Title/Abstract] OR "hepatocellular inflammatory disease"[Title/Abstract] OR "hepatocellular inflammatory syndrome"[Title/Abstract] OR "hepatoinflammation"[Title/Abstract] OR "liver infection"[Title/Abstract] OR "liver inflammation"[Title/Abstract] OR "liver inflammatory disease"[Title/Abstract] OR "subacute hepatitis"[Title/Abstract] OR "hepatitis"[Title/Abstract] "hepatic inflammation"[Title/Abstract] OR "hepatic inflammatory disease"[Title/Abstract] OR "hepatitic disease"[Title/Abstract] OR "hepatitic illness"[Title/Abstract] OR "hepatitides"[Title/Abstract] OR "hepatocellular inflammation"[Title/Abstract] OR "liver infection"[Title/Abstract] OR "liver inflammation"[Title/Abstract] OR "liver inflammatory disease"[Title/Abstract] OR "subacute hepatitis"[Title/Abstract] OR "hepatitis"[Title/Abstract] 255,002 10:08:11

1 Hepatitis[MeSH Terms] "hepatitis"[MeSH Terms] OR "hepatitis a"[MeSH Terms] 184,632 10:07:49

**WOS：**

(TI=(("hepatic cellular inflammation") OR ("hepatic inflammation") OR ("hepatic inflammatory disease") OR ("hepatitic disease") OR ("hepatitic disorder") OR ("hepatitic illness") OR ("hepatitides") OR ("hepatocellular inflammation") OR ("hepatocellular inflammatory disease") OR ("hepatocellular inflammatory syndrome") OR ("hepatoinflammation") OR ("liver infection") OR ("liver inflammation") OR ("liver inflammatory disease") OR ("subacute hepatitis") OR ("hepatitis") OR ("Fibrosis, Liver") OR ("Liver Fibrosis") OR ("cirrhosis") OR ("cirrhosis hepatis") OR ("cirrhosis, liver") OR ("cryptogenic liver cirrhosis") OR ("dietary cirrhosis") OR ("dietary liver cirrhosis") OR ("hepatic cirrhosis") OR ("postnecrotic liver cirrhosis") OR ("liver cirrhosis")

) OR AB=(("hepatic cellular inflammation") OR ("hepatic inflammation") OR ("hepatic inflammatory disease") OR ("hepatitic disease") OR ("hepatitic disorder") OR ("hepatitic illness") OR ("hepatitides") OR ("hepatocellular inflammation") OR ("hepatocellular inflammatory disease") OR ("hepatocellular inflammatory syndrome") OR ("hepatoinflammation") OR ("liver infection") OR ("liver inflammation") OR ("liver inflammatory disease") OR ("subacute hepatitis") OR ("hepatitis") OR ("Fibrosis, Liver") OR ("Liver Fibrosis") OR ("cirrhosis") OR ("cirrhosis hepatis") OR ("cirrhosis, liver") OR ("cryptogenic liver cirrhosis") OR ("dietary cirrhosis") OR ("dietary liver cirrhosis") OR ("hepatic cirrhosis") OR ("postnecrotic liver cirrhosis") OR ("liver cirrhosis")

) OR AK=(("hepatic cellular inflammation") OR ("hepatic inflammation") OR ("hepatic inflammatory disease") OR ("hepatitic disease") OR ("hepatitic disorder") OR ("hepatitic illness") OR ("hepatitides") OR ("hepatocellular inflammation") OR ("hepatocellular inflammatory disease") OR ("hepatocellular inflammatory syndrome") OR ("hepatoinflammation") OR ("liver infection") OR ("liver inflammation") OR ("liver inflammatory disease") OR ("subacute hepatitis") OR ("hepatitis") OR ("Fibrosis, Liver") OR ("Liver Fibrosis") OR ("cirrhosis") OR ("cirrhosis hepatis") OR ("cirrhosis, liver") OR ("cryptogenic liver cirrhosis") OR ("dietary cirrhosis") OR ("dietary liver cirrhosis") OR ("hepatic cirrhosis") OR ("postnecrotic liver cirrhosis") OR ("liver cirrhosis")

)) AND (TI=(("Liquid Biopsy") OR ("Liquid biops*") OR ("cfDNA") OR ("circulating cell-free DNA") OR ("circulating tumor DNA") OR ("circulating DNA") OR ("ctDNA") OR ("plasma DNA") OR ("serum DNA") OR ("blood DNA") OR ("circulating microRNA") OR ("microRNA") OR ("miRNA") OR ("miR") OR ("circulating tumor cell") OR ("CTC") OR ("Exosome*") OR ("Exosomic") OR ("Exosomal") OR ("Extracellular vesicle*") OR ("Microvesicle*") OR ("alpha fetoprotein") OR ("AFP")

) OR AB=(("Liquid Biopsy") OR ("Liquid biops*") OR ("cfDNA") OR ("circulating cell-free DNA") OR ("circulating tumor DNA") OR ("circulating DNA") OR ("ctDNA") OR ("plasma DNA") OR ("serum DNA") OR ("blood DNA") OR ("circulating microRNA") OR ("microRNA") OR ("miRNA") OR ("miR") OR ("circulating tumor cell") OR ("CTC") OR ("Exosome*") OR ("Exosomic") OR ("Exosomal") OR ("Extracellular vesicle*") OR ("Microvesicle*") OR ("alpha fetoprotein") OR ("AFP")

) OR AK=(("Liquid Biopsy") OR ("Liquid biops*") OR ("cfDNA") OR ("circulating cell-free DNA") OR ("circulating tumor DNA") OR ("circulating DNA") OR ("ctDNA") OR ("plasma DNA") OR ("serum DNA") OR ("blood DNA") OR ("circulating microRNA") OR ("microRNA") OR ("miRNA") OR ("miR") OR ("circulating tumor cell") OR ("CTC") OR ("Exosome*") OR ("Exosomic") OR ("Exosomal") OR ("Extracellular vesicle*") OR ("Microvesicle*") OR ("alpha fetoprotein") OR ("AFP")

)) AND (TI=(("liver cell carcinoma*") OR ("liver cell cancer") OR ("Hepatocellular Cancer*") OR ("hepatocellular carcinoma*") OR ("hepatocarcinoma") OR ("hepatic cell carcinoma") OR ("hepato-cellular carcinoma") OR ("hepatocyte carcinoma")

) OR AB=(("liver cell carcinoma*") OR ("liver cell cancer") OR ("Hepatocellular Cancer*") OR ("hepatocellular carcinoma*") OR ("hepatocarcinoma") OR ("hepatic cell carcinoma") OR ("hepato-cellular carcinoma") OR ("hepatocyte carcinoma")

) OR AK=(("liver cell carcinoma*") OR ("liver cell cancer") OR ("Hepatocellular Cancer*") OR ("hepatocellular carcinoma*") OR ("hepatocarcinoma") OR ("hepatic cell carcinoma") OR ("hepato-cellular carcinoma") OR ("hepatocyte carcinoma")

)) AND (TI=(("diagnosis") OR ("sensitivity") OR ("specificity") OR ("accuracy") OR ("receiver operating characteristic")

) OR AB=(("diagnosis") OR ("sensitivity") OR ("specificity") OR ("accuracy") OR ("receiver operating characteristic")

) OR AK=(("diagnosis") OR ("sensitivity") OR ("specificity") OR ("accuracy") OR ("receiver operating characteristic")

))
