# Supplementary material for: Diagnosis of hepatocellular carcinoma using liquid biopsy-based biomarkers: a systematic review and network meta-analysis
Source: Front Oncol. 2025 Jan 28;14:1483521. doi: 10.3389/fonc.2024.1483521 (PMC11810725; doi:10.3389/fonc.2024.1483521)
Supplement: Supplementary file 3 [file Table2.docx]

**Table S2** Inclusion of 82 studies

1. Chen, Y., et al., *Serum miR-96 is a promising biomarker for hepatocellular carcinoma in patients with chronic hepatitis B virus infection.* Int J Clin Exp Med, 2015. **8**(10): p. 18462-8.

2. Guo, S., et al., *Circular RNA 0006602 in plasma exosomes: a new potential diagnostic biomarker for hepatocellular carcinoma.* Am J Transl Res, 2021. **13**(6): p. 6001-6015.

3. Yosry, A., et al., *Highly Sensitive Serum miRNA Panel for the Diagnosis of Hepatocellular Carcinoma in Egyptian Patients with HCV-Related HCC.* Lab Med, 2022. **53**(5): p. 523-529.

4. Hung, C.H., et al., *Circulating microRNAs as biomarkers for diagnosis of early hepatocellular carcinoma associated with hepatitis B virus.* Int J Cancer, 2016. **138**(3): p. 714-20.

5. Abd El Gwad, A., et al., *Role of exosomal competing endogenous RNA in patients with hepatocellular carcinoma.* J Cell Biochem, 2018. **119**(10): p. 8600-8610.

6. Matboli, M., et al., *circRNAs (hsa_circ_00156, hsa_circ _000224, and hsa_circ _000520) are novel potential biomarkers in hepatocellular carcinoma.* J Cell Biochem, 2019. **120**(5): p. 7711-7724.

7. Li, L., et al., *Serum miR-18a: a potential marker for hepatitis B virus-related hepatocellular carcinoma screening.* Dig Dis Sci, 2012. **57**(11): p. 2910-6.

8. Rashad, N.M., et al., *Serum miRNA-27a and miRNA-18b as potential predictive biomarkers of hepatitis C virus-associated hepatocellular carcinoma.* Mol Cell Biochem, 2018. **447**(1-2): p. 125-136.

9. Habieb, A., et al., *Potential role of lncRNA-TSIX, miR-548-a-3p, and SOGA1 mRNA in the diagnosis of hepatocellular carcinoma.* Mol Biol Rep, 2019. **46**(4): p. 4581-4590.

10. El-Hamouly, M.S., et al., *Circulating microRNA-301 as a promising diagnostic biomarker of hepatitis C virus-related hepatocellular carcinoma.* Mol Biol Rep, 2019. **46**(6): p. 5759-5765.

11. Elhendawy, M., et al., *MicroRNA signature in hepatocellular carcinoma patients: identification of potential markers.* Mol Biol Rep, 2020. **47**(7): p. 4945-4953.

12. Piciocchi, M., et al., *Circulating free DNA in the progression of liver damage to hepatocellular carcinoma.* Hepatol Int, 2013. **7**(4): p. 1050-7.

13. Yang, L., et al., *Expression of serum miR-218 in hepatocellular carcinoma and its prognostic significance.* Clin Transl Oncol, 2016. **18**(8): p. 841-7.

14. Huang, Z., et al., *Quantitation of plasma circulating DNA using quantitative PCR for the detection of hepatocellular carcinoma.* Pathol Oncol Res, 2012. **18**(2): p. 271-6.

15. Chen, L., et al., *Serum miR-182 and miR-331-3p as diagnostic and prognostic markers in patients with hepatocellular carcinoma.* Tumour Biol, 2015. **36**(10): p. 7439-47.

16. Zuo, D., et al., *Combination of miR-125b and miR-27a enhances sensitivity and specificity of AFP-based diagnosis of hepatocellular carcinoma.* Tumour Biol, 2016. **37**(5): p. 6539-49.

17. Nasser, M.Z., et al., *Circulating microRNAs (miR-21, miR-223, miR-885-5p) along the clinical spectrum of HCV-related chronic liver disease in Egyptian patients.* Arab J Gastroenterol, 2019. **20**(4): p. 198-204.

18. Fouda, M.S., et al., *Development of a novel panel based on micro-RNAs (21, 29a, 200 and 335) and alpha-fetoprotein as diagnostic biomarkers for hepatocellular carcinoma associated with hepatitis C infection.* Arab J Gastroenterol, 2021. **22**(1): p. 28-33.

19. Li, Z., et al., *Using circular RNA SMARCA5 as a potential novel biomarker for hepatocellular carcinoma.* Clin Chim Acta, 2019. **492**: p. 37-44.

20. Abdelgawad, I.A., N.H. Radwan, and H.R. Hassanein, *KIAA0101 mRNA expression in the peripheral blood of hepatocellular carcinoma patients: Association with some clinicopathological features.* Clin Biochem, 2016. **49**(10-11): p. 787-91.

21. Zhuang, C., et al., *Serum miR-21, miR-26a and miR-101 as potential biomarkers of hepatocellular carcinoma.* Clin Res Hepatol Gastroenterol, 2016. **40**(4): p. 386-96.

22. Lin, L., et al., *Serum miR-224 as a biomarker for detection of hepatocellular carcinoma at early stage.* Clin Res Hepatol Gastroenterol, 2016. **40**(4): p. 397-404.

23. Han, J., et al., *Identification of plasma miR-148a as a noninvasive biomarker for hepatocellular carcinoma.* Clin Res Hepatol Gastroenterol, 2019. **43**(5): p. 585-593.

24. Sun, Q., et al., *Evaluation of miR-331-3p and miR-23b-3p as serum biomarkers for hepatitis c virus-related hepatocellular carcinoma at early stage.* Clin Res Hepatol Gastroenterol, 2020. **44**(1): p. 21-28.

25. Amr, K.S., et al., *Early diagnostic evaluation of miR-122 and miR-224 as biomarkers for hepatocellular carcinoma.* Genes Dis, 2017. **4**(4): p. 215-221.

26. Yan, L., et al., *Diagnostic value of circulating cell-free DNA levels for hepatocellular carcinoma.* Int J Infect Dis, 2018. **67**: p. 92-97.

27. Tomimaru, Y., et al., *Circulating microRNA-21 as a novel biomarker for hepatocellular carcinoma.* J Hepatol, 2012. **56**(1): p. 167-75.

28. Shaheen, N.M.H., et al., *Role of circulating miR-182 and miR-150 as biomarkers for cirrhosis and hepatocellular carcinoma post HCV infection in Egyptian patients.* Virus Res, 2018. **255**: p. 77-84.

29. Luo, P., et al., *Identification of long non-coding RNA ZFAS1 as a novel biomarker for diagnosis of HCC.* Biosci Rep, 2018. **38**(4).

30. Li, X., et al., *Clinical significance of serum miR-487b in HBV-related hepatocellular carcinoma and its potential mechanism.* Infect Dis (Lond), 2021. **53**(7): p. 546-554.

31. AA, A.L., et al., *Utility of a microRNA panel in diagnosis and prognosis of hepatitis C-associated hepatocellular carcinoma.* Lab Med, 2024. **55**(3): p. 310-319.

32. Zhao, L., Q. Yang, and J. Liu, *Clinical Value Evaluation of microRNA-324-3p and Other Available Biomarkers in Patients With HBV Infection-Related Hepatocellular Carcinoma.* Open Forum Infect Dis, 2021. **8**(6): p. ofab108.

33. Chen, S., et al., *Differential expression of plasma microRNA-125b in hepatitis B virus-related liver diseases and diagnostic potential for hepatitis B virus-induced hepatocellular carcinoma.* Hepatol Res, 2017. **47**(4): p. 312-320.

34. Huang, J., et al., *A Circulating Long Noncoding RNA Panel Serves as a Diagnostic Marker for Hepatocellular Carcinoma.* Dis Markers, 2020. **2020**: p. 5417598.

35. Miura, N., et al., *Serum human telomerase reverse transcriptase messenger RNA as a novel tumor marker for hepatocellular carcinoma.* Clin Cancer Res, 2005. **11**(9): p. 3205-9.

36. Shen, X., et al., *Dysregulation of serum microRNA-574-3p and its clinical significance in hepatocellular carcinoma.* Ann Clin Biochem, 2018. **55**(4): p. 478-484.

37. Nguyen, H.B., et al., *Diagnostic Value of hTERT mRNA and in Combination With AFP, AFP-L3%, Des-γ-carboxyprothrombin for Screening of Hepatocellular Carcinoma in Liver Cirrhosis Patients HBV or HCV-Related.* Cancer Inform, 2022. **21**: p. 11769351221100730.

38. Miura, N., et al., *A novel biomarker TERTmRNA is applicable for early detection of hepatoma.* BMC Gastroenterol, 2010. **10**: p. 46.

39. Zhang, Z.Q., et al., *Serum microRNA 143 and microRNA 215 as potential biomarkers for the diagnosis of chronic hepatitis and hepatocellular carcinoma.* Diagn Pathol, 2014. **9**: p. 135.

40. Han, Y., et al., *Serum long non-coding RNA SCARNA10 serves as a potential diagnostic biomarker for hepatocellular carcinoma.* BMC Cancer, 2022. **22**(1): p. 431.

41. Yu, F., et al., *microRNA-150: a promising novel biomarker for hepatitis B virus-related hepatocellular carcinoma.* Diagn Pathol, 2015. **10**: p. 129.

42. Dhayat, S.A., et al., *Circulating microRNA-200 Family as Diagnostic Marker in Hepatocellular Carcinoma.* PLoS One, 2015. **10**(10): p. e0140066.

43. Li, J., et al., *Improving the Detection of Hepatocellular Carcinoma Using Serum AFP Expression in Combination with GPC3 and Micro-RNA MiR-122 Expression.* Open Life Sci, 2019. **14**: p. 53-61.

44. Gharib, A.F., et al., *Value of Serum miRNA-96-5p and miRNA-99a-5p as Diagnostic Biomarkers for Hepatocellular Carcinoma.* Int J Gen Med, 2022. **15**: p. 2427-2436.

45. Qiao, G.L., et al., *Hsa_circ_0003998 may be used as a new biomarker for the diagnosis and prognosis of hepatocellular carcinoma.* Onco Targets Ther, 2019. **12**: p. 5849-5860.

46. Quoc, N.B., et al., *Expression of Plasma hsa-miR122 in HBV-Related Hepatocellular Carcinoma (HCC) in Vietnamese Patients.* Microrna, 2018. **7**(2): p. 92-99.

47. Wang, Y., et al., *Down-regulation of long non-coding RNA GAS5-AS1 and its prognostic and diagnostic significance in hepatocellular carcinoma.* Cancer Biomark, 2018. **22**(2): p. 227-236.

48. Wang, Z., et al., *Long noncoding RNA MyD88 functions as a promising diagnostic biomarker in hepatocellular carcinoma.* Front Endocrinol (Lausanne), 2023. **14**: p. 938102.

49. Yousuf, T., et al., *Diagnostic implication of a circulating serum-based three-microRNA signature in hepatocellular carcinoma.* Front Genet, 2022. **13**: p. 929787.

50. Elfert, A.Y., et al., *Implication of miR-122, miR-483, and miR-335 Expression Levels as Potential Signatures in HCV-Related Hepatocellular Carcinoma (HCC) in Egyptian Patients.* Front Mol Biosci, 2022. **9**: p. 864839.

51. Eldosoky, M.A., et al., *Diagnostic Significance of hsa-miR-21-5p, hsa-miR-192-5p, hsa-miR-155-5p, hsa-miR-199a-5p Panel and Ratios in Hepatocellular Carcinoma on Top of Liver Cirrhosis in HCV-Infected Patients.* Int J Mol Sci, 2023. **24**(4).

52. Boonkaew, B., et al., *Circulating Extracellular Vesicle-Derived microRNAs as Novel Diagnostic and Prognostic Biomarkers for Non-Viral-Related Hepatocellular Carcinoma.* Int J Mol Sci, 2023. **24**(22).

53. Gao, S., et al., *Diagnostic utility of plasma lncRNA small nucleolar RNA host gene 1 in patients with hepatocellular carcinoma.* Mol Med Rep, 2018. **18**(3): p. 3305-3313.

54. Zhao, Q., et al., *Expression of the microRNA-143/145 cluster is decreased in hepatitis B virus-associated hepatocellular carcinoma and may serve as a biomarker for tumorigenesis in patients with chronic hepatitis B.* Oncol Lett, 2018. **15**(5): p. 6115-6122.

55. Li, T., et al., *Downregulation of microRNA-139 is associated with hepatocellular carcinoma risk and short-term survival.* Oncol Rep, 2014. **31**(4): p. 1699-706.

56. Xie, Y., et al., *Expression profiling of serum microRNA-101 in HBV-associated chronic hepatitis, liver cirrhosis, and hepatocellular carcinoma.* Cancer Biol Ther, 2014. **15**(9): p. 1248-55.

57. El-Garem, H., et al., *Circulating microRNA, miR-122 and miR-221 signature in Egyptian patients with chronic hepatitis C related hepatocellular carcinoma.* World J Hepatol, 2014. **6**(11): p. 818-24.

58. Wahb, A., et al., *Circulating microRNA 9-3p and serum endocan as potential biomarkers for hepatitis C virus-related hepatocellular carcinoma.* World J Hepatol, 2021. **13**(11): p. 1753-1765.

59. Youssef, S.S., et al., *Assessment of circulating levels of microRNA-326, microRNA-424, and microRNA-511 as biomarkers for hepatocellular carcinoma in Egyptians.* World J Hepatol, 2022. **14**(8): p. 1562-1575.

60. Nomair, A.M., et al., *The clinical significance of serum miRNA-224 expression in hepatocellular carcinoma.* Clin Exp Hepatol, 2020. **6**(1): p. 20-27.

61. Moshiri, F., et al., *Circulating miR-106b-3p, miR-101-3p and miR-1246 as diagnostic biomarkers of hepatocellular carcinoma.* Oncotarget, 2018. **9**(20): p. 15350-15364.

62. Lou, Z.H., et al., *Diagnostic Potential of the Serum lncRNAs HOTAIR, BRM and ICR for Hepatocellular Carcinoma.* Front Biosci (Landmark Ed), 2022. **27**(9): p. 264.

63. Farag, R.M.A., et al., *Studying the Impact of Golgi Protein 73 Serving as a Candidate Biomarker in Early Diagnosis for Hepatocellular Carcinoma among Saudi Patients.* Asian Pac J Cancer Prev, 2019. **20**(1): p. 215-220.

64. Shehab-Eldeen, S., et al., *Diagnostic Performance of microRNA-122 and microRNA-224 in Hepatitis C Virus-Induced Hepatocellular Carcinoma (HCC).* Asian Pac J Cancer Prev, 2019. **20**(8): p. 2515-2522.

65. Aboelwafa, R.A., et al., *The expression of microRNA-331-3p and microRNA-23b3 in Egyptian patients with early-stage hepatocellular carcinoma in hepatitis C-related liver cirrhosis.* Egyptian Liver Journal, 2021. **11**(1): p. 49.

66. Cimentepe, M., et al., *THE INVESTIGATION OF MICRORNAS AS POTENTIAL BIOMARKERS IN SERUM AND TISSUE FOR HEPATITIS B VIRUS RELATED HEPATOCELLULAR CARCINOMA.* Acta Medica Mediterranea, 2021. **37**: p. 1335.

67. M. A.. Farag, R., et al., *Influence of Glypican-3 as Anewly Diagnostic Biomarker in Earlydetection of Hepatocellular Carcinoma among Saudi Patients.* Biomedical and Pharmacology Journal, 2018. **11**: p. 1789-1796.

68. Gibriel, A.A., et al., *Diagnosis and staging of HCV associated fibrosis, cirrhosis and hepatocellular carcinoma with target identification for miR-650, 552-3p, 676-3p, 512-5p and 147b.* Cancer Biomark, 2022. **34**(3): p. 413-430.

69. Hussein, M.A., et al., *MicroRNA 21as a novel biomarker in hepatitis C virus-related hepatocellular carcinoma.* The Egyptian Journal of Internal Medicine, 2022. **34**(1): p. 56.

70. Shehab-Eldeen, S., et al., *Corrigendum to 'MicroRNA-29a and MicroRNA-124 as novel biomarkers for hepatocellular carcinoma' [Digestive and Liver Disease Volume 55, Issue 2, February 2023, Pages 283-290].* Dig Liver Dis, 2023. **55**(8): p. 1164.

71. Xu, L.-j., et al. *Association of serum microRNA-125 b and HBV-related hepatocellular carcinoma in Chinese Han patients*. 2018.

72. Duo, Z., et al., *The diagnosis value of combined detection of serum mir-125b and alpha-fetoprotein for primary hepatocellular carcinoma.* Chinese Journal of Clinical Oncology, 2014(10): p. 662-666.

73. Wenyuan, S., et al., *Significance of serum lncRNA-PVT1 expression in diagnosis and prognosis of hepatocellular carcinoma.* Journal of Practical Oncology, 2020. **35**(4): p. 317-321.

74. Lyu, L., et al., *The diagnostic value of plasma exosomal hsa_circ_0070396 for hepatocellular carcinoma.* Biomark Med, 2021. **15**(5): p. 359-371.

75. Wang, Y., et al., *Serum exosomal microRNAs combined with alpha-fetoprotein as diagnostic markers of hepatocellular carcinoma.* Cancer Med, 2018. **7**(5): p. 1670-1679.

76. Wang, Y., et al., *The Potential of Serum Exosomal hsa_circ_0028861 as the Novel Diagnostic Biomarker of HBV-Derived Hepatocellular Cancer.* Front Genet, 2021. **12**: p. 703205.

77. Wei, Y., et al., *Serum exosomal microRNA-370-3p and microRNA-196a-5p are potential biomarkers for the diagnosis and prognosis of hepatocellular carcinoma.* Folia Histochem Cytobiol, 2022. **60**(3): p. 215-225.

78. Chen, S., et al., *Serum exosomal miR-34a as a potential biomarker for the diagnosis and prognostic of hepatocellular carcinoma.* J Cancer, 2022. **13**(5): p. 1410-1417.

79. Ghosh, S., et al., *The exosome encapsulated microRNAs as circulating diagnostic marker for hepatocellular carcinoma with low alpha-fetoprotein.* Int J Cancer, 2020. **147**(10): p. 2934-2947.

80. Xu, H., et al., *Serum Exosomal Long Noncoding RNAs ENSG00000258332.1 and LINC00635 for the Diagnosis and Prognosis of Hepatocellular Carcinoma.* Cancer Epidemiol Biomarkers Prev, 2018. **27**(6): p. 710-716.

81. Xu, H., et al., *Serum exosomal hnRNPH1 mRNA as a novel marker for hepatocellular carcinoma.* Clin Chem Lab Med, 2018. **56**(3): p. 479-484.

82. Yang, J., et al., *Exosomal microRNA panel as a diagnostic biomarker in patients with hepatocellular carcinoma.* Front Cell Dev Biol, 2022. **10**: p. 927251.
