# Supplementary material for: Diagnosis of hepatocellular carcinoma using liquid biopsy-based biomarkers: a systematic review and network meta-analysis
Source: Front Oncol. 2025 Jan 28;14:1483521. doi: 10.3389/fonc.2024.1483521 (PMC11810725; doi:10.3389/fonc.2024.1483521)
Supplement: Supplementary file 4 [file Table3.docx]

**Table S3 PCR assay method of 82 studies**

| **No.** | **First Author** | **Publication Year** | **Detection Method** | **sample** | **Detection process** |
| --- | --- | --- | --- | --- | --- |
| 1 | Chen | 2015 | RT-PCR | Serum | Complement DNA was synthesized using the  miRCURY LNA™ Universal RT miRNA PCR kit  (Exiqon, Vedbaek, Denmark). Reverse transcriptase products were used as templates for the next PCR process after a 1:5 dilution and  were detected in 10 μL PCR reactions according to the protocol for miRCURY LNA™ Universal RT miRNA PCR kit. All amplifications were assayed with SYBR Green on an ABI PRISM 7500 sequence detection system (Applied Biosystems, Foster City, California,  USA) under the following cycling conditions:95℃ for 1 min, followed by 45 cycles of 95℃ for 10 s and 60℃ for 1 min. The  relative expression levels of miR-96 were calculated by the2^-△△Ct^ method, as previously described . MiR-16 was used as the endogenous control to normalize the data. |
| 2 | Guo | 2021 | qPCR | plasma | ExoRNEasy kit (Qiagen) was used to isolate  total RNA from exosomes. the total RNA was quantified then transcribed to cDNA. qPCR was used to detect the level of  Exo-circRNA from the cDNA. Glyceraldehyde-3-phosphate dehydrogenase (GAPDH) was used as the endogenous control gene,  2^-△△Ct^ was used to indicate the relative expression. |
| 3 | Yosry | 2022 | RT-PCR | Serum | The miRNeasy Mini Kit was used to extract miRNA and total RNA following the manufacturer’s protocols. SYBR Green-Based RT-PCR Analysis. According to the manufacturer’s instructions, the cDNA buffered with miScript HiSpec Buffer was prepared from 60 ng purified  miRNA using the miScript II RT Kit (SABiosciences). The cDNA was  then diluted 1:5 in nuclease-free water. The quantitative RT-PCR was  conducted using miScript miRNA PCR master mix (SABiosciences). The SNORD6 miRNA was selected as a reference gene for normalization. .Amplification reactions were done under the following conditions: initial incubation for 15 min at 95℃ (preactivation HotStarTaq), accompanied by 40 cycles at 94℃ for 15s, 55℃ for 30s, and 70℃for 30s. Amplification was carried out using the real-time Rotor-Gene PCR (Qiagen). Relative expression of each gene was estimated using the 2^-△△Ct^ method, |
| 4 | Hung | 2015 | qRT-PCR | serum | MiRNA expressions were quantified in real-time quantitative reverse transcription polymerase chain reaction (RT-PCR) systems using TaqManVR microRNA assays according to the manufacturer’s protocols  (Applied Biosystems), which included two steps: RT reaction  and TaqMan real-time PCR assay. RT reactions were performed with 10 ng of total RNA, 50 nM stem-loop miRNA-specific RT primers, 1×RT buffer, 0.25 mM of dNTPs, 3.33 U/μL MultiScribe RTase and 0.25 U/μL RNase inhibitor. RT products were subjected to miRNA expression assay for real-time quantitative PCR in a 20-μL final volume containing 2μL of RT product, 1μL of 20×TaqMan microRNA Assay (Applied Biosystems) and 10μL of 2×TaqMan Universal PCR Master Mix (Applied Biosystems). The PCR cycling parameters were 95℃for 15 sec followed by 60℃ for 30 sec for 40 cycles. U6nRNA TaqMan  miRNA assay (Applied Biosystems) was used as endogenous control for miRNA expression analysis. Real-time quantitative PCR was performed in a 7500 Fast Real-Time System (Applied Biosystems). The expression of miRNA relative to small RNA U6 was reported as dCT (△CT), which was calculated by subtracting the Ct of U6 RNA from the Ct of target miRNA. |
| 5 | Gwad | 2018 | qRT-PCR | serum | The levels of exosomal RAB11A mRNA and exosomal  lncRNA-RP11-513I15.6 in sera were measured using aQuantitect SYBR Green Master Mix Kit and an RT^2^ SYBR Green ROX qPCR Mastermix (Qiagen), respectively, using a Rotor Gene real-time PCR detection system (Qiagen, Germany) and specific primers (Accession:NM_001206836 and ENST00000429998, respectively)  supplied by Qiagen. Beta-actin (Accession NM_001101) was used as a housekeeping gene. The exosomal miR-1262 expression in sera was investigated by a miScript SYBR Green kit (Qiagen), a miScript universal primer and a miRNA‐specific forward primer (hsa‐mir‐1262 miScript Primer Assay), (Accession: NR_031664). All steps followed the manufacture’s suggested protocol, and RNU‐6 was used as an internal control. All the PCR primers were obtained from Qiagen.  The PCR program for SYBR Green‐based qPCR was as follows: first, denaturation at 95°C for 15 minutes; then 40 cycles of denaturation for 10 seconds at 94°C; after that, annealing for 30 seconds at 55°C; and finally, extension for 34 seconds at 70°C. Each reaction was performed in duplicate. A rotor gene manual was used to calculate the threshold cycle (Ct) value of each sample. Ct values more than 36 were considered negative. The specificities of the amplicons for the SYBR Green‐based  PCR amplification were affirmed by the melting curves.  The 2^-△△Ct^ technique was used to measure the expression of the exosomal RNA‐based biomarker panel. Housekeeping genes were used as an invariant control to normalize the raw data of the samples and compare these results with a reference sample. |
| 6 | Matboli | 2018 | Real‐time PCR | serum | cDNA synthesis was performed using the RT2 miRNA First Strand Kit (Qiagen, Valencia, CA) using 1 µg RNA.Real-time PCR was performed using a StepOnePlus. Applied Biosystem thermocycler. All reactions were performed in triplicates. the expression of hsa-circ-00156, hsa_circ_000224, and hsa_circ_000520 in serum samples was assessed using the QuantiTect SYBR Green PCR Kit (Qiagen) and gene‐specific divergent primers (Circular RNA specific QuantiTect Primers), on  StepOnePlus System (Applied Biosystems Inc, Foster, CA). Beta-actin was used as the endogenous control gene. The divergent primers were designed by the Circinteractome database (available at <http://circinteractome.nia.>nih.gov/) and synthesized by (Qiagen, Valencia, CA). The PCR program for SYBR-green-based real-time  PCR was as follows: initial denaturation at 95°C for  15 minutes, followed by 40 cycles of denaturation for  10 seconds at 94°C, annealing for 30 seconds at 55°C,  and, finally, extension for 34 seconds at 70°C. All reactions were carried out in triplicates. The threshold cycle (Ct) value of each sample was calculated using StepOnePlus software v2.2.2 (Applied Biosystems Inc) with any value greater than 36 considered negative. The relative quantification of circRNA expression was calculated according to the2^-△△Ct^ method. |
| 7 | Li | 2012 | qPCR | serum | The expression levels of miRNA were confirmed with real-time qPCR using individual miRNA-specific primers and miScript SYBR Green PCR kit (Qiagen, Germanny) on the ABI 9700 HT Real-time PCR system  (Applied Biosystems). Each amplification reaction was performed in a final volume of 20μL containing 2μL of the cDNA (100 ng/reaction), 10μL 2× SYBR Green PCR Master mix, 2μL10×9 miScript Universal Primer, 2μL10× miScript Primer Assay, and 4μL RNase-free water. Reaction was performed at 95℃ for 10 min and in 40  cycles at 95℃for 15 s, 55℃ for 30 s, and 70℃for 30 s. At the end of the PCR cycles, melting curve analyses was performed to verify the specificity and identity of PCR products. Each sample was run in duplicates for analysis. The miRNA relative expression levels were  calculated using the cycle threshold (Ct) values and evaluated by the  2^-△△Ct^ method. |
| 8 | Rashad | 2017 | qRT‑PCR | serum | One microgram miRNA was used in reverse transcription  with a miScript II RT Kit (Qiagen/SABiosciences Corporation, Frederick, MD, USA). Then, the cDNA was kept in −80℃ till the real-time-polymerase chain reaction (RT-PCR) analyses.  Quantitative real-time-polymerase chain reaction  (qRT-PCR) was carried out by StepOnePlus™ System  (Applied Biosystems Inc., Foster, CA, USA). Small RNA  (SNORD-68) was used as the internal control (catalogue no.  MS00033712, Qiagen). The miRNA-specific primers [miR-  27a: catalogue no. MS00003241, Qiagen; and miR-18b: catalogue no. MS00031521, Qiagen] were used. SYBR Green  Master Mix (Qiagen/SABiosciences Corporation, USA) was  used in the (RT-PCR) reaction according to the manufacturer’s suggested protocol, along with the manufacturer-provided miScript Universal primer and miRNA-specific forward primer. |
| 9 | Habib | 2019 | qPCR | serum | The levels of lncRNA-TSIX and SOGA mRNA expression  in the serum of the participants were assessed using RT2  SYBR Green ROX qPCR Mastermix and a Quantitect SYBR  Green Master Mix Kit (Qiagen, USA), respectively, on a  Step One Plus™ System (Applied Biosystems Inc., Foster  City, CA) with specific primers (Accession: NR_003255 and  NM_199181, respectively) provided by Qiagen (USA). Beta  actin (Accession NM_001101) was used as a housekeeping  gene.miR-548-a-3p expression in the sera was investigated  by mixing the total cDNA with the reagent provided in an  miScript SYBR Green kit (Qiagen, USA) according to the  manufacturer;s suggested protocol, in addition to the manufacturer-provided miScript universal primer and miRNA specifc forward primer (hsa-mir-548-a-3pa-3p miScript Primer Assay) (Accession: MI0003593). RNU-6 was used as an internal control. All the PCR primers were obtained from Qiagen (USA).rom Qiagen (USA).  The PCR program for the SYBR Green-based qPCR was  as follows: denaturation at 95 °C for 15 min; 40 cycles of  denaturation for 10 s at 94 °C; then annealing for 30 s at  55 °C; and lastly, extension for 34 s at 70 °C. Each reaction  was performed in duplicate. The threshold cycle (Ct) value of each sample was calculated using the StepOnePlus™ software v2.2.2 (Applied  Biosystems). Any Ct value greater than 36 was considered  negative. We used the melting curve analysis software of  Applied Biosystems to analyse our results. The melting  curves were analysed to affirm the specifcities of the amplicons for the SYBR Green-based PCR amplification. The expression of the RNA-based biomarker panel was measured using the2^-△△Ct^Technique. |
| 10 | EI-Hamouly | 2019 | qRT‑PCR | Plasma | Real-time PCR was accomplished by TaqMan Universal  Master Mix II Kit (Applied Biosystems). Real-time PCR  reactions were done with a total volume of 20μL including1 μL TaqMan mi-RNA Assay, 2μL cDNA, 10μL TaqMan  Universal PCR Master Mix and 7μL RNAase free water.  miR-301 expression analysis was performed by the ABI  7500 Real-Time PCR instrument (Applied Biosystems) with  the subsequent cycles: initial phase of 95 °C for 10 min, then  40 cycles of 95 °C for 15 s and 60 °C for 60 s.  The RT and PCR primers for miR-301 (hsa-miR-301;  Assay ID 000528) and U6 (U6 snRNA; Assay ID 001973)  were supplied by (Applied Biosystems).  miR-301 expression value was expressed relative to that  of U6 (reference control gene) and calculated from the equation: relative expression=2^-△△Ct^ |
| 11 | EIhendawy | 2020 | RT-qPCR | peripheral venous blood | N/A |
| 12 | Piciocchi | 2013 | Real-time PCR | Plasma | The plasma cfDNA concentration was measured by using a reproducible quantitative real-time PCR reaction targeting the hTERT gene (Quantifiler Human DNA Quantification Kit, Applied Biosystems, Carlsbad, CA). The reaction was done in 96-well plates with a volume of 25μL/well, containing 12.5μL of Quantifiler Reaction Mix, 10.5μL of  Quantifiler Human Primers Mix and 2μL of DNA sample.  The reaction was performed in triplicate. A standard curve was added to every amplification plate using eightfold serial dilutions of genomic human DNA from 0.023 to 50 ng/lL, as designed by Quantifiler Kit  User^，^s Manual (Applied Biosystems). However, two additional serial dilutions at lower concentrations were added to the standard curve, which remained absolutely linear(0.003–50 ng/lL), as validated by additional experiments performed. The PCR was done at 95℃ for 10 min, followed by 40 cycles at 95℃ for 15 s and 60℃ for 1 min, using an ABI PRISM 7900HT Sequence Detection System. The results were analyzed with Software 2.3 (Applied Biosystems). The amount of hTERT gene in plasma served as the target of cfDNA and was expressed as ng/lL×100. |
| 13 | Yang | 2015 | qRT-PCR | serum | The extraction of total RNA from sera was carried out  using mirVana PARIS Kit (Ambion, Austin, TX, USA)  according to the manufacturer’s instructions. The RNA  concentration was measured using a NanoDrop 2000  spectrophotometer (Thermo Fisher Scientific, Wilmington,  DE, USA). cDNA was reversed from total RNA with  TaqMan MicroRNA Reverse Transcription Kit (Applied  Biosystems), following the manufacturer’s protocol.  Quantitative real-time polymerase chain reaction (qRTPCR) was measured using the miScript SYBR-Green PCR  Kit on the ABI 7500 Real-Time PCR System (Applied  Biosystems, Foster, CA, USA.) RNU6B was  used as internal control. The primers of miRNAs and U6  were purchased from RiboBio (Guangzhou, China). The  PCR amplification protocol was as follows: 95℃ for  10 min, 40 cycles of denaturation at 95℃ for 15 s,  annealing at 55℃ for 30 s, and extension at 60℃for  30 s. The relative expression of miR-218 was calculated using the  2^-△△Ct^ method . All the reactions were experimented in triplicate. |
| 14 | Huang | 2011 | Real Time PCR | Plasma | PCR was done in duplicate on a DNA Engineer Opticon II (Bio-Rad  Laboratories, Hercules, CA, USA). Each 20μL reaction  consisted of 1×SYBR Premix DimerEraser™(Takara, Dalian, China),  0.2μM of each primers, 200μM deoxynucleotide triphosphate mix, and 2 μL DNA sample. PCR was done at 95°C for 30 s, followed by 40 cycles at 95°C for 5 s, 58°C for 25 s, and 72°C for 10 s. Melting curve analysis was performed to confirm the specificity of PCR products. For construction of the calibration curve for each amplification, we  generated a standard curve using 5-fold serial dilutions of  human genomic DNA (10, 50, 250, 1,250, 6,250 pg). |
| 15 | Chen | 2015 | real-time PCR | Serum | Total RNAs from sera were extracted using the mirVana PARI  S Kit (Ambion, Austin, TX, USA) according to the manufacturer’s instructions. The RNA concentration was measured  with a NanoDrop 2000 spectrophotometer (Thermo Fisher  Scientific, Wilmington, DE, USA). Reverse transcription  was performed using the PrimeScript™ First Strand cDNA  synthesis kit (Takara Bio, Inc., Dalian, Japan) according to  the manufacturer’s instructions. Real-time PCR was performed using the All-in-One™ miRNA quantitation by real-time PCR (qRT-PCR) detection kit (GeneCopoeia, Rockville, MD, USA) on the Applied Biosystems 7500 Real-time PCR System (ABI, Abilene, TX, USA). The PCR amplification protocol was as follows: 95℃ for 10 min, 40 cycles of denaturation at 95℃ for 15 s, then 1 min of annealing/ extension at 60℃. The comparative cycle threshold (CT) method was applied to quantify the expression levels of miRNAs. The small nuclear U6 RNA was used as internal control. The relative amount of miRNAs was calculated using the equation 2^-△△Ct^. |
| 16 | Zuo | 2015 | qRT-PCR | Serum | In the first step, reverse transcription (RT)  assay was performed in a reaction volume of 15 μL, using  TaqMan® miRNA Reverse Transcription Kit (Applied  Biosystems, Foster City, CA), which contained 4.16 μL of  Nuclease-free water, 1.50 μL of ×10 Reverse Transcription  Buffer, 0.19 μL of RNase Inhibitor, 0.15 μL 100 mM dNTPs  (with dTTP), 1 μL of Multi Scribe Reverse Transcription,  3 μL of miRNA-specific 5×RT primer (Applied Biosystems), and 5 μL of total RNA by sequentially incubating at 16℃for 30 min, 42℃for 30 min, and 85℃ for 5 min.  The quantification of miRNA expression was done according  to the manufacturer’s standard instructions for the TaqMan®  miRNA assay on a CFX96 system (Bio-Rad, USA) for real-time PCR assay. Each reaction (20μL) included 1.33μL of  the reverse transcription products, 1.00μL of TaqMan® small  RNA assay (20×), 7.67μL of nuclease-free water, and  10.00 μL of TaqMan® Universal PCR Master Mix II (2×)  without UNG supplied by Applied Biosystems. All qPCR  reactions were analyzed in triplicate and were incubated in a 96-well plate with an initial enzyme activation at 95℃ for  10 min, followed by 40 cycles of 95℃ for 15 s and 60℃ for  1 min. The comparative CT method( 2^-△△Ct^) was used to calculate relative expression of miRNA with small RNA U6 as an endogenous reference for normalization . |
| 17 | Nasser | 2019 | (RT) PCR | serum | Purification of total RNA, including small RNAs (e.g, miRNAs),  from serum was carried out using the miRNeasy Mini Kit, Qiagen  (cat. no. 217004); followed by quantification of Serum miR-21,  miR-223, and miR-885-5p by quantitative reverse transcription  (RT) PCR with the TaqMan MicroRNA Assays (Catalogue number  4427975, 4370048, 4366596 Applied Biosystems) using 7500  Real-Time PCR Instrument, Applied Biosystems. Concisely, TaqMan  MicroRNA Assay included two steps; a reverse transcription (RT)  step which was carried out, using a stem-looped small RNA specific RT primer, with the following cycling conditions; incubation at 16℃ for 30 min, and 42℃ for 30 min, and 85℃ for 5 min in a thermal cycler. Subsequently, real time quantitative PCR with specific TaqMan probe [Minor Groove Binder (MGB)] and primers (forward and reverse) were performed with the following conditions: 95℃ for 10 min, succeeded by 40 thermal cycles of 95℃ for 15 s and 60℃ for 60 s. The Quantification cycle) (Cq) was automatically given by the software where the serum levels of the studied miRNAs were normalized to control miRNARNU58A and expressed as 2 ^-△cq.^ |
| 18 | Fouda | 2020 | qRT-PCR | serum | The relative expression of miRNAs in the serum samples was  determined using the miScript SYBR green PCR kit, according to  the manufacturer’s instructions (Qiagen, Germany). qRT-PCR using  the specific primers targeting the miRNAs was performed under  the following conditions: 95℃for 10 min, then 40 cycles at  95℃ for 15 s and 63℃ for 30 s. The RT-qPCR assay was performed  using ViiA 7 real-time PCR (Applied Biosystems, Foster City, CA,  USA). The samples were tested in duplicate. Data were analysed using the comparative method. The threshold cycle (CT) was determined for individual miRNA. MiRNA U6 was used as endogenous control. For measuring the △CT value,  the Ct numerical value of the endogenous control was substituted  from the Ct numerical value of the miRNA. Results were normalised against normal samples using the formula [△△ CT = △CT_tumour_-△ CT_normal_], the fold change (FC) was calculated using  the formula [FC = 2^-△△Ct^], and log FC was calculated |
| 19 | Li | 2019 | qRT-PCR | plasma | Quantitative real-time PCR (qRT-PCR) was performed in triplicated  with iTaq Universal SYBR Green supermix (Bio-Rad, Hercules, CA, USA) on a CFX96 system (Bio-Rad, CA, USA) following the manufacturer's instructions. GAPDH served as the internal control. Relative mRNA expression was calculated by the 2^-△△Ct^ method. QRT-PCR data processing was shown in an additional file. |
| 20 | Abdelgawad | 2015 | qRT-PCR | whole blood | A extraction was done from mononuclear cells using InviTrap Spin  Blood RNA Mini Kit according to manufacturer's instructions. Oneμg  RNA was reversely transcribed using high capacity cDNA reverse  transcription kit (Applied Biosystems). Reverse transcription was  performed in 20μl reaction containing 1×RT buffer, 0.2 mM dNTP  mixture, 1×RT random primer, 50 U multiscribe TM reverse transcriptase and nuclease free water. The reaction was performed at 25℃ for 10 min, followed by 37℃ for 120 min and 85℃ for 5 min then kept at 4℃.Primers designed for KIAA0101 detection were according to a previous report. Real-time quantitative PCR amplifications were performed in a total volume of 20μl; each reaction contained 2μl 10×buffer, 10μl 2×QuantiTect SyberGreen PCR master mix (Qiagen), 1μl (10μmol/l) of each primer, 2μl cDNA, and 5μl ddH2O.PCR regime involved a 95℃ 5-min initial denaturation step followed by 23 cycles (forβ-actin) and 28 cycles (for KIAA0101) at 95℃ for 30 s, 55℃ for 30 s, and 72℃ for 30 s. |
| 21 | Zhuang | 2015 | qRT-PCR | serum | Reverse transcription was performed using PrimeScriptTM RT  reagent Kit (Takara). Briefly, reverse transcription reaction  mixture contained 10 ng small RNAs, 10 mM of each dNTPs,  5×RT buffer and 0.5 L of miRNA-specific RT primer was  incubated at 37◦C for 15 min, and 85 ◦C for 5 s. Subsequently,  real-time quantitative PCR was performed using SYBR Green  PCR Master Mixture (Takara). The reaction mixture for qRT-PCR included 10 L 2×SYBR Premix, 0.8 L forward primer (10 M), 0.8 L reverse primer (10 M), 2 L reverse transcription product, 0.4 L Rox and 6 L ddH2O. All reactions were run on Stratagene Mx3000p system (Agilent Technologies) using the following conditions: 95 ◦C for 5 min,  followed by 40 cycles at 95 ◦C for 5 s, 60 ◦C for 30 s, and 72 ◦C  for 30 s. Cycle thresholds (Ct) were automatically set. The  relative expression levels of miRNAs were calculated using  △Ct method with cel-miR-39 and U6 as reference control  ( △Ct_miRNA_ = Ct_miRNA_—Ct_miR-39_—Ct_U6_) . Lower CtmiRNA  value means higher expression level of miRNA. |
| 22 | Lin | 2015 | qRT-PCR | Serum | Mature miRNAs were reverse transcribed using miScript II  RT kit (Qiagen), followed by quantitative real-time polymerase chain reaction (qRT-PCR) using miScript SYBR Green  Kit (Qiagen) in accordance with manufacturer’s instructions  on an Applied Biosystem 7500 Real-Time PCR System (Life  Technologies). The synthetic cel-miR-39 was used as exogenous internal control (for serum samples) as described by Mitchell et al. For quantification of miR-224 expression in tumorous tissues, U6 was adopted as internal control.qRT-PCR was performed in triplicate. Relative expression of miRNA was calculated using comparative Ct method and expressed as 2^-△Ct^ |
| 23 | Han | 2018 | qRT-PCR | plasma | The plasma were thawed and transferred into microcentrifuge tubes  (500μL of each plasma). 50 μL of artificially synthesized Cel-miR-39 with a concentration of 100 nmol/L was added into each plasma. Total RNA was extracted using the miRNeasy Mini kit (Qiagen). Target miRNA was reverse transcribed to cDNA by a gene-specific RT primer using the miScript. miRNA expression profiles of plasma were  determined with miScriptSYBR Green PCR Kit (Qiagen) and  performed on CFX96 Real-Time PCR System (BioRad). The expression of the target, normalized to a control, was calculated by the comparative Ct method. In order to ensure the reliability to normalize data, exogenous reference Cel-miR-39 was used as another internal standard control for normalization. The amplification procedure consisted of two initial holds at 50℃ for 2 min and 95℃ for 2 min; this was followed by 40 cycles made up of 95℃ for  15 s and 60℃ for 60 s. All RT reactions were performed in  triplicates. miRNAs expression was calculated using 2^−△Ct^. |
| 24 | Sun | 2019 | RT-qPCR | Serum | Reverse transcription was carried out using a TaqMan  MicroRNA Reverse Transcription Kit (Applied Biosystems)  according to the manufacturer’s protocol. PCR was subsequently carried out using the Roche 480 real-time PCR  system using a TaqMan MicroRNA assay (Applied Biosystems)  for the following reaction: 95℃for 10 min, 40 cycles of 95℃  for 15 s, and 60℃for 1 min. Cel-miR-39 was used as an external control gene and the 2^-△Ct^ method was employed to assess miRNA expression. |
| 25 | Amr | 2017 | RT-quantitative PCR | plasma | RNA was isolated using RNeasy Mini Kit (QIAGEN, Hilden,  Germany) according to the manufacturer’s instructions for  copurification of miRNA, then stored at -80℃. MicroRNAs  expression (miR-122 and miR-224) was determined by  applying the TaqMan MicroRNA Assays (Applied Biosystems,  Carlsbad, CA, USA). The extracted miRNA was reverse  transcribed in the reaction mixture containing miR-specific  stem-loop RT primers for each using Reverse Transcription  Kit (Applied Biosystems). Master Mix of TaqMan Universal  PCR without AmpErase UNG (Applied Biosystems) was  applied for real-time PCR. The reaction was run on an ABI  PRISM 7000 system (Applied Biosystems). The resulted  miRNA data are calculated in relative to RUN6B. All  samples were measured in duplicates and Relative quantity  (RQ) of miRNAs 122 and 224 was calculated by the formula  (RQ=2^-△Ct^) |
| 26 | Yang | 2017 | Qubit dsDNA 7 HS Assay kit | plasma | The plasma was stored at −80℃until use. The MagMAXTM Cell-Free DNA Isolation Kit (Life Technologies, USA) was used for the isolation of circulating-free DNA from 1ml plasma samples. For each sample, the level of extracted circulating-free DNA was quantified using Qubit dsDNA. |
| 27 | Tomimaru | 2011 | qRT-PCR | Plasma | Reverse transcription (RT) reaction and real-time quantitative RT-polymerase chain reaction (qRT-PCR) were performed using Taqman human miRNA assay kit (Applied Biosystems, Foster City, CA) according to the instruction supplied by the manufacturer. The expression of the target miRNA in the tumoral tissue and the non-tumoral tissue was normalized relative to the expression of RNU48, which was used as an internal control. On the other hand, there is no established endogenous plasma miRNA control for normalization of plasma miRNA levels. Therefore, in the present study, the expression of the target miRNAs in plasma was normalized relative to the expression of miRNA-16, which was confirmed to exist abundantly and stably in the plasma, as an internal control in previous reports. Data were analyzed according to the comparative Ct method (2 ^-△CT^) |
| 28 | Shaheen | 2018 | qRT-PCR | Serum | Reverse transcription (RT) was performed in a 15μl reaction volume using 5μl of RNA (10 ng per reaction), 3μl stem-loop RT primer and 7μl RT Reaction Master Mix using TaqMan MicroRNA Reverse Transcription Kit (Applied Biosystems, Foster City, CA, USA).The tube was incubated on ice for 5 min and loaded into the thermal cycler using the following conditions: 16oC for 30 min, 42oC for 30 min and 85oC for 5 min. For real-time PCR, 1.33 μl RT products were mixed with 10 μl TaqMan® Universal PCR Master Mix II, No UNG, 1μl TaqMan MicroRNA Assay and 7.67μl nuclease free water in a final volume of 20 μl according to manufacturer instructions. All reactions were run on StepOne real time PCR system (Applied Biosystems, USA) using the following conditions: 95℃ for 10 min, 45 cycles of 95℃ for 1s and 60℃ for 60 s. Relative expression of miRNA was calculated using the comparative cycle threshold (Ct) method. Fold change of each candidate miRNA within each group was then calculated using the equation 2 ^-∆∆CT^ |
| 29 | Luo | 2018 | RT-qPCR | Plasma | Quantitative real-time PCR assay was performed on the Bio-Rad CFX96 (Bio-Rad Laboratories, Inc., Hercules, CA, USA) using SYBR-green I Premix EXTaq following the instructions for users.  The reaction protocol was as follows: 95℃for 5 min, followed by 40 cycles of 95℃ for 30 s, 63.7℃ for 30 s, and 72℃ for 30 s. |
| 30 | Li | 2021 | qRT-PCR | serum | First, we used TRIzol reagent (TaKaRa, Otsu, Shiga,  Japan) and miRNA Purification Kit (CWBiotech, Beijing,  China) to extract total RNA from cells and serum samples according to the manufacturer’s instructions, and  used a microplate reader to detect the RNA concentration. Then we reversed transcription of miRNA by miRNA first-strand cDNA synthesis kit (Applied Biosystems, Foster City, CA). The reverse transcription sequences were as below: The qRT-PCR was carried out using the 7900real-time PCR system (Applied Biosystems, Foster City, CA) to perform on the processed samples, and detect their expression in the SYBR Green kit (GenePharma, Shanghai, China). The PCR condition was as follows:95℃ for 3 min, followed by 35 cycles of 95℃ for 20 s,55℃ for 30 s, and 72℃ for 30 s, and finally with 5 min extension at 72℃. U6 was selected as the internal standard to standardize its expression, and the relative expression of miR-487b was  calculated using the 2^-△Ct^ method. |
| 31 | ALrefai | 2023 | RT-PCR | blood | A miScript SYBR Green PCR kit provided by Qiagen was used for amplification. The cDNA samples were diluted 1:5 with nuclease-free water prior to amplification. A total of 12.5 µL SYBR Green Master Mix, 3.5 µL nuclease free water, 4 µL diluted cDNA, 2.5 µL miScript universal primer, and 2.5 µL miScript primer assay was used in a whole volume of 25 µL. The amplification was carried out in ABI 7500 real-time PCR (software version 2.0.1) as follows: initial activation at 95℃for 15 minutes followed by 40 cycles of 94℃ for 15 seconds, 55℃ for 30 seconds, and 70℃ for 30 seconds. The relative quantifications of miR-331-3p, miR-23b-3p, and miR-3194-5p were determined using the comparative 2^−ΔΔCt^ method after normalizing the expression levels of miR-331, miR-23b-3p, and miR-3194-5p to that of RNU6. |
| 32 | Zhao | 2021 | qRT-PCR | serum | Total RNA from cells and serum samples was extracted using TRIzol reagent (Invitrogen, Carlsbad, CA, USA) as per the manufacturer’s instructions. The concentration and purification of RNA were  spectrophotometrically confirmed by calculating the OD ratio (A260/280 close to 2.0) using a NanoDrop 2000 Spectrophotometer (Thermo Scientific, DE, USA). The RNA was then reversely transcribed into cDNA using a miRNA cDNA synthesis kit (CWBiotech, Beijing, China) following the manufacturer’s protocols. The expression of miR-324-3p was determined using a miRNA qPCR assay kit (CWBiotech, Beijing, China) and measured on an Applied Biosystems 7900 Real-Time PCR system (CA, USA) with setting as follows: 95℃ for 10 min, 40 cycles of 95℃ for 20s, 58℃ for 15s, 72℃ for 20s. The expression quantitation of miR-324-3p was done using the 2^−ΔΔCt^ method and normalized to the control cel-miR-39-3p |
| 33 | Chen | 2016 | qRT-PCR | Plasma | Real-time polymerase chain reaction (PCR) was carried out using iTaq Universal SYBR Green Supermix and CFX96 real-time PCR system (Bio-Rad, Hercules, CA, USA). The total reaction volume was 20 μL, which contained 2 μLcDNA, 10 μL SYBR Green Supermix, 1.6 μL  microRNA-specific forward and universal reverse primer (10 mM), and 6.4μL nuclease-free water. The PCR reaction was started at 95°C for 30s, then followed by 40 cycles of 95℃for 5 s, 57℃ for  30 s, and 72℃ for 30 s. All reactions were amplified in duplicate. Samples with Ct value >37 were considered to be unacceptable and discarded from the study. To normalize the plasma miR-125b  levels, we defined the small nuclear U6 RNA as endogenous control gene. The relative expression comparative Ct method formula 2 ^-ΔCt^ |
| 34 | Huang | 2020 | qRT-PCR | Serum | Total RNA was extracted from serum samples using  a Hipure Liquid RNA Kit (Cat# R4163-03, Magen, Guangzhou, China). The RNA quantity and purity were evaluated  via the NanoDrop One spectrophotometer (Thermo Scientific, Wilmington, DE, USA). The purified RNA was reversely  transcribed into cDNA using the M-MLV Reverse Transcriptase (Cat# M1701, Promega, Madison WI, USA). Then, the  levels of lncRNAs were assessed by qRT-PCR using TB  Green™ Premix Ex Taq (Cat# RR420A, Takara, Dalian,  China) which was performed on the QuantStudio Real Time  PCR system (Applied Biosystems, Foster City, CA, USA). All  reactions were performed with the following conditions:  95℃for 30 seconds, 45 cycles of 95℃for 5 seconds, and 60℃  for 30 seconds. The specificity of the PCR products was  ensured by melting curve analysis following each reaction.  The relative expression of each lncRNA was determined  using the 2^−ΔΔCt^ method with GAPDH as the endogenous  control for data normalization. |
| 35 | Miura | 2005 | qRT-PCR | Serum | RNA was extracted with DNase treatment from serum as reported  Previously. RNA from 200μL of serum was dissolved in 200μL of H_2_O. The quantitative reverse transcription-PCR (RT-PCR) was done by using 1μL of RNA extract and 2μL of SYBR Green I (Roche, Basel, Switzerland) in a One Step RT-PCR kit (Qiagen, Tokyo, Japan), in which h2-microglobin RNA was used as a PCR quality control. RNA was extracted from HCC tissues by using the same volume of serum and  dried up to 20-fold concentration. RNAs from HCC tissues were  extracted using TRIzol Reagent according to the instructions of the  manufacturer (Invitrogen Corp., Carlsbad, CA). The RT-PCR condition was an initial incubation at  50℃ for 30 minutes followed by a 12-minute incubation at 95℃，  then 50 cycles at 95℃ (0 second), 55℃ (10 seconds), and 72℃ (15 seconds), and a 20-second melting at 40℃.The PCR yielded products of 131 bp for hTERT, of 150 bp for AFP, and of 88 bp for h2-microglobin RNA, respectively (data not shown). The RT-PCR assay was repeated twice and the quantification was confirmed by using LightCycler (Roche) with reproducibility. |
| 36 | Shen | 2017 | RT-qPCR | Serum | Real-time PCR was performed using an ABI 7500 PCR Detection System (ABI, USA). The 20 µl of PCR reaction mix included 10 µl SYBR Green I mix, 3 µl cDNA, 1 µl forward primer, 1 µl reverse primer and 5 µl RNase-free H2O. Reactions were incubated in optical tubes at 95°C for 10 min, followed by 40 cycles of 95℃ for 15 sec and 60℃ for 31sec. All experiments were done in triplicate and date was calculated using the comparative Ct (2^−∆∆Ct^ ) method. U6 was used as an internal control to normalize RNA input in the real-time PCR assay. |
| 37 | Nguyen | 2022 | RT-PCR | Serum | hTERT mRNA was evaluated by Real-time PCR method  using SYBR Green. Serum AFP+AFP-L3+DCP were  assessed by TASWako^®^ i30 a clinical automated immunoanalyzer system using a micro-chip capillary electrophoresis  (Liquid-phase binding assay). Concentrations of AFP and  AFP-L3% were calculated on the basis of the peak region of  the fluorescence intensity of complex 1 (part AFP-L3) and  complex 2 (part AFP-L1). |
| 38 | Miura | 2010 | RT-qPCR | Serum | RNA was extracted with DNase treatment from serum as reported previously. The quantitative RT-PCR was performed as described previously. for hTERT. The RT-PCR condition was an initial incubation at 50 for 30 min followed by a 12-min incubation  at 95, then 50 cycles at 95 (0 s), 55 (10 s), and 72 (15 s),  and a 20 second melting at 40℃. The dynamic ranges of  real-time PCR analysis for hTERTmRNA were more than  approximately 5 copies in this assay and we were able to  exclude the possibility of false negativity in serum samples from patients with CH, LC and controls. The PCR yielded products of 143 bp for hTERT (data not shown). The RT-PCR assay was repeated twice and the quantification was confirmed by using LightCycler (Roche, Basel,  Switzerland) with reproducibility |
| 39 | Zhang | 2014 | RT-qPCR | Serum | The expressions of miRNAs were quantified by TaqMan  miRNA assays (Applied Biosystems) following reverse  transcription (Tiangen, Chin) of 40 ng RNA. Reactions  were loaded onto a 96-well plate and run in duplicate on an ABI 7900 Fast Real-Time PCR System (Applied Biosystems). The reactions were firstly incubated at 50℃ for 20 seconds and then 95℃ for 10 minutes, followed by 40 cycles of denaturation at 95℃ for 15 seconds, then  1 minute of annealing/extension at 60℃. TheΔΔCT method was used to determine relative number of copies (RQ) of miRNA. The U6 was chosen as the endogenous normalizer. |
| 40 | Han | 2022 | qPCR | Serum | Total RNA was extracted from equal volume of serum  samples with TRIzol LS reagent (Invitrogen, Carlsbad,  CA, USA) as described in the manufacturer’s protocol.  All RNAs were digested with DNase I (Takara, Dalian,  China). cDNA was generated using the First-strand  cDNA synthesize kit (#1622, Termo Fisher Scientifc Waltham, MA, USA) with random hexamer primers in  accordance with the manufacturer’s instructions. The  synthesized cDNA templates were further amplified by  SYBR Green master kit (Takara, Dalian, China), and the  expression of β-actin was used as the internal control |
| 41 | Yu | 2015 | qRT-PCR | Serum | The synthesized cDNA templates were further amplified by  SYBR Green master kit (Takara, Dalian, China), and the  expression of β-actin was used as the internal control.  We calculated the relative expression values by comparing the normalized cycle threshold (Ct). |
| 42 | Dhayat | 2015 | qRT-PCR | plasma | Quantitative Real-Time (qRT) PCR was performed using the miScript PCR system (Qiagen) as described previously. Quantitative microRNA analysis was performed using CFX Manager Software v2.1 (Bio-Rad Laboratories, Munich, Germany). Expression of circulating microRNA-141, microRNA-200a, microRNA-200b, microRNA-200c, and microRNA-429 was analyzed quantitatively after normalization to the cel-microRNA-39 spiked-in control using the ΔΔCt (cycle threshold) method. |
| 43 | Li | 2019 | qRT-PCR | Serum | The RT reaction was performed at 16℃ for 30min, 42℃ for 30min and 85℃ for 5 min by using the Applied Biosystems 7300 Sequence Detection System. qPCR was subsequently carried out using a TaqMan  MicroRNA assay (Applied Biosystems) for the following  reaction: 10μL of 2×Taq Manuniversal PCR Master Mix  II, 1μL of 20×TaqMan small RNA Assay reagent, 7.67μL  of nuclease-free water and 1.33μL of product from the RT  reaction. The qPCR was performed at 95℃ for 10 min, 40  cycles of 95℃ for 15 s, and 60℃ for 1 min. Fluorescence  readings were taken during the 60℃ step. A no template  control (NTC) and a control without reverse transcription  (NRT) were used as negative controls. To calculate the  relative expression levels of miR-122, cel-miR-39 was  used as a control miRNA and the 2^-ΔCt^ |
| 44 | Gharib | 2022 | qRT-PCR | Serum | The miScript SYBR Green PCR kit (Qiagen GmbH) was used for real-time quantitative reverse transcription PCR  (qRT-PCR), and reactions were carried out in duplicate, using the Stratagene Mx3005P-qPCR system. The endogenous  control miR-16 was utilized to standardize the expression levels of miRNAs isolated from serum samples.18 PCR  conditions were as follows: initial denaturation at 95°C for 15 minutes, then 40 cycles of 94℃ for 15 seconds, annealing  at 55℃ for 30 seconds, and extension at 70℃for 30 seconds. The primers were purchased from Qiagen GmbH.  Relative expression of the two miRNAs (miRNA-96-5p and miRNA-99a-5p) was calculated using the ∆∆CT method. |
| 45 | Qiao | 2019 | qRT-PCR | plasma | qRT-PCR was performed with SYBR Premix Ex TaqTM II  (TaKaRa, Dalian, People’s Republic of China) on  LightCycler 480II real-time PCR system (Roche) following manufacturer’s instructions. These primers were synthesized by Sangon Biotech (Shanghai, People’s Republic of China). The data were analyzed using the ΔCt method. |
| 46 | Quoc | 2018 | RT-PCR | plasma | cDNA synthesis was conducted with SensiFASTTM cDNA Synthesis Kit (Bioline, UK). The reaction of 20μL was as the following: 4μL5xTransAmp buffer, 1μL reverse transcriptase, 100 ng total  RNA and up to 20μL DNase/RNase free water. Reaction  conditions were 25℃ for 10 min, 42℃ for 15 min and 85℃  for 5 mins. RT-PCR for miRNA was performed with specific  primers of hsa-miR122 and GAPDH primers were used as  amplification control by GoTaq® Colorless Master Mix  (Promega, USA). Amplified PCR product of hsamiR122 was cloned into the pJET1.2 vector, followed with sequencing by FirstBASE company (Malaysia). |
| 47 | Wang | 2018 | RT-qPCR | plasma | According to manufacturer’s instructions, the expressions of GAS5-AS1 were performed on the Bio-Rad CFX96 (Bio-Rad Laboratories, Inc., Hercules, CA, USA) using SYBR Premix Ex Taq Kit (TaKaRa,  Japan). The reactions started at 95◦C for 5 min, followed by 40 cycles of 95℃for 30 s, 64℃for 30s and 72℃ for 30s. Ct values of the target GAS5-AS1were normalized in relation to GAPDH. The primers  were designed based on (NR_037605.1) GAS5-AS1 gene sequence obtained from the gene database of NCBI. The comparative Ct method formula 2^−∆Ct^ was used to calculate the relative gene expression. All experiments were carried out in duplicate for each data point. |
| 48 | Wang | 2023 | RT-qPCR | plasma | The reverse transcription (RT)reactions took place using the Prime Script™ RT Reagent Kit (Takara, Dalian, Liaoning) (Lot no. AK4802). The 20-ml solution contained five samples of 4-ml Prime Script Buffer Mix, 1 mg of template RNA and 1mg of Prime Script RT Enzyme Mix I, 1 ml of Oligo dT Primer and RNase-free H2O; it was then incubated firstly at 37℃ for 30 min, followed by 85℃ for 5 s, and finally 4℃ for 60 min. RT-qPCR was completed using a CFX96™ real-time system (BioRad, CA, USA). the total volume was 25 ml following the guideline by the manufacturer (Takara, Dalian, China). Then, the solution was incubated at 95℃ for 30 s, after that, 45 cycles of 95℃and 60℃ alternating for 5 s and 34 s, respectively. Three repetitions were the minimum standard for each test. The specificity of the RT-qPCR products was examined with the melting curve analysis. The relative gene expression level was normalized by the endogenous control-b-actin, then assessed using the  2^−△△Ct^ method. |
| 49 | Yousuf | 2022 | RT-qPCR | Serum | MiRCURY LNA™ SYBR Green PCR kit (#339346,QIAGEN, United States), commercially designed primers, cDNA, and nuclease-free water were used for amplification of the product. The desired master mix was prepared from the abovementioned reagents in a tube, and the obtained  mixture was mixed well by vortexing and spun down. The mixture was dispensed in tubes, and cDNA was diluted (1:60) in nuclease-free water and added to these tubes separately. The tubes were placed in a thermocycler (Rotor-Gene Q MDx, QIAGEN, working on Q-Rex  software, Germany) by following reaction conditions as  95℃ for 2-min, followed by 40 cycles of denaturation at  95℃ for 10-min, annealing at 56℃ for 1-min, and melting  curve at 60-95℃. U6 was used as an internal reference gene.  The ΔΔCt model was used for the relative quantification of  RT-PCR data of miRNA |
| 50 | Elfert | 2022 | qRT-PCR | Serum | For real-time PCR of each miRNA, 2.5μl diluted RT products were combined with 5.5μl RNase-free water, 10μl QuantiTect SYBR Green PCR Master Mix, and 2μl miScript universal primer (reverse  primer) and then added to a custom Rotor-Disc 100miRNA PCR array that contains miRNA-specific miScript primer assays (Qiagen, Valencia, CA). Optical thin wall strips were used to seal Rotor-Disc. The Rotor-Gene Q real-time PCR system (Qiagen, Valencia, CA) was used to run real-time PCR under the following settings: 95℃ for 30 min,  followed by 40 cycles of 15 s at 94℃, 30 s at 55℃, and 30 s  at 70℃. The cycle threshold (Ct) is the number of cycles  required for the fluorescent signal to cross the threshold in  real-time PCR. The fold change of miRNA expression levels  was calculated using the 2^-ΔΔCt^ formula |
| 51 | Eldosoky | 2023 | qRT-PCR | plasma | N/A |
| 52 | Boonkaew | 2023 | qRT-PCR | plasma | Additionally, the differentially expressed EV-miRNAs were validated in another set of plasma samples by quantitative Real-Time PCR (qRT-PCR) to identify novel biomarkers for NBNC-HCC. Finally, the diagnostic and prognostic roles of these potential biomarkers were analyzed. |
| 53 | Gao | 2018 | N/A | plasma | Total RNA was extracted using TRIzol reagent  (Invitrogen; Thermo Fisher Scientific, Inc.). First strand  cDNA was synthesized from 72 tissue or plasma samples using a PrimeScript RT Reagent Kit (Takara Biotechnology Co., Ltd., Dalian, China), according to the manufacturer's protocol; the reaction was performed at 16℃for 30 min, 42℃ for 30 min and 85℃ for 5 min. qPCR was performed to quantify the expression level of lncRNA with  SYBR Premix Ex Taq (Takara Biotechnology Co., Ltd.)  and the ABI Prism 7900HT Fast Real-Time PCR System  (Applied Biosystems; Thermo Fisher Scientific, Inc.). PCR  was performed at 95℃ for 2 min, followed by 40 cycles at  94℃ (15 sec), 60℃ (60 sec) and 72℃ (30 sec). All experiments  were carried out in triplicate. Relative expression levels were  calculated using the 2^-ΔΔCq^ method and were normalized  to GAPDH. |
| 54 | Zhao | 2018 | RT-qPCR | Serum | The mRNAiso Plus and microRNAiso Plus kits (Takara Biotechnology Co., Ltd., Dalian, China) were used for mRNA and miRNA extraction from liver tissue, respectively. The MiRCURY RNA kit  (Exiqon A/S, Vedbaek, Denmark), a specific kit for extracting  microRNA from the serum, was used to extract miRNA from  serum samples in order to guarantee the validation of the  concentration of microRNAs. RT‑qPCR for microRNAs  was performed on a 480 PCR system (Roche Diagnostics,  Basel, Switzerland), at 95℃ for 1 min followed by 45 cycles  of 95℃ for 5 sec, 65℃ for 30 sec and 72℃ for 30 sec. The  expression of miRNAs in serum samples was calculated using  the comparison 2^-∆∆Cq^ method relative to RNU6B (U6). |
| 55 | Li | 2014 | RT-qPCR | plasma | Quantification of mature miRNAs was accomplished by a two-step method. Firstly, RNA was 3'- extended with a poly(A) tail using poly(A) polymerase, then the poly(A) product was reverse transcribed using oligo(dT)-Universal Tag (Tiangen, China). Subsequently, real-time  quantitative PCR was performed with miRNA-139 primer and  internal normalization primer according to the procedures of  miRcute miRNA SYBR-Green qPCR detection kit (Tiangen)  in Mx3000p sequence detection system (Agilent, USA), using  the following conditions: 94℃ for a 2-min cycle, followed by  45 cycles of 94℃ for 20 sec, and 60℃ for 34 sec. Briefly, 20 µl  PCR system contained 2 µl of RT product solution, 10 µl of  2X miRcute miRNA Premix (including SYBR), 0.4 µl forward  primer, 0.4 µl reverse primer and 7.2 µl RNase-free ddH2O.  Triplicate PCRs were carried out for every cDNA sample,  including negative controls without templates. hsa-miR-U6  and hsa-miR-16 were used as the internal normalization  control for tissue and plasma sample respectively. All primers  were designed and provided by Tiangen. The expression level  of miRNA was computed using the comparative ΔCt method  as previously reported. |
| 56 | Xie | 2014 | RT-qPCR | Serum | N/A |
| 57 | El-Garem | 2014 | RT-qPCR | Serum | PCR quantification experiments were performed with  PCR (Applied Biosystems; Foster City, CA) using the  SYBR Green PCR Master Mix according to the manufacturer’s protocol. The primers for microRNA-122, -221 and housekeeping gene were supplied by Qiagene, Germany (catalog numbers 3416, 3857 and 33712).  The housekeeping miRNA SNORD68 was used as the endogenous control. Fluorescence measurements were made in every cycle and the cycling conditions used were: 95℃ for 30 s, and 40 cycles of 95℃ for 5 s and 60℃ for 34 s. Expression of miRNAs was reported as ΔCt value.  The ΔCt was calculated by subtracting the Ct values of miRNA SNORD68 from the Ct values of the target miRNAs. As there is an inverse correlation betweenΔCt and miRNA expression level, lower  ΔCt values were associated with increased miRNA. The resulting normalizedΔCt values were used in calculating relative expression  values by using 2^-ΔCt^, these values are directly related to the miRNA expression levels. The 2^-ΔΔCt^ method was used to determine relative-quantitative levels of individual miRNAs. |
| 58 | Wahb | 2021 | RT-qPCR | Serum | Purified miRNA was stored at -80°C until reverse transcription, which was accomplished using the Qiagen®miScript II RT Kit(QIAGEN) following the manufacturer’s instructions. Each 20-µl reaction tube  contained 4μL 5×miScript HiSpec Buffer, 2μL 10×miScript Nuclease Mix, 2μL.RNase-free water, 2 μL miScript Reverse Transcriptase Mix, and 10 μL template RNA.  Reverse transcription was carried out at 37℃ for 60 min and 95℃ for 5 min on an Applied Biosystems 2720 thermal cycler (Bioline, Singapore, United States). The cDNA product was diluted to 5 ng/ul before determining the transcript levels by real-time quantitative PCR. Real-time quantitative PCR was performed using the miScript SYBR  Green PCR Kit (QIAGEN) according to the manufacturer's instructions. The reaction mixture contained 12.5μL 2x QuantiTect SYBR Green PCR Master Mix, 2.5μL 10x miScript Universal Primer based on mRNA sequences obtained from the miRBase  database for miRNA 9-3p, 2.5μL template cDNA and 3.5μL RNase-free water. The Applied Biosystems®7500 real-time thermal cycler (Applied Biosystems, Foster City, CA, United States) was programmed to run 40 cycles of the following steps: 95℃ for  15 min (initial denaturation step), denaturation at 94℃ for 15 s, annealing for 30 s at 55℃ and extension for 30 s at 70℃. U6 snRNA was used as an endogenous control. Relative quantification expression levels were calculated using the comparative 2^−ΔΔCt^ method with Applied Biosystems 7500 software version 2.0.1. |
| 59 | Youssef | 2022 | qRT-PCR | plasma | The reverse transcription reaction was performed using the TaqMan™ MicroRNA Reverse Transcription Kit, Cat number # 4366596, (Applied Biosystems, Foster City, CA, United States) according to the manufacturer's instructions. miRNA-326, miRNA-511, and miRNA-424 quantification was carried out using quantitative real-time PCR (qRT-PCR) (Stratagene Mx3000p; Agilent Technologies, Germany). The qRT-PCR for each sample was carried out in duplicate using TaqMan 2x  universal master mix II (Applied Biosystems, Foster City, CA, United States) and TaqMan microRNA Assay Mix containing PCR primers and TaqMan probes for each miRNA. The expression level of RNU6B was used as an endogenous control for normalization. To determine miRNA relative expression, it was reported as a fold change (ΔCt and ΔΔCt calculations). |
| 60 | Nomair | 2020 | RT-PCR | Serum | Reverse transcription reaction was performed on 100 ng total RNA using a  miRNA Select Hi/LoRox kit according to the manufacturer’s instructions (BIOLINE). PCR amplification was performed after  the miRNA was reverse transcribed into complementary DNA (cDNA), and the reaction setup of quantitative PCR (qPCR) was prepared as follows: 10μl 2X SansiSMART PCR master mix, 2μl miRNA-224  primer (EPIK miRNA Select Hi/low-ROX Kit), and 5μl cDNA were mixed, then nuclease-free water was added to reach a final volume of 20μl. The housekeeping miRNA-39 was used as an internal reference. The  cycling conditions were: 1 cycle at 95℃ for 10 minutes and 40°C for 5 minutes, then 40 cycles at 95°C for 10 seconds and 60°C for 30 seconds. Raw Ct values were collected using the software  supplied with the real time PCR instrument (Applied  Biosystems 7000 Sequence Detector). The difference between the Ct values (∆Ct) of the target gene and housekeeping gene for each sample was calculated and the calibrated ∆Ct value (∆∆Ct) for each sample  was determined (∆∆Ct = ∆Ct of target gene -∆Ct of control samples). The relative gene copy number was calculated by the expression 2^-∆∆Ct^ . |
| 61 | Moshiri | 2018 | N/A | Serum | Four miRNAs, which emerged in a preliminary RNAseq  experiment, were validated using a droplet digital PCR  (ddPCR) approach on independent cohorts of patients and  controls. |
| 62 | Lou | 2022 | qRT-PCR | Serum | Total RNA was extracted using the miRcute miRNA isolation Kit  (DP501; Beijing, China) according to the manufacturer’s  protocol. Briefly, complementary DNA (cDNA) was synthesized using the TIANGEN lnRcute lncRNA cDNA kit  (KR202; Beijing, China). One hundred ng of total extracted  serum RNA was converted into cDNA. The relative expression of various genes was examined using the SYBR Green  Realtime PCR Master Kit (QPK-201; Shanghai, China) and  a PCR ABI 7500 Sequence Detection System. Each reaction mixture consisted of 2 µL of cDNA, 12.5 µL of mastermix and 1 µL of each primer, with the total volume adjusted to 25 µL using RNase-free water. The housekeeping gene18S was used as the internal control. Primer sequences for the lncRNAs HOTAIR, BRM and ICR, and for 18S mRNA were designed and synthesized by the HangZhou Adicon  Clinical Laboratory. |
| 63 | Farag | 2018 | RT-qPCR | Serum | PCR was performed using Dream Taq Green PCR Master Mix (Thermo Scientific, Fermentas). The temperatures protocol of reaction  was performed at 25ÚC for 10 min then 120 min respectively, and 85ÚC for 5 min and kept at 4ÚC.The reaction for â actin was performed in 25 µl reaction containing 1 µl cDNA, 1x master mix, 25 pmole of each primer. For amplified â actin, the cyclic condition consisted of initial denaturation at 94℃for 5 min, followed by 34 cycles of denaturation at 94℃ for 1 min, annealing at 63℃ for 2 min and elongation at 72℃for 3 min For amplified Glypican-3, the cyclic condition consisted of initial denaturation at 94ÚC for 5 min, followed by 35 cycles of denaturation at 94℃ for 30 sec, annealing at 58℃ for 45 sec, elongation at 72℃ for1 min and final elongation at 72℃ for 10 min. The PCR products were visualized on 2% agarose and were 452 bp for b-actin and 256 bp for target gene of Glypican-3 |
| 64 | Shehab-Eldeen | 2019 | RT-qPCR | Serum | nucleic acid extraction was done by QIAGEN viral RNA Mini Extraction Kit. Serum AFP was done by enzyme-linked immunosorbent  assay (ELISA) using IMMULITE 1000 system by a kit  supplied by Siemens Medical Solutions Diagnostics, USA  (Gitlin, 1975).MicroRNA was first effectively isolated from plasma  by using kits supplied by Qiagen™ RNA extraction kit  MiRNeasy kit, according to manufacturer instructions.  Once microRNA has been purified it needs to be  quantified by using NanoDrop® N50 nanophotometer  Implen GmbH and Implen, Inc. Schatzbogen 52 81829  München, Germany to determine both RNA concentration  and purity. The extracted microRNA product was stored  at–80°C for reverse transcription step. The extracted  microRNA was reverse transcribed (RT) to synthesize  single-stranded cDNA using the Qiagen®miScript II RT  Kit. RT master mix was prepared by adding the following  components to a nuclease-free microcentrifuge tube: 4μl  5×miScript HiSpec Buffer, 2μl 10×miScript Nuclease Mix,  2μl RNase-free water, 2μl miScript Reverse Transcriptase  Mix, then a 10 μl Template RNA was added to each a  20-µl reaction tube containing reverse-transcription master  mix. Reverse transcription was carried out at 37°C for 60  minutes and 95℃for 5 minutes on Applied Biosystems  2720 thermal cycler (Bioline, Singapore, USA) cDNA product was diluted to 5 ng/ul for determining transcript levels by real-time quantitative PCR |
| 65 | Aboelwafa | 2021 | qRT-PCR | Serum | Total RNA including microRNA was immediately isolated from plasma samples using miRNeasy Mini Kit(Qiagen, Maryland, USA) according to manufacturers’ instructions. The concentration and purity of the extracted total RNA were assisted using NanoDrop2000 Spectrophotometer (Thermo Scientific, USA). Singlestranded cDNA was synthesized from purified RNAsamples using the Taqman miRNA reverse transcription  Kit (Applied Biosystems, USA) for RNA reverse transcription according to the manufacturer’s protocol. The real-time amplification was performed using TaqMan MicroRNA assays for miR-331-3p, miR-23-3p, and TaqMan Fast Advanced Mater Mix (Applied Biosystems, USA) on the RotorGene Q Real-Time PCR System (Qiagen, Germany). RNU6 was used as endogenous references; its expression was stable in all the samples and independent of the analyzed variables. The relative expression levels were determined using the 2^−ΔΔCT^ method |
| 66 | Cimentepe | 2021 | qRT-PCR | Serum | miRNA expression levels were quantified in qRT-PCR systems by using miScript SYBR Green PCR kit according to the manufacturer’s protocols (Qiagen, Germany). RT-qPCR mixture with 20μl final volume contained 10μl 2x QuantiTect SYBR Green PCR Master Mix, 2μl 10x miScript Universal Primer, 2μl 10x miScript Primer Assay, 5μl diluted  cDNA, and 1μl RNease free water. All RT-qPCR analyses were performed using Rotor-Gene Q (Qiagen, Germany) with the cycling conditions were 95℃ for 15 min followed by 40 cycles of 94℃ for  15 sec, 58℃ for 30 sec, and 72℃ for 30 sec |
| 67 | Farag | 2018 | RT-PCR | Serum | PCR was performed using Dream Taq Green PCR Master Mix (Thermo Scientific, Fermentas). The temperatures protocol of reaction  was performed at 25ÚC for 10 min then 120 min respectively, and 85ÚC for 5 min and kept at 4ÚC.The reaction for â actin was performed in 25 µl reaction containing 1 µl cDNA, 1x master mix, 25 pmole of each primer. For amplified â actin, the cyclic condition consisted of initial denaturation at 94℃ for 5 min, followed by 34 cycles of denaturation at 94℃ for 1 min, annealing at 63°C for 2 min and elongation at 72°C for 3 min For amplified Glypican-3, the cyclic condition consisted of initial denaturation at 94ÚC for 5 min, followed by 35 cycles of denaturation at 94℃ for 30 sec, annealing at 58℃ for 45 sec,  elongation at 72℃ for1 min and final elongation at  72℃ for 10 min. |
| 68 | Gibriela | 2022 | qRT-PCR | plasma | QRT-PCR was carried out using the GoTaq  RqPCR Master Mix (Promega, USA, Cat No A6001) using  the StepOne Plus (Applied Biosystems, USA). To ensure accurate quantitation for investigated miRNAs and to avoid non-specific amplification for the designed primers, melt curve analysis was conducted after each PCR run. The 2^−∆∆Ct^ formula was used to determine  relative miRNA expression |
| 69 | Hussein | 2022 | RT-PCR | Serum | Serum samples from all participants were obtained for detection and  quantification of the miRNA using reverse transcriptionpolymerase chain reaction (RT-PCR). |
| 70 | Shehab-Eldeen | 2023 | RT-qPCR | Serum | Total RNAs and miRNAs were extracted directly from the whole  blood samples with the miRNeasy® Mini Kit (QIAGEN, Germany)  using the QIAzol-chloroform extraction procedure. Subsequently,  the yield and quality of total RNA and miRNAs were assessed using a Nanodrop instrument (Thermo Scientific, USA), and tubes  were then transferred to −80°C until the next step of the reverse  transcription stage: forming complementary DNA (cDNA) using the  miScript II RT Kit (QIAGEN, Germany). Each reaction was carried  out on ice with a total reaction volume of 15μL: 4μL of miScript  HiSpec RT buffer, 2μL of the miScriptTM reverse transcriptase, 2μL of miScript Nucleics Mix, and 10 ng of RNA extract in a maximum volume of 5 μL. Incubation was  performed in a 2720 Biosystems thermal cycler (Singapore) for one  cycle of 37℃ for 60 minutes and 95℃for 5 minutes to inactivate  the reverse transcriptase. The cDNA product was stored at −20℃  until real-time PCR.io of 1:5 with nuclease-free water. The reaction mixed preparation was carried out with 12.5μL of SYBR Green Master Mix,4μL of diluted cDNA, 2.5μL of miScript universal primer, and  2.5μL of miScript primer, and 3.5μL of nuclease-free water.  The miRNA RNU6 was used as an endogenous control. Mature  miRNA was detected with the aid of the miScript primer assay.  The data were analyzed using an ABI 7500 real-time PCR instrument under the following cycling conditions: initial activation step  at 95℃ for 15 minutes followed by 40 cycles (15 seconds at 94℃,  30 seconds at 55℃, and 30 seconds at 70℃). The expression levels of miRNA 29a-3p and miRNA 124-5p were normalized to the control-related endogenous model RNU6and calculated using the comparative 2^− △Ct^ method |
| 71 | Xu | 2018 | RT-qPCR | Serum | We used ABI Prism 7900HT sequence detection system (Applied  Biosystems, Foster City, CA) to carry out real-time quantitative PCR (qPCR) quantification of serum microRNA-125b with SYBR Green PCR  Master Mixture (Takara). The specificity of PCR product was validated by melting curve analysis when PCR cycles ended. For quality control  of qPCR, each sample was in duplicate and a negative control. The cycle threshold (Ct) is defined as the number of cycles required for  the fluorescent signal to cross the threshold in qPCR. Levels of serum microRNA-125B was calculated by the formula 2^-ΔCt^. |
| 72 | Zuo | 2014 | RT-qPCR | Serum | After collection of blood specimens in non-anticoagulant tubes, serum was separated by centrifugation at 3000 r/min for 5 min and stored at -80℃. Then 200 μL of serum was used for the extraction of miRNA according to the instructions of mirVana^TM^ miRNA Extraction Reagent. cDNA was synthesized by reverse transcription of the extracted RNA according to the instructions of TagMan@miRNA Reverse Transcription Reagent. The reverse transcription mix was prepared according to the ratio, and TagMan® hsa-miR-125b primer (5x) or the internal reference TaqMan® U6 sn-RNA primer (5x) were added respectively, and reverse transcription was performed using an RT-PCR instrument. After CDNA was synthesized, real-time fluorescence quantitative PCR was performed according to the instructions of TaqMan® miRNA qPCR reagent. TaqMan® Uni-versalPCR Mix Ⅱ (without UNG) was added, and TaqMan® hsa-miR-125b primer (20x) or internal reference TaqMan® U6snRNA were added. The specific primer sequences of miR-125b and U6 are shown in Table 2. The data were analyzed according to the amplification and melting curves of miRNA., |
| 73 | Song | 2020 | qRT-PCR | Serum | Total RNA of lncRNA-PVT1was extracted from serum samples using an RNA isolation kit (Axygen Science Inc., USA). Three replicates were performed for each gene in each sample. 1 μg of total RNA was used as a template to synthesize single-stranded cDNA using a reverse transcription kit (Genecopoeia, Guangzhou).Glyceraldehyde-3 phosphate dehydrogenase (GAPDH) was used as an endogenous reference, and a 20 μL qRT-PCR system (Genecopoeia, Guangzhou, China) was used for 40 cycles of amplification according to the following procedures: 95℃ for 1 min, 60℃ for 1 min, and 72℃ for 1 min, and the results were normalized to the endogenous control. The relative expression of IncRNA-PVT1 was measured using the 2-AAG method. |
| 74 | Lyu | 2021 | qRT-PCR | Plasma | Total RNA of exosomes in plasma was extracted with the exoRNeasy Serum/Plasma Midi Kit (Cat.77044, QIAGEN, Germany) according to manufacturer’s instructions. The free circulating RNAs in plasma were extracted by miRNeasy Serum/Plasma Kit (Cat. 217184) according to illustrations. Total RNA of HCC tissues was isolated  by the EastepTM Super Total RNA Extraction Kit (Cat. LS1040, Promega, WI, USA) following the instructions A fixed amount of RNA was reverse-transcribed to cDNA using an GoScript Reverse Transcription Mix kit (Cat.A2800) according to the instructions.  The quantitative real-time PCR (qRT-PCR) amplification of cDNA was analyzed by Tli RNaseH Plus (Cat.RR420B, Takara, Japan) using an Applied Biosystems 7500 Real-Time PCR System (Thermo Fisher Scientific, MA, USA). The primers details were available in supplementary materials (Supplementary Table 2). The amplification  conditions were set up as follows: 95℃ for 5 min, followed by 40 cycles of 95℃for 15 s, 62℃ for 40 s. Melting curve analysis was used to confirm amplification specificity. The amplification products of circRNAs were sent to the BioSune Biotechnology (Shanghai, China) Co. Ltd for RNA sequencing to verify their specific back splicing  sites. The 18S rRNA was considered as control, and the 2 (−△Ct) method was used to evaluate circRNA levels. |
| 75 | Wang | 2018 | RT-qPCR | Serum | MicroRNA expression in serum exosomes from all subjects  was assessed by RT-qPCR. For cDNA synthesis,  TaqMan microRNA Reverse Transcription Kit and TaqMan  microRNA-specific primers (Applied Biosystems, CA, USA)  were used following the manufacturer’s protocol. Reverse  transcription was conducted in a scaled down reaction  volume of 7.5μL, including 2.08μL of RNase-free water,  0.075μL of dNTPs with dTTP, 0.75μL 10× RT buffer,  0.5μL of multiscribe reverse transcriptase, 0.095μL of  RNase inhibitor, 1.5μL of microRNA-specific stem-loop  RT primer, and 2.5μL (25 ng) of total RNA template.  Reverse transcription reaction was then performed under  the following conditions: 16℃ for 30 min, 42℃ for 30 min,  and 85℃ for 5 min. Thereafter, TaqMan microRNA assay (Applied  Biosystem) was adopted to quantify individual exosomal  microRNA as described previously. Briefly, 20μL  reaction system was prepared as follows: 10μL TaqMan  Universal PCR Master Mix, 1μL TaqMan microRNA assay  mix, and 1.33μL of RT products, and 7.67μL nucleotidefree water. qPCR was performed on ABI PRISM 7300  system at the following condition: 50℃ for 2 min, 95℃  for 5 min, and 40 cycles of 95℃ for 15 sec and 60℃  for 1 min. All qPCR reactions were conducted in duplicate, and the Ct values greater than 35 were defined as 35. The expression levels of microRNAs were calculated by the 2^−ΔCt^ method as described previously |
| 76 | Wang | 2021 | qRT-PCR | Serum | Equal amounts of RNA were reverse transcribed with random  primers using Quantscript RT Kit (Tiangen, Cat: KR103).  Quantitative real-time PCR (qRT-PCR) of synthesized  complementary DNA (cDNA) was performed on an Applied  Biosystems 7500 System (ThermoFisher Scientific) using TB  Green® Premix Ex Taq™ II (Takara, Cat: RR820). |
| 77 | Wei | 2022 |  | Serum | Reverse transcription was performed by using One Step Prime Script miRNA cDNA Synthesis Kit (TaKaRa) The primers were designed and  synthesized by Invitrogen (Carlsbad, CA, USA). The reverse primers were provided by the One Step Prime Script miRNA cDNA Synthesis Kit (TaKaRa). Three replicate wells were set for each sample, and the  reliability of the PCR results was evaluated by the dissolution  curve. The relative expression of the target gene was calculated  by 2^-ΔΔCt^ |
| 78 | Chen | 2022 | qRT-PCR | Serum | Real-time quantitative fluorescent RT-PCR (qRT-PCR) was  performed using the SYBR Premix Ex Taq (Takara) kit  on the ABI 7500 detection system. The cel-miR-39 was  taken as the internal reference. The primers of  miR-34a and cel-miR-39 were purchased from  Guangzhou Ruibo Biotechnology Co., Ltd. |
| 79 | Ghosh | 2020 | qRT-PCR | Serum | cDNA was quantified using SYBR Green PCR Master Mix (Roche  Diagnostics, Basel, Switzerland) and gene-specific primers  in the QuantStudio 7 Flex RealTime PCR system (Thermo Fisher Scientific, Waltham, Massachusetts). RNU6B (tissue), C. elegans mir-39  (exosomes) and 18 seconds rRNA (tissue) were used as the internal  control for miRNA and mRNA. Log_2_ 2^−ΔCt^ where ΔCt [(Ct_Gene_ −  Ct_Internal Control_)×10^6^ was used for expression analysis. |
| 80 | Xu | 2018 | qRT-PCR | Serum | The primer and probe sequences for ENSG00000258332.1,  LINC00635 and GAPDH mRNA were designed using Primer Express v3.0 software (Applied Biosystems Co., Ltd, USA) and synthesized by Shanghai Huirui Biology Science & Technology Company. The PCR mixture contained 10μL of 2×PCR buffer, 0.4μL of DNA polymerase, 0.4μL of reverse transcriptase, 0.4μL of each primer, 0.8μL of each probe, 2μL of RNA template and nuclease-free water to a total volume of 20μL (TaKaRa Inc., Dalian, China). PCR was performed  in an ABI Prism® 7500 Sequence Detection System (Applied Biosystems Co, Ltd, USA) under the following cycling conditions: 50℃ for 15 min, 95℃ for 5 min, followed by 45 cycles of  95℃ for 15 s and 60℃ for 40 s. GAPDH mRNA was used as an internal control; no-template control was included as negative control. For quantitative results, the relative expression level expression of each lncRNA was represented as a fold change using the 2^-ΔΔCt^ method. Each  sample was assayed in duplicate, with an average of the two duplicates used for analysis. |
| 81 | Xu | 2017 | qRT-PCR | Serum | The primers and probes for exosomal hnRNPH1 mRNA and GAPDH  mRNA amplification were designed using Express v3.0 software  (Applied Biosystems Co., Ltd, USA), and synthesized at Shanghai Hui-rui Biological Technology Co., Ltd. The reaction system  comprised 10 μL of 2× PCR buffer, 0.4 μL of DNA polymerase II, 0.4 μL of reverse transcriptase mixture, 0.4 μL of each primer, 0.8μL of probe, 2μL of RNA template and deionized water to the total volume  of 20μL (TaKaRa Co., Ltd, China). The ABI Prism® 7500 PCR instrument (Applied Biosystems Co., Ltd, USA) was used for detection.  The following reaction conditions were used: the first stage (50℃  for 15 min), the second stage (95℃ for 5 min), and the third stage,  including 45 cycles (95 ℃ for 15 s, 60℃ for 40 s). The relative expression level of hnRNPH1 mRNA was calculated using the 2^−ΔCt^ method |
| 82 | Yang | 2022 | qRT-PCR | exosomes/Plasma | Total RNA was transformed into cDNA using Taqman MicroRNA Reverse Transcription Kit (Thermo Fisher Scientific), according to the manufacturer’s instructions. Quantitative PCR was then performed using Taqman microRNA assays on ABI Prism 7900HT Detection System (Thermo Fisher Scientific), as per the manufacturer’s instructions. The PCR cycling conditions were as follows: 1) 50℃ for 2 min; 2) 95℃  for 10 min; 3) 40 cycles of 95℃ for 15 s, 60℃ for 1 min. The  primers and probes were designed and produced by Thermo  Fisher Scientific: U6 snRNA (assay ID 001973), has-miR-29c  (assay ID 000587), has-miR-26a (assay ID 000405), and hasmiR-199a (assay ID 000498). U6 was used as a stable endogenous  control for normalization. The relative gene expression was  calculated as the value of 2^−ΔCq^. |
